# The Welfare of Dogs as an Aspect of the Human–Dog Bond: A Scoping Review

**DOI:** 10.3390/ani14131985

**Published:** 2024-07-05

**Authors:** Peter Verbeek, Chase Alan Majure, Laura Quattrochi, Stephen James Turner

**Affiliations:** Department of Anthropology, University of Alabama at Birmingham, 402 10th Avenue South, Birmingham, AL 35294-1241, USA; cmajure@uab.edu (C.A.M.); laura.quattrochi@gmail.com (L.Q.); sturner0@uab.edu (S.J.T.)

**Keywords:** human–dog bond, dog welfare, behavior

## Abstract

**Simple Summary:**

The popular notion that dogs are our best friends suggests a positive bond between ourselves and our dogs. When people think and talk about their connection with dogs, they often do this from a human perspective, and they are less likely to try to think about this from the dog’s perspective. We are interested in what the bond between humans and dogs means for dogs, and we are particularly interested in how a bond with a human partner affects the welfare of the dog. We decided to investigate to what degree and how research on the human–dog bond considers the welfare of dogs. We used a large database to select research publications on the human–dog bond published during the 2012–2023 period. We found 706 publications on the human–dog bond from around the globe, of which 246 had a research focus on dog welfare. We studied the publications with a focus on dog welfare, and we found that the characteristics and backgrounds of the dog and the owner affected both the nature of their bond and the welfare of the dog in both positive and negative ways. Most the publications that we studied were on pet dogs in Western industrialized societies.

**Abstract:**

The close bond that can exist between humans and their dogs is an important aspect of the evolutionary, economic, and social connections between the two species. There is a need for a better understanding of the place of the dog within the human–dog bond and on ways the human–dog bond affects dog welfare. We conducted a scoping review to investigate to what extent and in what ways dog welfare is addressed in the research literature on the human–dog bond. We identified 706 publications on the human–dog bond from across the globe that were published from 2012 to 2023. We found that 246 of these 706 publications had a focus on dog welfare. Our review showed that the interplay of characteristics and backgrounds of owners/handlers and their dogs was linked to dog welfare in multiple, both positive and negative, ways. Our review is limited by the fact that most of the research that we reviewed involved pet dogs and in majority came from Western, Educated, Industrialized, Rich, Democratic (WEIRD) societies. There is a need for a better understanding of how the human–dog bond affects the welfare of working, assistance, and service dogs.

## 1. Introduction

### 1.1. A Tale of Two Dogs

Years ago, when the first author lived in Bretagne, France, he heard from a friend that a local hunter was planning to shoot and kill his male Gordon Setter for being unsatisfactory in the hunt. The dog would not point toward the game that the hunters were chasing, but rather would drive the game away from the hunters before they had a chance to aim their guns at it and shoot it. As an alternative to killing his dog, the hunter let it be known that he would give away the dog to anyone who wanted it, and so I (PV) got in touch with him and told him that I would take his dog. For the next several weeks, I collected the dog, named Sulky, every afternoon from the garage in which he spent his days, and then took him for a long walk along the Bretagne shoreline. I thought that this might help Sulky to become accustomed to me and help him with his transition from his current life to life with me. Sulky never showed fear or aggression toward me when I arrived at the garage to pick him up, and he would let me leash him with no problem. During our walks together, he mostly ignored me, however, and he seemed not interested in being petted by me, nor did he pay much attention to me when I talked to him. One day when we had arrived at a remote spot along the coast with no people or other dogs in sight, I decided to take a chance and I unhooked Sulky’s leash from his collar. Sulky took off like a greyhound at a dog racetrack. I sat down on a rock overlooking the ocean and contemplated that this was perhaps not the smartest move that I had ever made in my life with dogs, releasing a powerful hunting dog in a remote area teeming with small mammals, quail, and even the occasional pheasant. Scent heaven! Then, just when I was trying to think of what to do next, a small black dot appeared on the rugged horizon, from behind some mighty granite rocks, gradually morphing into a large Gordon Setter, black feathery fur flowing in the ocean breeze, running toward me. When he finally made it to my side, Sulky sat down close to me—panting heavily and drooling profusely—and then put his head on my knee. He let me pet him and appeared to enjoy it, and he listened intently to my repeated “Good boy!” The next day, I took Sulky in for good. During the following years, my bond with Sulky was as close as with any of my other canine companions that I have been fortunate to share my life with thus far.

We were reminded of Sulky’s entry in the first author’s life by a recent news report about a politician in the US who shot and killed her 14-month-old German Wirehaired Pointer, Cricket, for reportedly ruining a pheasant hunt, being aggressive toward her owner, and for getting into a chicken coop with fatal consequences for the chicken. The news generated many comments from across the political spectrum [1], few of them positive.

We can hypothesize that both Cricket and Sulky behaved in part as a function of strong prey drives, fueled by their evolutionary heritage as a predator species, and shaped by generations of human-controlled selective breeding. We can also hypothesize that being well—feeling well—for Sulky may have meant running free, tracking scents and the animals that produced them in the Bretagne countryside, while for Cricket, it may have meant having a go at those cooped up chickens. As for Cricket’s purported aggressiveness toward her owner, we cannot say much about this, other than that it would be relevant to know more about Cricket’s owner’s behavior toward her, and about the way Cricket was raised from puppyhood. 

For both Sulky and Cricket, we can ask whether they should have been judged that harshly by their owners, or even judged at all, not to mention punished, for ruining hunts in which they were recruited to participate by their owners, and for which they were expected by their human minders to behave in certain ways and not in others. While a prey drive is inherent to a dog, human control over it is a training issue. Training creates a bond between the human and the dog, for better or for worse, and in the case of Sulky and Cricket, this was associated with an ultimate threat to the dog’s welfare; Sulky escaped with his life, Cricket did not. Considering this tale of two dogs implies what concepts such as welfare and wellbeing of a dog may mean to the dogs themselves, and how that connects to the human–dog bond. As a step toward a better understanding of this issue, we set out and report here on a scoping review that explores to what extent and in which ways dog welfare is considered in research on the human–dog bond. 

### 1.2. The Human–Dog Connection

There is a growing understanding that dealing with global environmental challenges requires a systems perspective that places humans squarely among other animals and considers mutualistic as well as antagonistic interactions between humans and other than human animals (hereafter animals) [2]. An integral aspect of such an approach needs to be a focus on interactions between humans and domestic animals, some of which can be described as mutually beneficial and others as exploitative when most benefits accrue to humans. A key connection that combines mutually beneficial as well as exploitative elements consists of the evolutionary, economic, and social links between humans and dogs, which is the oldest established connection between humans and another animal species (Ibid.). Leaving aside for a moment the importance of including this connection in a systems approach to dealing with the challenging environmental conditions that we have created for ourselves, it can be argued that if we truly want to understand ourselves as a species, we need to consider our evolved and developing connections with dogs, considering that dogs have been with us almost every step of the way. 

Science is heeding the call, as research on the connections between humans and dogs is rapidly becoming a major interdisciplinary field [3]. Many questions about the complex connections between humans and dogs remain to be answered, including about the processes of domestication of dogs and whether the Eurasian grey wolf, *Canis lupus*, is indeed the direct ancestor of the domestic dog, *Canis lupus familiaris* [2,4]. A better understanding of the evolution and domestication of dogs is important for guiding research on the human–dog bond. The human–dog bond is integral to the comprehensive connection between humans and dogs, and, considering the diversity in cultural opportunities and constraints on the human–dog connection across the globe, the human–dog bond can be expected to be highly varied in its expression and needs a multifaceted approach. 

#### 1.2.1. The Human–Dog Bond

It is important at this point to distinguish between the comprehensive human–dog connection and the human–dog bond. We define the human–dog connection as a complex network of human associations with dogs, ranging from puppy mills to search and rescue dogs, and from pampered pets to free-ranging street dogs, and much more. We follow Oxford Languages in defining a bond in the social realm as “a relationship between people or groups based on shared feelings, interests, or experiences”, and apply this definition to the close relationship between humans and dogs. As the sharing of feelings, interests, or experiences between humans and dogs is culture- and context-dependent, the human–dog bond can be expected to be highly varied in its expression. At the most basic level, research on the human–dog bond focuses on behavioral and cognitive interactions between humans and dogs that enable individuals or groups of the two species to coordinate their lives. Important work in this area is conducted on what dogs bring to the table for this, including ways in which dogs read and anticipate human behavioral cues, and how human language can function as a conduit for interactions between humans and dogs [5,6]. 

The ways in which dogs rely on and express their species-specific ability to attune to human behavior and language can also be expected to vary significantly across social and cultural contexts, and this has inspired ethnographic and culturally informed research on the form and function of the human–dog bond across the globe [7]. The questions here are how and why and what kinds of feelings, interests, or experiences are shared between humans and dogs, and in what contexts? The complexity of the task of understanding these contextually defined close connections between humans and dogs is apparent from the fact that the world population of dogs is estimated at between 700 million and 1 billion dogs, 70% of which are free-ranging dogs [8]. To date, much of the research on the human–dog bond as an aspect of the comprehensive human–dog connection has been on owned and non-free-ranging dogs, primarily in Western, Educated, Industrialized, Rich, Democratic (WEIRD) societies, and significantly less research on the human–dog bond has been conducted in other than WEIRD societies [7]. 

The need for culturally relevant research on the human–dog bond that focuses on when, how, why, and where, feelings, interests, and experiences are shared between humans and dogs, comes at a time when concepts traditionally eschewed in anthropological, psychological, and biological approaches to animal behavior, such as feelings (i.e., felt emotions [9]) and sentience [10], are increasingly seen as valid and important concepts to pursue in the study of how animals make their way through life. Theoretically and practically, the conceptual interplay between classical behaviorism (classical and operant conditioning) and classical ethology (instinct; fixed action patterns) that portrayed other animals mostly as automata, has largely run its course, clearing the way for newly realigned perspectives on emotion [11,12] and sentience [9,13] as important drivers of animal behavior. These developments in animal behavior science map onto contemporary perspectives on animal welfare (e.g., The Universal Declaration on Animal Welfare 2000–2014), and they inspired our review.

#### 1.2.2. Function and the Human–Dog Bond

For thousands of years, humans have selectively bred dogs for services to be rendered to them, including hunting, guarding, tracking, and herding for humans. Starting about 150 years ago, selective breeding of dogs became formalized through the foundation of breed associations and national kennel clubs, including the American Kennel Club (AKC) and similar kennel clubs in WEIRD societies organized under the umbrella of the Federation Cynologique Internationale (FCI). Modern representatives of the more than 200 recognized dog breeds commonly carry pedigrees attesting to multiple generations of breeding according to an accepted breed standard that outlines a breed’s desired form and function. Humans also train and breed dogs of differing ancestry to assist them with modern day life issues and to provide them with services and emotional comfort in times of need. 

While most current dog breeds were created and selectively bred with a specific function in mind, there is mixed evidence about whether and how behaviors associated with function are genetically encoded and preserved in current breeding populations [14,15]. Moreover, in WEIRD societies, often only a minority of fanciers of a given dog breed still breed, train, and care for their dogs guided by adequate knowledge of—and dedication to—the original function of the breed. 

All too often when it comes to acquiring and raising a pup in WEIRD societies, preferences for ‘looks’ trump informed knowledge of the function and character of the breed, or, in case of a mixed-breed animal, relevant information about the behavior of parents or family history of the pup [16,17]. Such uninformed acquisition of a canine companion can result in a mismatch between owner characteristics and owner expectations and a dog’s developing temperament and behavior. As the stories of Sulky and Cricket at the beginning of this introduction suggest, a mismatch between owner characteristics and owner expectations of the dog can negatively affect the human–dog bond and a dog’s welfare. 

Owner characteristics and expectations and a dog’s inherent or desired behavioral function do not only correlate with the quality of the human–dog bond and dog welfare in WEIRD societies, but also in non-WEIRD societies. A recent comprehensive review of ethnographic studies conducted in 124 globally distributed societies showed that human–dog bonds were closer, and positive care of the dogs increased, in the case of herding dogs, while the opposite was found for hunting dogs [7]. Form and function of the dog can thus be hypothesized to be important variables in the study of dog welfare in the context of the human–dog bond. 

### 1.3. Dog Welfare Defined

Psychologist Alexandra Horowitz suggests that while keeping pets in the home can be seen as reflective of an interest in animals, it is worth remembering that this is a model of animal captivity that also produces millions of homeless or unmanageable animals who are killed annually in the United States alone [18]. Focusing on dogs as pets, and while commenting on the field of human–animal interaction (HAI) research, Horowitz comments that in HAI studies, the dog is usually the silent partner, with little or no attention to the dog in and of itself, or to its welfare (Ibid.). In part inspired by the work of Horowitz and others, including the work of the evolutionary biologist and canine expert Marc Bekoff [19], we set out to explore to what extent and in which ways dog welfare (a dog’s state of being well) is considered in research on the human–dog bond. From the opening ‘tale of two dogs’ throughout the subsequent sections of this introduction, various aspects of dog welfare have come to the fore. However, for this scoping review, we needed a more systematic and established approach to the welfare of animals, and we decided to let our work be guided by the approach developed over the past 25 years by the bioethicist David Mellor and his colleagues [20]. 

As detailed in Section 2.4.3., the ‘Five Domains Model’ of human–animal interactions in assessments of animal welfare developed by Mellor et al. [20] focuses on (1) nutrition, (2) physical environment, (3) health, (4) behavioral interactions, and (5) mental state. From the initial assessment of our selected literature (see Section 2.3), we decided that the first four domains of the model would adequately cover aspects of dog welfare addressed in our sample. As Mellor and colleagues explain, the model is a guide to the assessment of the positive and negative impacts of human behavior on animal welfare, including the behavior of such persons as companion animal owners, owners of sport/recreational animals, animal trainers, service animal handlers, hunters, researchers, veterinary care staff, and pound/shelter staff, who are all persons serving as “the human” in the human–dog bond, illustrating the good fit of Mellor et al.’s model for the purpose of this scoping review. 

### 1.4. Aims

The aim of this scoping review is to obtain insight into the extent to which dog welfare is considered in the research and literature on the human–dog bond. We were inspired to conduct this review in part by our work on a university course on the anthropology of peoples and their dogs, as well as by our own experiences and activities with dogs, which, for some of us, span many decades. From our earlier work, we had the impression that research on the human–dog bond has been on the rise during the past decade, and we wanted to test that by selecting the literature for our review from the period 2012–2022 We also included literature that was published up until the date of our initial search in May 2023. We hope that this review will inspire further research on dog welfare as an aspect of the human–dog bond. We also hope that this review will have some practical significance for the lives of pet dogs and working dogs of all kinds, and their human partners, as well as for the lives of service and assistance dogs and their human beneficiaries. 

## 2. Materials and Methods

Given that the research on the human–dog relationship includes work in multiple disciplines, we decided on using the Scopus^®^ database (Elsevier B.V., Amsterdam, The Netherlands), which is the largest database of peer-reviewed literature and covers nearly 36,377 titles from approximately 11,678 publishers, of which 34,346 are peer-reviewed journals in top-level subject fields: life sciences, social sciences, physical sciences, and health sciences. We utilized the PRISMA checklist for scoping reviews [21]. As registration is not deemed necessary for scoping reviews, the review was not pre-registered.

### 2.1. Specifying the Research Question

The research question for this scoping review developed from what we see as a need for a greater emphasis on dogs in research on the human–dog bond [22]. Even research that proclaims to take a ‘dogcentric’ perspective often focuses on the impact of dogs on the lives of humans and not the other way around. For our purpose, a ‘dogcentric’ approach starts with the dog and focuses on what the dog contributes to the human–dog bond by means of the dog’s species-specific and domesticated nature, and on how interacting with humans affects the dog’s life. We predicted that there is a growing segment within research on humans and their dogs that focuses on dog welfare, and we wanted to know how much of this trend is reflected in research on the human–dog bond. Our research question for this scoping review is therefore stated as, ‘To what extent and in what ways is dog welfare addressed in the research literature on the human–dog bond?’. 

### 2.2. Identifying Relevant Literature

**Figure 1:**
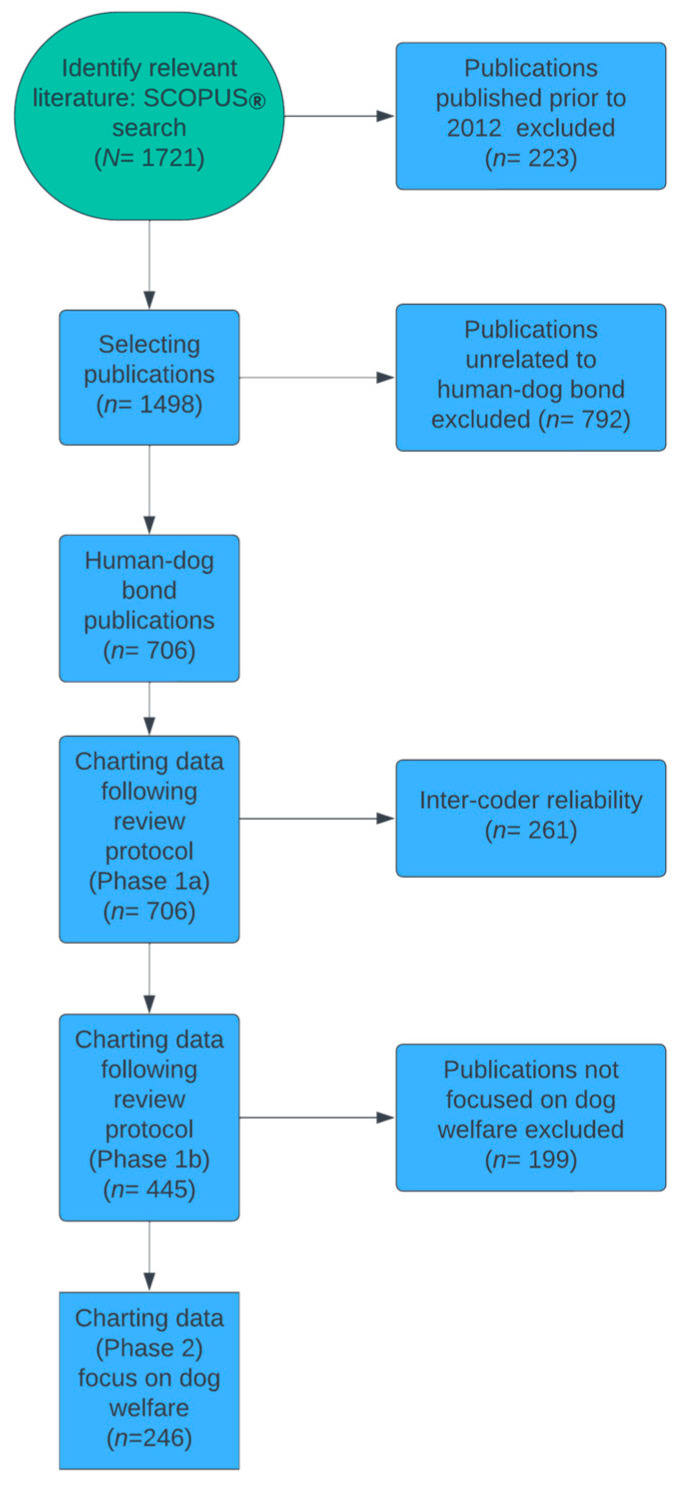
Selection of sources of evidence.

As illustrated in Figure 1, we developed a search strategy to identify literature relevant to answering the research question. To cast a wide net, the following three search terms were used, separated by the Boolean operator ‘AND’: human, dog, bond. On 29 May 2023, the first author used this strategy to search the abstract and citation database Scopus^®^. The search was limited to publications in the English language.

The initial search resulted in 1721 records, with publication dates spanning the period of 1939–2023. The search was downloaded and transformed into an MS Excel file (Version 16.84), and the 1721 publications identified by the search were downloaded from the publisher site or requested through inter-library loan. 

From earlier work, we had noted that the literature on the human–dog bond appeared to have significantly increased in volume over the past decade [23], and to test this, we selected records published during the years 2012–2022, and added the publications published in 2023 up to the date of the search. This resulted in 1498 publications. 

### 2.3. Selecting Publications

Next, we inspected abstracts of the 1498 preselected publications and eliminated those not related to the human–dog bond (e.g., records on strictly veterinary issues; chemical bonds, etc.). This data cleaning resulted in 706 publications, of which 680 were published during the years 2012–2022, and 26 in 2023 up to the date of our Scopus^®^ database search. Of the 706 selected publications, 689 are journal articles and 17 are book chapters.

### 2.4. Charting the Data—Phase 1a

We developed a protocol to chart the data relevant to our research question. We prepared a data extraction form in Microsoft Excel to include each of the 706 publications selected from the original Scopus^®^ master file that were charted following our protocol. Each article was read in its entirety and re-read if needed for clarity and proper understanding. We scored presence (1) or absence (0) for each level of each of the 3 criteria listed in the protocol. 

Protocol criterion 1: Consideration of dog welfare

(a) Primary focus of research/discussion on dog welfare.
(b) Secondary/tertiary focus of research/discussion on dog welfare.
(c) Dog welfare mentioned but not a focus of the research/discussion.
(d) Dog welfare not mentioned in publication.

We coded a primary focus on dog welfare in case the research or possible application thereof was centered entirely on (the) dogs’ state of being well (cf. 1.3). We coded a secondary/tertiary focus on dog welfare in cases where the interest or benefits for the human in the human–dog bond came first, while the welfare of the dog was also seriously or actively considered, for example, as in the case of ONE-HEALTH articles. We discussed articles that proved difficult for one of us to classify until we reached a consensus for how to code it.

We also scored (0 or 1) whether Institutional Animal Care and Use Committee (IACUC) approval of the research was mentioned in the publication, and whether the publication either referred to—or was specifically focused on—ONE-HEALTH. 

Protocol criterion 2: Dogs studied/discussed

(a) Pet dogs
(b) Working dogs
(c) Assistance/Service dogs

Protocol criterion 3: Human partner

(a) Owner (handler) [non-assistance/service dog]
(b) Professional (e.g., K9 police officer; researcher, etc.)
(c) Beneficiary (assistance/service dog)

#### 2.4.1. Inter-Coder Reliability

Charting of the 706 publications selected for our scoping review was performed by the first three authors. To assess inter-coder reliability upfront, the first three authors each worked on the same randomly selected subset [*n* = 261 (36.8%)] of the 706 publications selected for charting. Full agreement on the three protocol criteria (see above) was shown for 205 of the selected publications (78.5%; Fleiss’ kappa = 0.571). The 56 records for which there was disagreement were discussed and updated after consensus was reached. 

#### 2.4.2. Charting the Data—Phase 1b

The task of charting the remaining 445 publications was approximately equally divided among the first three authors. Several meetings were held during this phase to review and discuss any issues that arose. Following completion of this task, the first author verified the coding of the 3 protocol criteria for each of the 445 publications, and any remaining issues were discussed and resolved with the respective coder prior to releasing the full dataset of 706 records for the second phase of data charting.

#### 2.4.3. Charting the Data—Phase 2

For each of the 706 publications on the human–dog bond, the first author identified the country and greater geographical region of origin based on the country of residence of the main author of the publication (as determined by institutional affiliation). The following geographical regions were defined: Africa (publications from 5 countries), Asia (11 countries), Europe (23 countries), Middle East (1 country), North America (2 countries), Oceania (2 countries), and South America (5 countries). 

Next, each of the 246 publications charted as having either a primary or secondary focus on dog welfare were inspected and scored by dog welfare domain, based on the 2020 five domains model developed by David Mellor and colleagues (Mellor et al. 2020 [20]). As mentioned previously, these welfare domains include Nutrition (nutritional conditions and their associated effects), Environment (physical environmental conditions and their associated effects), Health (health conditions and their associated effects), Behavior (behavioral interactions and their associated effects), to which was added the domain Other for any other aspect of welfare not specified in the first four domains adapted from Mellor et al.

Finally, for each of the 706 publications, the scientific domain best describing the research/discussion was determined and scored by the first author as one of the following: (Animal) Behavior, (Animal) Welfare, Medical Science, Social Science, and Veterinary Science. The determination by scientific domain was based on journal/article title as well as by the publication’s main author’s position and/or affiliation. 

### 2.5. Collating, Summarizing, and Reporting the Data 

In the results sections that follow, we first report on summaries derived from the full data set of 706 publications on the human–dog bond. We follow this with summaries and analyses derived from the publications on the human–dog bond with either a primary or secondary focus on dog welfare (*n* = 246). We conclude with a more detailed discussion of publications with a focus on dog welfare that represent the behavioral interaction domain (Behavior) of the five-domain model of animal welfare, i.e., behavioral interactions and their associated effects (*n* = 114). These latter publications are attached to this paper within the reference list, and the remaining publications charted in this scoping review can be accessed as Supplementary Material.

## 3. Results

### 3.1. Consideration of Dog Welfare in Human–Dog Bond Research Publications

As we predicted, and as illustrated in Table 1, our summative analysis showed that the relative number of publications on the human–dog bond increased notably from an average of 40.2 (SD = 27.9) per year during the years 2012–2017 to an average of 87.8 (SD = 26.2) per year during the years 2018–2022. 

During the years 2012–2017, an average of 11.8% of the publications on the human–dog bond featured a primary focus on dog welfare, while for the following period 2018–2022, this percentage was 24.2%, which is also a notable increase. Publications with a secondary focus on dog welfare accounted for 10.2% during 2012–2017 and 14.2% during 2018–2022, mirroring, albeit less substantially so, the upward trend shown for publications with a primary focus on dog welfare. The records for the first months of 2023 suggest a continuation of the rise in human–dog bond publications with a primary focus on dog welfare.

Taken together, over the period 2012–2022, 34.8% of publications on the human–dog bond had a focus on dog welfare, with 20.2% of the 680 publications at least mentioning dog welfare. Next, we compared how the focus on dog welfare was distributed among publications on the types of dogs that we defined for our review: pet dogs, working dogs and service/assistance dogs.

#### 3.1.1. Consideration of Dog Welfare by Category of Dog

As shown in Table 2, pet dogs were by far the most studied type of dog, representing 80.7% of the 706 publications on the human–dog bond, compared to working dogs, 3.4% of the total, and service dogs, 12.3% of the total. Mixed samples, mostly made up of pet dogs and service dogs, accounted for 3.5% of the total.

Looking within each type of dog, publications with a primary focus on dog welfare accounted for 22.6% of all publications on pet dogs, with 16.6% having a secondary focus on dog welfare, and 18.4% mentioning—and 42.3% not mentioning—dog welfare. The percentages for working dogs, presented here in the same order as for pet dogs, were 25.0%, 12.5%, 16.7%, and 45.8%, respectively, and for service dogs, 16.0%, 0%, 24.0%, and 60.0%. 

IACUC compliance was reported in 66 research publications, of which 19.7% had a primary focus and 12.2% a secondary focus on dog welfare, with 9.1% mentioning, and 59.0% not mentioning dog welfare.

Our next analysis looked at the country and geographical region of origin of the 706 publications on the human–dog bond, and we noted the focus or lack thereof on dog welfare for each country and region.

#### 3.1.2. Consideration of Dog Welfare by Country and Geographical Region

Our analysis showed that Europe topped the number of publications on the human–dog bond with 301 publications (42.6% of total), followed by North America, 277 (39.2%), Oceania, 52 (7.4%), Asia, 37 (5.2%), South America, 30 (4.2%), Africa, 6 (0.8%), and the Middle East, 3 (0.4%). 

As detailed in Table 3, for regions with 10 or more human–dog bond publications, Europe showed the highest percentage of publications with a primary focus on dog welfare, 24.3%, compared to North America, 18.4%, Asia, 16.2%, Oceania, 13.5%, and South America, 13.3%. Taken together, the findings of this geographical analysis show that the human–dog bond is a topic of scientific inquiry across the major regions of the world, with relatively high numbers of publications concentrated in Europe and the Americas.

#### 3.1.3. Top Journals Publishing Articles on the Human–Dog Bond with a Focus on Dog Welfare

As shown in Table 4, the journal *Animals* stands out as the leading journal in our selection of journals with three or more articles on the human–dog bond with a focus on dog welfare, publishing 14.3% of the 108 articles. Eight (42.1%) of the 19 journals in this selection are from veterinary science, and together, they represent 47.2% of the 108 articles with a primary focus on dog welfare and 41.7% of the 60 articles with a secondary focus on dog welfare, illustrating the importance of veterinary science for the study of welfare as an aspect of the human–dog bond. 

#### 3.1.4. Focus on Dog Welfare by Scientific Domain

As mentioned under Section 2.4.3, the scientific domain for each of the 706 publications on the human–dog bond was categorized as either (Animal) Behavior, (Animal) Welfare, Medical Science, Social Science, or Veterinary Science, with Behavior broadly defined and including work on animal cognition. As shown in Table 5, the greatest number of publications on the human–dog bond concerned work on Behavior (26.3% of the total), closely followed by Social Science (24.4%), Medical Science (23.2%), and Veterinary Science (22.7%). Publications from the domain of Welfare accounted for 3.4% of the 706 publications on the human–dog bond.

Underscoring the significance of Veterinary Science for the study of dog welfare in the context of the human–dog bond, 61.9% of the publications in Veterinary Science had either a primary or secondary focus on dog welfare, compared to 58.3% in Welfare, 48.3% in Behavior, 22.7% in Social Science, and 22.0% in Medical Science (Table 5). 

### 3.2. Aspects of Dog Welfare

Following up on our analyses on the prevalence and origin of work on dog welfare in the context of the human–dog bond, we investigated the distribution of specific aspects of dog welfare among publications addressing the human–dog bond.

#### 3.2.1. Aspects of Dog Welfare by Category of Dog

Behavioral interactions and their associated effects (Behavior; see Section 1.3 and Section 2.4.3) were the most common welfare domain addressed in publications on the human–dog bond with pet dogs as subjects (46.0% of 224), followed by Health (36.6%) [Table 6]. Studies on working dogs mirrored this pattern, with 55.6% of the publications dealing with Behavior, and 22.2% dealing with Health. Studies on service dogs showed the reverse of this pattern: Behavior 33.3% and Health 55.6%. However, considering the small sample size of publications on working dogs and service dogs, it is hard to draw conclusions from these findings.

#### 3.2.2. Aspects of Dog Welfare by Scientific Domain

As shown in Table 7, behavioral issues (Behavior) were the most often addressed dog welfare issues in publications from animal behavior science (*Behavior*; 67.2% of 58), as well as from animal welfare science and social science (*Welfare*, 78.6%; *Social*, 64.1%). Unsurprisingly, health issues (Health) ranked high in medical science and veterinary science publications (*Medical* 75.0%; *Veterinary* 47.5%), while behavioral welfare issues (Behavior) also featured prominently in publications from veterinary science, (*Veterinary* 34.3%), showing the diverse approach in veterinary science to dog welfare in the context of the human–dog bond.

#### 3.2.3. Behavioral Aspects of Dog Welfare by Scientific Domain

The analysis presented in Section 3.2.2. showed that behavioral interactions and their associated effects on dog welfare (i.e., welfare domain Behavior) were the most common aspect of dog welfare addressed in our selection of publications on the human–dog bond. We followed up on this finding with a detailed look at the various types of behavioral interaction issues and we identified the eighteen distinct ones listed in Table 8a.

With 29.8% of the total, owner characteristics and their interactional effects on dog welfare were the most often addressed behavioral dog welfare issue across scientific domains (four out of five scientific domains reporting), followed by problem behaviors (14.0%; three of five scientific domains). Dog training was another relatively common behavioral dog welfare issue (9.5%; five of five scientific domains), followed by interactional effects related to attachment to owner (7.9%; two of five scientific domains), dog characteristics (7.0%; four of five scientific domains), and dog emotion (7.0%; three of five scientific domains). Intraspecies interactions (5.3%) and veterinary visits (5.3) were specific to scientific domains with, respectively, one and two scientific domains reporting on these behavioral interactional dog welfare issues. Table 8b shows the sources of evidence for the above analysis.

## 4. Discussion

### 4.1. The Five-Domain Model of Dog Welfare and the Sentient Dog

As the Behavior domain was the most frequently addressed welfare domain in the selected literature of this scoping review with a focus on dog welfare, we proceeded with a more detailed review of the publications in this area. The behavioral interaction domain of Mellor et al.’s five-domain model of animal welfare (Behavior) breaks down positive human attributes and behaviors into attitude, voice, aptitude, and handling/controlling. For attitude, confidence, caring, sensitivity, patience, kindness, and empathy are listed as positive influences on the human–animal interaction, resulting in a bonded, alert, and responsive animal ready to explore novel events. A calm, clear, and encouraging human voice adds to that positive mix, and so does an experienced and skilled aptitude on the part of the human partner. Handling and controlling of the animal by the human partner should be skillful and gentle, based on an insightful mix of firmness and restraint, and a focus on rewards. Deficiencies on the part of the human in those attributes and behaviors can result in the animal being anxious and insecure in the interaction with the human and can trigger fear and even panic in the animal, as well as helplessness and an avoidance of novel events [20]. In terms of the operational definition of the human–dog bond that we advance in this paper, we propose that with the right attributes and behaviors on the part of the human, the likelihood that the dog will be ready to share feelings, interests, and experiences with the human partner (see Section 1.2.1) and establish a bond will be greatly increased.

Groetzinger Strickler [101] states that in recent years, the relationship between pet and owner has changed significantly. Mirroring empirical and theoretical developments in the study of animal behavior as a whole [9,11] (see also Section 1.2.1), professionals including veterinarians and trainers, as well as owners, are more likely than before to perceive companion animals as thinking, feeling beings. According to Groetzinger Strickler, this allows for a transition in training and care from one of poor and inadequate behavioral welfare to an approach that acts on the full potential of the animal. Our own perspective here acknowledges the science, which shows that many animals that were previously depicted as mere stimulus response automata are indeed thinking and feeling beings, capable of making their own decisions while navigating through life. We think that this new understanding of animal behavior both maps onto and enriches the five-domain model of animal welfare, especially as it applies, but is not limited to, the human–dog bond.

#### 4.1.1. The Pre-Acquisition Phase and Dog Welfare

Many of the articles that we reviewed for this presentation discuss or investigate behavioral elements of Mellor et al.’s five-domain model. Several authors stress that the foundation for a close bond between a human and a dog starts at the time the dog enters the human’s life, or even before that, during the phase the human contemplates bringing a dog into her life. Holland and colleagues [16,75] warn against impulse acquisitions without any research into the background of the dog or its breed. These authors state that acquiring a dog out of a desire to help a vulnerable animal, including adopting a dog from a shelter, does not necessarily guarantee the establishment of a bond favorable to the welfare of the dog, unless and until the human contributes the right mix of attitudes and behaviors expected to positively affect the dog’s welfare.

Research by Bouma et al. [134] shows that people who frequently read books about owning dogs and who often talked about this with others were more likely to make an informed decision about getting a dog, compared to people who visited websites offering or selling dogs and as such, were more prone to impulse buying. Pirrone et al. [97] stress that potential owners should see the puppy they intend to buy while it is with its mother, as this can provide useful information on the behavior of the mother and can help to predict the behavior of the puppy as an adult. Diverio et al. [103] show that there can be a gap between what people imagine as the ideal dog and their actual dog, and that adequate education of potential dog owners about the specifics of the breed and background of the dog, and of the positive effects of training activities on the dog’s behavior, is important for establishing a bond that optimizes dog welfare.

Herron at al [104] point out that newly adopted shelter dogs often experience separation anxiety, and that pre-adoption counseling can be helpful to inform the new owner about effective prevention tools to use in the home to minimize the development of separation anxiety. Reese [80] suggests, for shelter dog adoptions to be successful, matching discussions with a potential owner before showing any dogs are important, as they can serve as a guide to show only those dogs that in terms of temperament and behavior can be predicted to be a good match with the new owner. Taken together, these studies show the responsibilities of potential and new dog owners for doing their part in laying the foundation from which a bond that optimizes dog welfare can develop.

#### 4.1.2. Owner Characteristics and Dog Welfare

The articles we reviewed for this section present a variety of approaches to the role of owner characteristics in the human–dog bond and dog welfare. Different aspects of the Behavior domain of dog welfare are implied, associated with different contexts and perspectives. Brubaker and Udell [74] found inspiration in human developmental science for their approach to the human–dog bond. Developmental science shows a link between parenting style (and parenting practice) and social developmental outcomes in children and adolescents [135]. In some sociocultural groups, especially in WEIRD societies, authoritative parenting, which gives children some input in the interaction and respects their relative autonomy, tends to result in positive social developmental outcomes, such as positive peer relations and self-confidence and self-esteem in social situations. Authoritarian parenting, based on less or no child input in the interaction, and, especially, rejecting/neglecting parenting, have been associated with less positive social developmental outcomes (Ibid.). Brubacker and Udell showed that authoritative owners with high expectations and high responsiveness tended to have dogs that were highly social and sensitive to social context and good at solving an experimental problem task. In contrast, dogs with authoritarian owners (high expectations and low responsiveness) tended to show less positive social behavior and problem-solving skills. These authors suggest that a highly social and cognitively well-functioning dog can be seen as a “happy” dog, and that their research shows that the style in which owners interact with their dog can have a direct effect on the dog’s welfare. 

González-Ramírez [78] showed that compatibility in energy levels, temperament, and daily activity between owners and their dogs was associated with less aggressive and fearful behaviors and higher trainability scores in dogs, compared to less compatible human–dog dyads. Powell [118] found that owner conscientiousness, extraversion and openness, and the quality of the bond with their dog (defined here as “attachment”), were positive factors in the dog’s response to clinical behavioral intervention. Stevens et al. [76] provide evidence that owners who scored higher on cognitive measures were more likely to have their dogs complete an obedience training program. Karvinen and Rhodes [85] showed that owners who train their dog in agility engage in more physical activity with their dogs but less without their dog, compared to other dog owners. As physical activity is generally beneficial for a healthy dog, engaging in agility can indirectly have a positive effect on dog welfare.

We detected a growing interest in personality profiles and mental health of owners and other humans in the close social circle of a dog and the possible links to the quality of the human–dog bond and dog welfare. Dodman et al. [89] found a significant correlation between moderate depression in male owners and the use of aversive and confrontational dog training techniques. Tiplady et al. [105] report on the effects of domestic violence on dog welfare. Dogs that were the target of redirected violence were more likely to be owned by women rather than men, children, or both partners. These authors also found that people experiencing domestic violence are often unwilling to confide in veterinarians or seek help from animal shelters. Hall [91] studied dogs in families with children with neuro-developmental disorders and with neurotypical children. She found that harsh contact and rough and tumble play with children with neuro-developmental disorders, and having to cope with child meltdowns and tantrums, were negative factors in terms of the welfare of dogs in such families. Hall suggests a safe haven for the dog to escape to, parent’s awareness of stress signs, and child education in dog interaction to help limit negative effects on dog welfare.

Ferrell and Crowley [83] studied emotional support dogs’ interactions with their human beneficiaries and report that approximately one in seven ESA dogs in their study may not have been receiving consistent quality care. Moreover, explicitly discussing animal welfare was not associated with actual welfare items except for adequate shelter. While cautioning that their findings are preliminary, these authors suggest that oversight of ESAs may need to include an assessment of caretaking behaviors and a determination of how to best meet the needs of both the beneficiary and the ESA dog.

Giraudet [84] suggests that dogs may show greater levels of stress in the presence of children, and that the welfare of assistance and therapy dogs who may interact with children remains underexplored. She suggests that for children, the benefits of interacting with dogs may outweigh the risks, but that this is not necessarily the case for dogs. Older children and adolescents may have interactions with dogs that have a more positive effect on dog welfare. Bathurst and Lunghofer [92], for example, report on ‘Lifetime Bonds’, a program in Chicago that teams up at-risk youth, particularly adolescent males, and at-risk dogs impounded as victims of cruelty and neglect. Both youths and dogs help each other in this program, with the youths learning about the responsibilities of taking care of another sentient being, and the dogs having a chance to overcome some of the effects of their violent past through the care and training provided by their young partners.

Behavior problems of dogs are an important dog welfare concern, as they are one of the most common causes of relinquishment to shelters and a common reason for euthanasia [136]. Anxious dogs can be more vulnerable to disease and, through aggressive behavior, can become a public health concern (Ibid.). Several authors reviewed here discuss the role of owner characteristics in dog behavior problems and ways to increase owner knowledge about the causes and interventions associated with problem behavior. Philpotts [77] argues that while improving an owner’s knowledge through an education intervention has the potential to improve dog welfare, the complexity of dog welfare and dog ownership requires significantly more informed input from cross-disciplinary and boundary-crossing research on dog welfare in the design and execution of such education intervention. Westgarth et al. [87] express a similar view while discussing responsible dog ownership and ways to promote it. These authors argue that telling owners that they should be responsible is of limited use for promoting behavior change if it is not accompanied by targeted education on the dog’s role within the family and wider society.

McGreevy et al. [102] argue for an applied science of dogmanship. They emphasize the role veterinary behavioral medicine can play in such a science, using information technology tools such as a computer or personal electronic device-based interactive doglogbook and dogmanship coaching tool, based on a detailed dog–human interaction ethogram. They suggest these tools can be combined with real-time measures of heart rate, balance and movement to give biofeedback, as the user develops timing, consistency, and calmness, toward an ideal interaction pattern that protects and enhances dog welfare. In a similar vein, Alcaidinho et al. [94] report on a pilot study on whether the use of a specially designed smartphone application registering owner and dog interaction can increase the perceived strength of the bond between owners and newly adopted dogs from a California shelter, with the goal of reducing returns to the shelter. And in the United Kingdom, a smartphone application that rewards owners for walking their dog and taking care of its wellbeing with points that can be swapped for vouchers, gained 50,000 registered users since its launch in March 2022 [95].

#### 4.1.3. Problem Behaviors as a Dog Welfare Issue

Problem behavior is discussed extensively in the literature on the human–dog bond reviewed here, and it is often linked to the welfare of both dogs and their human partners [122]. There is no one-size-fits-all definition for problem behavior, as what is perceived as problematic dog behavior for one dog owner may not be a problem for another. We found some reoccurring themes in the literature, however, including aggressive behavior and separation anxiety. This latter term, borrowed from human psychology, refers to often destructive behavior exhibited by a dog when left at home with the owner away and no other humans present. Several authors emphasize that problem behavior can be linked to the specific functioning, or malfunctioning, of a given human–dog bond. This implies that in terms of remedying the problem behavior, the behavior and characteristics of both the human and the dog need to be considered. The first author has experience with this reality, going back many years when he worked as a professional dog trainer in a large boarding and breeding facility in Germany. My (PV) task was to correct problem behavior in dogs boarded at the facility where I worked, ranging from serious issues such as aggression toward other dogs or people, to more mundane issues, such as excessive leash pulling when walked, or ignoring commands and generally not being a good canine citizen in the opinion of the owner. I would work intensively with such dogs for about 6 weeks, at the end of which I would demonstrate the behavior of the dog to its owner in the expectation that the dog’s behavior was now acceptable to the owner. In some cases, this was not the end of it, however, as the dog would be returned to us by the owner with the same problem behavior occurring again. This is when I learned that to remedy problem behavior in dogs, both the owner and the dog need to be trained.

Veterinary behavioral scientist Daniel Mills and colleagues [114] propose eight overlapping dimensions to be characteristic to the human–dog bond: content of interactions, diversity of interactions, reciprocity versus complementarity of interactions, quality of interactions, frequency and patterning of interactions, intimacy, cognitive perspective of interactions, and multidimensional qualities. They link these eight dimensions to (1) the emotional involvement/dependency, (2) common interest, and (3) working partnership that make up a human–dog bond. We see significant similarity here to our own view of the human–dog bond as one of shared feelings, interests, and experiences. From this conceptual approach to the human–dog bond, Mills and colleagues suggest that approaches to problem behavior in dogs should consider the bidirectionality of the human–dog bond, including the expectations and biases that both partners may bring to the bond. This, of course, is a more eloquent way of stating what the first author learned as a professional dog trainer all those years ago: it usually takes two, the owner and the dog, to remedy dog problem behavior.

Stephen-Lewis et al. [115] make a similar case for the need to consider both owner and dog when dog problem behavior occurs in their discussion of reactivity in dogs, which may be a problem to some owners and less of a problem to others. They propose that dog reactivity is influenced by canine characteristics, human expectations, and human capabilities, each of which features multiple subfactors, such as culture and lifestyle. Canejo-Teixeira [124] distinguishes between functional and dysfunctional dyads in the context of dog aggression and other problem behavior. Human members of functional human–dog dyads are described as being responsible for the dog’s welfare, providing the necessary care, and avoiding situations of risk. Humans in dysfunctional dyads are described as showing the opposite characteristics, and as not always being aware that their behavior may be placing themselves and/or their dog at risk. Taken together, each of these authors underlines the need to consider the bidirectionality and overall functioning of the human–dog bond when dog problem behavior occurs.

A study by Dinwoodie and colleagues [123] provides a good insight into various types of problem behavior in a large sample survey of 4114 dogs of mixed and pure breeds submitted by 2480 dog owners. Male and female, mostly neutered, dogs were equally represented in the sample. Problem behavior was reported in 85% of the survey responses and included anxiety and fear, aggression, excessive barking, house soiling, destructive behavior, and a range of other issues. The survey focused on dog characteristics and found that age, neutered status, origin, and lineage were all correlated with problem behavior. Rajapaksha [125] reflects on age as a dog characteristic in problem behavior and suggests that problem behavior is more difficult to diagnose in older dogs than in younger dogs. The reason for this is that many degenerative disease conditions in older dogs are reflected as a change in behavior. Rajapaksha recommends detailed clinical examinations for older dogs and owner education and behavioral enrichment as measures to improve the welfare of older dogs.

While framing dog characteristics in the human–dog bond context, research by Dubé [116] showed that problem behavior was more common in dogs described as highly sensitive and when there was a mismatch between owner personality and dog “personality”. Gates et al. [111] report on problem behavior in dogs adopted from shelters. In their sample, most dogs were reported to exhibit problem behavior, including aggression toward people or dogs, destructive behavior in the home, and excessively high energy. Interestingly, most new owners showed little concern about the problem behavior, but these authors do recommend support programs for adopters to increase adoption satisfaction.

While not apparent in the Gates et al. study, problem behavior can result in adopted dogs being returned to a shelter, or to dogs being relinquished to a shelter in the first place. Powel and colleagues [118] suggest that problem behavior is in fact one of the leading causes of dog relinquishment. They surveyed owner perception of problem behavior of relinquishing and non-relinquishing owners to see whether there were differences between these two types of owners in how problem behavior was perceived. Relinquishing owners were found to be significantly less likely to report problem behavior compared to the matched sample of non-relinquishing owners. Powel et al. suggest that if the relinquishing owners were indeed answering honestly, they might have been less informed about normative dog behavior and did not always recognize their dog’s behavior as a problem.

Several authors in our review discuss separation anxiety, including in dogs newly adopted from shelters, and possible interventions for this problem behavior. One strategy that has been suggested is for owners to show little or no excitement during arrivals and departures from the home. Recent research by Teixiera and Hall [120], however, showed that this strategy had no effect in mitigating separation anxiety in newly adopted dogs. Feuerbacher and Muir [110] tried to use the return of the owner as a reward for teaching a desired behavior to dogs, and the ability to stay alone without showing signs of separation anxiety increased over baseline, but none of the dogs in the study was able to stay alone for very long.

#### 4.1.4. Dog Training as a Dog Welfare Issue

Dog training is related to dog welfare in multiple ways, including through the goals of the training as well as through its execution. The literature on dog training that we identified as part of our search for articles on the human–dog bond and dog welfare deals exclusively with dog training in WEIRD societies, highlighting the need for comparative and ethnographic work on dog training and dog welfare from other parts of the world. Training goals can be indirectly related to dog welfare, as illustrated by d’Angelo et al. [56], who report on a training program in Italy with the goal of increasing the chances of shelter dogs being adopted. The program is described as having met this goal, as adult and older dogs that underwent a 4-month good canine citizen training program were more likely to be adopted than an age-matched sample of untrained shelter dogs. In case the adoptions of trained shelter dogs indeed resulted in a new “forever home” for these dogs, it can be argued that their welfare was indirectly served by the training program.

Vitulli et al. [59] report on another shelter dog training program in Italy that is guided by a systematic pre-training assessment of the dogs to be included in the training program. Commenting that shelters are often lacking the financial and staff resources to manage training programs, the authors propose students as a source of labor for such programs. In Italy, secondary education students can take anthrozoology courses that are focused on dog training methods respectful of dogs and the human–dog bond. As the mission of many of these courses is to complement in-class learning with a practicum, Vitulli et al. see this as an opportunity for shelters to team up with schools to have students conduct their practical learning through participation in a shelter dog training program.

The Washington Humane Society (WHS) teamed up with the Walter Reed National Military Medical Center (WRNMMC) in a shelter dog training program. Alers and Simpson [60] report on this program, in which soldiers recovering at WRNMMC learned and applied positive reinforcement training to dogs waiting for adoption at WHS. Similarly, as in the program discussed by Vitulli et al. [59], the dogs in the training program were prescreened on health and behavior. Both dogs and soldiers were said to benefit from the program, as it increased the chances for the dogs to be adopted, and the soldiers developed new skills, built positive bonds with the dogs, and continued to serve their community.

The reference to “positive reinforcement” in the above section brings us to several articles from our selection here that we consider to be especially relevant and thought provoking in terms of the relationship between dog welfare and training. In a published research proposal focused on the training of working dogs, Vieira de Castro et al. [57] present an overview of traditional dog training methods based on the behaviorist principles of classical conditioning and operant conditioning. Classical conditioning methods include a conditioned punisher such as when an initially neutral stimulus (e.g., the word ‘No!’) is paired with a punishing stimulus, e.g., a slap. After repeated pairing of these two stimuli, the word No! can stand alone in achieving the effect of stopping any undesired behavior the dog may be engaged in. The second classical conditioning method is referred to as a conditioned reinforcer, where a previously neutral stimulus, e.g., a clicker, is repeatedly paired with a reward, e.g., a food reward. After repeated pairing of these two stimuli, the clicker can stand alone in reinforcing a desired (to be trained) behavior that the dog is engaged in. Operant conditioning provides four training methods: positive punishment, negative reinforcement, positive reinforcement, and negative punishments. Positive punishment is used to try to stop an undesirable behavior, for example by jerking on the dog’s leash or by yelling at the dog. Negative reinforcement involves stopping a stimulus that is being perceived as unpleasant by the dog (e.g., the vibrations caused by an e-collar [65]) as soon as the dogs shifts to or shows a desired behavior. Positive reinforcement involves providing the dog with a reward, e.g., a dog treat, as soon as the dogs exbibits the desired behavior. Negative punishment refers to removing a stimulus perceived as pleasant by the dog after it shows an undesirable behavior, e.g., a time-out session in a dog crate.

Trainers engaged in these techniques often refer to purportedly genetically predisposed temperament traits in the dog, such as prey drive (shown, for example, by eagerness to chase down and retrieve a toy), or food drive, shown by interest in obtaining dog treats or other foods. In case of positive reinforcement, a toy or treat can be the reward, or can become a conditioned reinforcer. And in the case of conditioned punishment and positive punishment, specific behaviors of the handler can be the punishers. As with the pigeons in BF Skinner’s operant conditioning chambers, there seems to be no need for the dog to do much else than to focus on the incoming rewards or punishers and to behave accordingly as it is driven along by its drives.

Pręgowski [23] critically reviews traditional and more recent dog training techniques. He comments that dog training practices tend to rely either on the traditional approach of positioning the owner/handler as the dominant “leader of the pack”, resulting in a discipline heavy approach, or on behaviorism, with currently more of an emphasis on positive reinforcement than on punishment. Pręgowski comments that what is left out of the picture of this otherwise coherent and force-free positive reinforcement approach is the well-being of the trained dog, including the dog’s immanent needs, especially psychological needs, such as attention and bonding.

Smith et al. [61] used an ethnographic approach to study the training of police dogs in the United Kingdom. These authors suggested that in working dog training, the dogs tend to become “instruments of human work whose capabilities and subjectivities are left unexplored”. The central aim of their study was to obtain a better insight into the degree to which this assertion holds in police dog training, and whether and how this type of training creates a bond between dog and human. The police instructors in the study shared that they saw the bond between dog and handler as the central pillar in the training relationship. Through a mix of interviews and observations and the survey of video and photo materials, Smith and colleagues showed how interests, feelings, and experiences were shared in the training through “interactions between voices (praising, commanding, warning, types of barks and growls), sensing and touching bodies (hands, noses, teeth, fur, licks, and bites), material objects (toys, bite sleeves, leashes, ground, and kennel)”. The trainers commented that they thought that their canine partners had a better quality of life than the average pet dog resting at home, but with reference to the work of Bekoff [12], the authors state that this view omits the consideration of other aspects of a dog’s life, such as play, including with other dogs, and reproduction and care of offspring.

Finally, in this discussion of articles on dog training and dog welfare included in our scoping review, LaFollette et al. [64] report on a study that investigated the relationships among training methods, posttraumatic stress disorder (PTSD) severity, service dog behavior, and the veteran–service dog bond in a program matching military veterans with service dogs. The veteran used multiple training methods, and positive reinforcement or bond-based training methods were associated with reporting more positive outcomes, while positive punishment was associated with more negative outcomes. LaFollette and colleagues suggest that education about training methods could be beneficial for service dog efficacy and welfare.

#### 4.1.5. Attachment to Owner as a Dog Welfare Issue

Earlier in this scoping review, we discussed the adaptation of the concept of parenting style to the study of the human–dog bond and dog welfare. Inherent to this approach is the view that in the human–dog bond, the human is the “adult”, and the dog the immature, dependent, partner. Attachment theory is another approach from human developmental science that has been adapted to the study of the human–dog bond and dog welfare. We identified several attachment publications in our search, and we discuss them here. Before discussing individual publications, it is useful to review the history of attachment research. Inspired by the ethologist Konrad Lorenz’ work on imprinting in gosling, John Bowlby [137] developed attachment theory based on the premise that in species where infants are fully dependent on their parents for survival, including the human species, newborns are biologically predisposed to establish a close bond with their caregivers, thus promoting their chances of survival and, ultimately, reproduction at sexual maturity.

The psychologist Mary Ainsworth developed a standardized test titled ‘The Strange Situation’ (SST) for measuring the quality of attachment to the primary caregiver of human infants between 1 and 2 years of age. The SST is purported to identify a secure attachment pattern and distinct patterns of insecure attachment. Like parenting style/parenting practice theory and research in developmental science, attachment theory has been further developed through longitudinal research that has linked infant attachment patterns as measured with SST with later social developmental outcomes, including, in the case of secure attachment, positive peer relations in childhood and adolescence, and even positive romantic relationships in adulthood.

The SST standardized test of attachment in human infants has been adapted for use with the human–dog dyad to measure the attachment of dog to owner, and in some cases, owner to dog and dog to dog in cohabiting dog pairs. Several human–dog bond studies refer to the adapted SST by its original name, while other studies refer to it as ‘The Secure Base Test’ (SBT) [31,32]. Other studies have developed surveys to be completed by dog owners to assess attachment of the dog to the owner without employing the SST [26].

Carreiro and colleagues [25] studied the correlation between SST attachment and sleep patterns in mature dogs. They found that secure attachment in dogs was associated with more time spent in NREM sleep. They refer to a previous EEG study on pet dogs that showed that participation in a negative social interaction, including separation from the owner, was associated with a decrease in NREM duration following the negative interaction compared to following a positive social interaction. Commenting on these findings, Carreiro and colleagues suggest that securely attached dogs might have had a more stable inner state due to the secure base provided by the owner. Thielke and Udell [29] compared dog attachment to shelter and foster care staff with the dog’s performance on cognitive tasks. They report that secure attachments were associated with higher persistence in cognitive tasks in dogs, and with less survey-rated neurotic behavior, compared to insecurely attached dogs.

Saavedra-Aracena and colleagues [27] asked the question of whether attachment status would be associated with roaming behavior in owned but free-ranging dogs. The study was conducted at Navarino Island in Southern Chile, where 30% of owned dogs roam free. They found that owners of free-ranging dogs are less likely to represent a secure base to the dogs as measured through SST and complementary surveys. Following these findings the authors argue for the need for educational campaigns to foster responsible dog ownership. Sipple et al. [28] found comparable attachment patterns between dogs and their owners as in similar studies, but no evidence for attachment between cohabiting dog pairs. These authors suggest that bonds formed among adult dogs likely serve a different function than those between dogs and their owners. Konok and colleagues [33] found a correlation between insecure avoidant attachment in owners and separation anxiety in their dogs. They suggest that avoidant owners may not provide a secure base for their dog when needed, and as a result, the dog may develop separation anxiety.

Lewis [30] critiques the application of attachment theory and methods to the study of the human–dog bond and dog welfare. Lewis argues that this approach infantilizes mature animals and proposes instead that dogs form mature social bonds with their guardians and that separation anxiety is the result either of the frustration of mature adult group behaviors, or an overdependency fostered by the guardian. Lewis suggests that social bonds are adaptive, as they maximize survival and reproductive fitness and, through social buffering, can ameliorate the physiological response to acute stressors. Lewis adds that it is reasonable to infer that domestic dogs are emotionally and socially mature individuals with adult social skills specifically adapted to human social groups. Importantly, Lewis argues that separation anxiety can arise when a dog’s natural social behaviors are thwarted when left alone in the home, resulting in frustration of normal adult social behavior. Such frustration may also occur when dogs are left alone without prior rigorous exercise or other suitable sensory stimulation. In sum, such frustration of normal adult social behavior when left alone at home is fundamentally different from a subjective feeling of anxiety in the absence of an attachment figure. Lewis concludes that from the perspective of dog welfare, it is necessary that dogs are no longer viewed as immature, infantile individuals, but rather as mature sentient beings with a predisposition to bond with humans.

#### 4.1.6. Dog Characteristics as a Dog Welfare Issue

Like owner characteristics, dog characteristics affect the human–dog bond, but our review suggests that more needs to be learned about how this works. Samet and her colleagues noted that survey studies seeking to define the human–dog bond commonly do so without specific questions about the dog’s investment in the bond. Samet et al. define the human–dog bond as “the unique, dynamic and reciprocated relationship between a person and dog, one in which each member can influence the other’s psychological and physiological state”. As a first step to develop more dog-centered questions for survey research on the human–dog bond, Samet et al. conducted a series of semi-structured interviews asking dog owners and handlers to comment on their perception of a dog’s place in the human–dog bond. Themes that emerged included ‘adaptation’, ‘understanding of a dog’s preferences, likes, and dislikes’, and ‘affirmation’. Subthemes included ‘boundaries’ and ‘expectations’ (within adaptation), ‘excitement’, ‘proximity’, ‘affection’, and ‘recall’ (within affirmation) [38].

Protopopova and colleagues investigated to what extent behaviors of shelter dogs may affect their length of stay at the shelter. Controlling for morphological preferences, they found that leaning or rubbing on the kennel wall, facing away from the front of the kennel, and standing, were all associated with an increase in length of stay compared to dogs who did not exhibit these behaviors. No association between length of time at the shelter and consistent behavioral changes was found. The authors conclude that their findings can help shelters to focus their behavioral modification efforts on behaviors likely to influence adopters’ choices [39].

Döring and colleagues report on rehoming of laboratory beagles in Germany. They cite ‘The European Directive 2010/63/EU (EU, 2010; recital no. 26)’ that states as follows: “(…) animals such as dogs and cats should be allowed to be rehomed in families since there is a high level of public concern about the fate of such animals”. The adopted laboratory beagles, most of whom had never known life outside their laboratory kennel, showed desired behavior in their new homes within 6–12 weeks and thus proved to be highly adaptable. Nine dogs were returned, resulting in a 94% adoption success rate. This study showed that the rehoming of laboratory dogs presents a valuable alternative to euthanasia [40].

Lee and colleagues report on owner-reported characteristics of older dogs. This research is part of the Dog Aging Project. Owners reported that older dogs were less active than younger dogs; rural dogs were more active than suburban and urban dogs, especially at younger ages; and larger dogs were more active than smaller dogs. Somewhat surprisingly, older owners were found to have more active dogs than younger owners [41]. Related to Lee et al.’s study, Yamasaki reports on efforts by dog welfare organizations to motivate people to adopt older dogs. The approach involves a narrative that describes older dogs as potentially damaged but always resilient, deserving of care and still capable of a meaningful life [44].

#### 4.1.7. Dog Emotion as a Dog Welfare Issue

The emotions of dogs, especially negative emotions, were linked to dog welfare by several authors. Hakanen and colleagues (2020) investigated correlates of non-social fear in dogs. Non-social fear is an important welfare issue, as it causes distress in fearful dogs. It was found that less socialization early in life, inexperienced owners, living without other dogs present, urban environments, and less frequent participation in activities or training were all factors predictive of non-social fear. The authors suggest that several of these factors can be improved upon by changes in owner behavior [45]. A survey study conducted in Brazil showed that negative emotional activation in pet dogs was associated with single-dog households, with being neutered, and with being owned by women. Mixed breeds, which account for most of the pet dogs in Brazil, showed higher levels in both negative and positive emotional activation compared to purebred dogs [46].

Lenkei et al. surveyed personality traits of owners and their dogs and observed separation behavior with an outdoor test. Dogs with lenient owners were more likely to bark than to whine during the test, and the authors concluded that the owner’s attitude toward the dog can be related to the dog’s frustration-related separation behavior [47].

A survey administered in Tokyo, Osaka, and Sendai in Japan comprising owners of 262 dogs revealed that more than half of the owners reported that their dog showed anxiety-related behavior, and more than 20% of the owners were concerned about it. Triggers for anxiety-related behavior reported by owners included ‘separation’ and ‘subject’, ‘storm’ and ‘fireworks’, ‘storm’ and ‘sound’, and ‘fireworks’ and ‘sound’ [49]. A mixed-methods study in Iran used surveys and a behavioral test to investigate stress and fear-related factors and behavioral problems in dogs. The results showed that neuroticism and fear caused by other dogs and humans were commonly associated with problem behavior, followed by separation from the owner. Small dogs were more likely to show problem behavior linked to separation and fear caused by other dogs than larger dogs. Keeping dogs indoors with limited access to a yard was also found to be associated with problem behavior [51].

### 4.2. Human–Dog Bond Research across the Globe

We found that the number of annual publications on the human–dog bond increased significantly over the period 2012–2022, and that this upward trend continued in 2023. We also found that research on the human–dog bond takes place around the globe, with significant concentrations of this work in Europe and North America. We recognize that it is possible that publications represented in the database that we searched for this review are biased in origin toward work from Western, Educated, Industrialized, Rich, Democratic (WEIRD) societies. As such, our findings concerning the geographical distribution of work on the human–dog bond are preliminary. More work should be done using other sources to uncover research on the realities of dog welfare and the human–dog bond in non-WEIRD countries.

#### 4.2.1. Focus on Dog Welfare in Context

We found that Europe had the highest proportion of publications on the human–dog bond with a primary focus on dog welfare, followed by North America and Asia. Science is never conducted in a societal or political vacuum, and future research should study local animal welfare legislation as a societal context that could motivate or necessitate research on dog welfare. Moreover, in democracies, legislation derives from societal needs and sentiments, and as such, the presence or absence of animal welfare legislation specific to dogs can give insight into a democratic society’s perception of the place of dogs within the society.

We conducted some preliminary research on animal welfare legislation, using the World Animal Protection website https://api.worldanimalprotection.org/ accessed on 23 May 2024, and we found that, for example, Austria and The Netherlands, like other EU member states, ban modifying surgery on dogs, such as the cropping of ears and docking of tails in certain breeds, as well as the use of E-collars and electric fences and prong collars. Austria’s animal welfare laws specifically mention the mental wellbeing of animals, and in The Netherlands, dog welfare is being lobbied for by a 112-year-old organization for the protection of dogs (Koninklijke Honden Bescherming; https://hondenbescherming.nl/ accessed on 23 May 2024). The US is one of the pioneers in animal welfare legislation with the 1966 Animal Welfare Act, but much of this legislation is focused on farm- and industry-related animal practices. The use of E-collars and prong collars has not been banned in the US, nor has the practice of modifying surgeries on dogs.

#### 4.2.2. Dog Welfare and the Function of Dogs

Our review suggests that function matters in terms of dog welfare. For example, a study conducted in 124 globally distributed societies showed that human–dog bonds were closer, and positive care of the dogs increased, in the case of herding dogs, while the opposite was found for hunting dogs [7]. Research on culturally specific functions of dogs has the potential to tell us much about cultural diversity in the expression of the human–dog bond and how it relates to dog welfare.

In WEIRD societies with breed associations and kennel clubs in place, there is a need, in our view, for breed-specific investigations of dog welfare as an aspect of the bond between owners and handlers of the breed. The way in which the original function of a breed is maintained (or not) through specialized training and specific breeding requirements, such as done within the member states of the WUSV (Weltunion der Vereine für Deutsche Schäferhunde; World Union of German Shepherd Dog Club Associations), also deserves attention. Does formal adherence to a functional breed standard affect the welfare of the dogs, and, if so, in what way, and how is it related to the nature of the bond between owners and handlers of the breed? We think that these are questions worthy of pursuing in future research.

#### 4.2.3. Reciprocity and Individual Differences

Studies reviewed here illustrate reciprocity between humans and dogs in the establishment and sustenance of the human–dog bond, and they shed a light on how that interplay affects dog welfare. The shared feelings, interests and experiences that build the human–dog bond vary with the input of both partners, and there is a need for research on how the interplay of the abilities, predispositions, and characteristics of the partners affects the bond and dog welfare. There is no generic dog as much as there is no generic human, and to better understand the reciprocity within the human–dog bond, there is a need for more attention to individual differences such as ‘personality’ factors in members of both species. We argue for a relational approach that does justice to uncovering universal patterns of interaction as well as to the variations thereof.

#### 4.2.4. Crossing Disciplinary Boundaries

This scoping review illustrates that the human–dog bond is a topic of interest across a range of scientific disciplines. Perhaps this should not come as a surprise, considering that dogs feature in so many different aspects of human life and human culture. Quite a few of the studies that we reviewed here were the result of cross-disciplinary collaboration, and we think that such collaboration will be important to take the research on dog welfare and the human–dog bond to the next phase.

## 5. Conclusions

This scoping review shows that during the past decade, 44.8% of publications on the human–dog bond did not mention dog welfare. This finding can be interpreted from either a ‘the glass is half empty’ or a ‘the glass is half full’ perspective. We opt for the latter, as this scoping review also shows that the proportion of publications with a primary focus on dog welfare more than doubled over the period 2018–2022 compared to the period 2012–2017 (11.8% vs. 24.2%). In addition, the number of annual publications on the human–dog bond, irrespective of the presence or absence of a focus on dog welfare, increased significantly during the latter half of the past decade, with the upward trend continuing in 2023. This scoping review also suggests that more than in previous years, studies are considering dogs as deserving partners capable of complex interactions with humans that go far beyond mere responses to reward or punishment. Context matters, however, and the main limitation of this scoping review is that it presents findings that are mostly restricted to research conducted in WEIRD societies. There certainly is a need for research on dog welfare and the human–dog bond in non-WEIRD societies. In addition, the work included in this review is primarily focused on pet dogs, and we need to know much more about dog welfare in working dogs and service and assistance dogs as it relates to the bonds of these active dogs with their owners, handlers, and beneficiaries.

In closing, we suggest that the ethologist Nico Tinbergen’s eminently useful four questions for the study of behavior can help guide us in our thinking about where to take the research on dog welfare and the human–dog bond next. Answers to questions like ‘What enhances, protects, and sustains dog welfare in the human dog bond?’ (proximate causation); ‘How does the human–dog bond develop and how does its development affect dog welfare?’ (ontogeny); ‘What is the function of the human–dog bond and how does function affect dog welfare?’ (function); and ‘How did the ability of dogs and humans to form close bonds evolve?’ (ultimate causation) could tell us a lot about our dogs as well as about ourselves. Much remains to be done.

## Figures and Tables

**Table 1:** Consideration of dog welfare in human–dog bond research publications (2012–2023; *N* = 706).

Year	Primary Focus	Secondary Focus	Dog Welfare Mentioned	Dog Welfare Not Mentioned	Total
2012	1	1	0	8	10
2013	0	0	4	10	14
2014	9	4	5	16	34
2015	4	6	8	28	46
2016	6	5	11	28	51
2017	13	15	21	37	86
2018	14	2	10	35	61
2019	19	13	23	46	101
2020	21	21	17	47	106
2021	28	28	24	33	113
2022	20	6	15	17	58
Total	135	102	138	305	680
%	19.9	15	20.3	44.8	100
2023	7	2	4	13	26
%	26.9	7.7	15.4	50	100

**Table 2:** Focus on dog welfare in human–dog bond articles by category of dog (2012–2023; *N* = 706).

Category	Pet Dogs	Working Dogs	Service Dogs	Mixed Sample	Total
Primary Focus	129	6	3	4	142
Secondary Focus	95	3	6	0	104
Dog Welfare Mentioned	105	4	27	6	142
Dog Welfare Not Mentioned.	241	11	51	15	318
Total	570	24	87	25	706

**Table 3:** Consideration of dog welfare in human–dog bond research publications (2012–2023; *N* = 706) by country and geographic region.

Africa	Primary	Secondary	Mentioned	Not Mentioned	Total
Burkina Faso		1			1
Egypt				1	1
Ethiopia			1		1
Nigeria			1		1
Rwanda			1		1
South Africa				1	1
Subtotal (%)	0 (0)	1 (16.6)	3 (50.0)	2 (33.3)	6 (100)
Asia	Primary	Secondary	Mentioned	Not Mentioned	Total
China				2	2
Hong Kong		1	1		2
India	1		2	3	6
Japan	2	2	4	11	19
Malaysia		1			1
Pakistan	1				1
Philippines		1			1
South Korea		1	1		2
Sri Lanka	1				1
Taiwan				1	1
Thailand	1				1
Subtotal (%)	6 (16.2)	6 (16.2)	8 (21.6)	17 (45.9)	37 (100)
Europe	Primary	Secondary	Mentioned	Not Mentioned	Total
Austria	1	2	4	15	22
Belgium		2		5	7
Czech Republic				4	4
Denmark	1		2	1	4
Finland	4	1			5
France	8	2	3	6	19
Germany	5	1	2	7	15
Greece		1			1
Hungary	3	2	2	11	18
Ireland				2	2
Italy	13	4	5	26	48
The Netherlands	2	2	5	10	19
Northern Ireland	1				1
Norway			1	1	2
Poland	2			2	4
Romania		1	1	1	3
Russia			1	1	2
Serbia			1		1
Slovenia	1				1
Spain	2	2	2	4	10
Sweden	4	1	1	8	14
Switzerland	1	1		5	7
UK	25	16	25	22	88
Subtotal (%)	73 (24.3)	41 (13.6)	55 (18.3)	132 (43.8)	301 (100)
Middle East	Primary	Secondary	Mentioned	Not Mentioned	Total
Iran	1			2	3
Subtotal (%)	1 (33.3)			2 (66.7)	3 (100)
North America	Primary	Secondary	Mentioned	Not Mentioned	Total
Canada	4	10	9	14	37
USA	47	29	50	114	240
Subtotal (%)	51 (18.4)	39 (14.1)	59 (21.3)	128 (46.2)	277 (100)
Oceania	Primary	Secondary	Mentioned	Not Mentioned	Total
Australia	6	8	8	26	48
New Zealand	1	1	1	1	4
Subtotal (%)	7 (13.5)	9 (17.3)	9 (17.3)	27 (51.9)	52 (100)
South America	Primary	Secondary	Mentioned	Not Mentioned	Total
Argentina		1	3	6	10
Brazil	1	4	1	3	9
Chile	1	1	2		4
Colombia	1				1
Mexico	1	2	2	1	6
Subtotal (%)	4 (13.3)	8 (26.7)	8 (26.7)	10 (33.3)	30 (100)

**Table 4:** Journals publishing three or more articles on the human–dog bond with a focus on dog welfare ranked by primary focus (2012–2023).

Journal	Primary Focus	Secondary Focus	Total
Animals	13	11	24
Scientific Reports	12	1	13
Frontiers in Veterinary Science	11	8	19
Journal of Veterinary Behavior	11	8	19
Journal of Applied Animal Welfare Science	9	1	10
Veterinary Clinics of N. America—Small Animal Practice	9	1	10
PLoS ONE	8	6	14
Veterinary Record	8	4	12
Veterinary Sciences	5	0	5
Applied Animal Behavior Science	4	2	6
Physiology and Behavior	4	0	4
Preventive Veterinary Medicine	3	1	4
Society and Animals	3	1	4
Animal Cognition	2	1	3
International Journal of Comparative Psychology	2	6	8
Topics in Companion Animal Medicine	2	2	4
Veterinary Journal	2	1	3
Anthrozoös	0	3	3
Journal of Comparative Pathology	0	3	3

**Table 5:** Focus on dog welfare in human–dog bond publications (2012–2023; *N* = 706) by scientific domain.

Science Domain:	Behavior	Welfare	Medical	Social	Veterinary
Primary Focus	37	13	10	16	66
Secondary Focus	21	1	26	23	33
Dog Welfare Mentioned	35	5	40	34	28
Dog Welfare Not Mentioned	93	5	88	99	33
Total	186	24	164	172	160

**Table 6:** Welfare domains in human–dog bond publications with a primary or secondary focus on dog welfare by category of dog (2012–2023; *n* = 246).

	Pet Dogs	Working Dogs	Service Dogs	Mixed Sample
Nutrition	14	1	0	0
Environment	17	0	0	0
Health	82	2	5	1
Behavior	103	5	3	3
Other	8	1	1	0
Total	224	9	9	4

**Table 7:** Welfare domains in human–dog bond articles with a primary or secondary focus on dog welfare by scientific domain (2012–2023; *n* = 246).

	*Behavior*	*Welfare*	*Medical*	*Social*	*Veterinary*
Nutrition	1	0	1	4	9
Environment	6	1	1	2	7
Health	9	0	27	7	47
Behavior	39	11	5	25	34
Other	3	2	2	1	2
Total	58	14	36	39	99

**Table 8:** (a) Behavioral interaction effects on dog welfare (welfare domain Behavior) in human–dog bond articles with a primary or secondary focus on dog welfare by scientific domain (2012–2023; *n* = 114). (b). Sources of evidence for the analysis presented in (a).

**(a)**							
***Science Domain:***		***Behavior***	***Welfare***	***Medical***	***Social***	***Veterinary***	***Total***
Behavioral interactional effects associated with:							
Abuse		0	1	0	0	0	1
Attachment		5	0	0	4	0	9
Synchrony		1	0	0	1	0	2
COVID-19 pandemic		1	0	0	0	1	2
Dog characteristics		2	1	3	2	0	8
Dog emotion		5	0	0	1	2	8
Dog preferences		2	0	0	0	0	2
Dog-related injuries		0	0	1	0	0	1
Dog training		3	1	1	2	4	11
Ethical/moral issues		1	0	0	1	0	2
Intraspecies interactions		6	0	0	0	0	6
Owner characteristics		7	3	0	13	11	34
Physical activity		0	0	0	0	0	2
Population management		1	0	0	0	0	1
Post-conflict behavior		0	0	0	1	0	1
Problem behaviors		5	3	0	0	8	16
Stress levels		0	1	0	0	0	1
Veterinary visits		0	1	0	0	5	6
Total		39	11	5	25	34	114
**(b)**							
***Scientific Domain***	***Behavior***	***Welfare***	***Medical***	***Social***		***Veterinary***	
Abuse	-	McMillan et al. [24]	-	-		-	
Attachment	Carreiro et al. [25]; Riggio et al. [26]; Saavedra-Aracena et al. [27]; Sipple et al. [28]; Thielke and Udell [29]	-	-	Lewis [30]; Solomon et al. [31]; Payne et al. [32]; Konok et al. [33];			
Behavioral synchrony	Duranton et al. [34]	-	-	Duranton et al. [35]		-	
COVID-19 pandemic	Brand et al. [36]	-	-	-		Sherwell et al. [37]	
Dog characteristics	Samet et al. [38]; Protopopova et al. [39]	Döring et al. [40]	Lee et al. [41]; Smith [42]; Scotney and Clay [43]	Chira et al. [7]; Yamasaki [44]		-	
Dog emotion	Hakanen et al. [45]; Savalli et al. [46]; Lenkei et al. [47]; Arahori et al. [48]; Kurachi et al. [49]	-	-	McMillan [50]		Qiasvand et al. [51]; Ballantyne [52]	
Dog preferences	Bhattacharjee et al. [53]; Duranton et al. [54]	-	-	-		-	
Dog-related human injuries	-	-	Schurer et al. [55]	-		-	
Dog training	D’Angelo et al. [56]; Vieira de Castro et al. [57]; Harris et al. [58]	Vitulli et al. [59]	Alers & Simpson [60]	Smith et al. [61]; Pręgowski [23]		Townsend et al. [62]; Learn et al. [63]; LaFollette et al. [64]; Masson et al. [65];	
Ethical/moral issues	NG et al. [66]	-	-	Benz-Schwarzburg et al. [67]		-	
Intraspecies interaction	Cheng et al. [68]; Uccheddu et al. [69]; Cimarelli et al. [70]; Mariti et al. [71]; Romero et al. [72]; Mariti et al. [73]	-	-	-		-	
Owner/handler characteristics	Brubaker and Udell [74]; Holland et al. [75]; Stevens et al. [76]; Holland [16]; Philpotts et al. [77]; González-Ramírez [78]; Rehn et al. [79]	Reese [80]; Rossi and Maia [81]; Mehrkam et al. [82]	-	Ferrell and Crowley [83]; Giraudet et al. [84]; Karvinen and Rhodes [85]; Włodarczyk [86]; Bouma et al. [17]; Westgarth et al. [87]; Maharaj et al. [88]; Dodman et al. [89]; Davis et al. [90]; Hall et al. [91]; Bathurst and Lunghofer [92]; Sirois [93]; Alcaidinho et al. [94]		The Veterinary Record [95]; Powell et al. [96]; Pirrone [97]; Laurence et al. [98]; Hall et al. [99]; Nardoia et al. [100]; Strickler [101]; McGreevy et al. [102]; Diverio et al. [103]; Herron et al. [104]; Tiplady et al. [105];	
Physical activity	-	-	-	-		Väätäjä et al. [106]; Yuma et al. [107]	
Population management	Ma et al. [108]	-	-	-		-	
Post-conflict behavior	-	-	-	Cavalli et al. [109]		-	
Problem behavior	Feuerbacher and Muir [110]; Gates et al. [111]; Wormald et al. [112]; Thielke and Udell [113]; Mills et al. [114]	Stephens-Lewis et al. [115]; Bräm Dubé et al. [116]; Mehrkam et al. [117]	-	-		Powell et al. [118]; Normando et al. [119]; Teixeira and Hall [120]; Taylor et al. [121]; Buller and Ballantine [122]; Dinwoodie et al. [123]; Canejo-Teixeira et al. [124]; Rajapaksha [125]	
Stress levels	-	Koda et al. [126]	-	-		Riggio et al. [127]	
Veterinary visits	-	Mariti et al. [128]	-	-		Helsly et al. [129]; Kogan et al. [130]; Csoltova et al. [131]; Döring et al. [132]; Martin et al. [133]	

## Data Availability

Not applicable.

## Supplementary Materials

The following supporting information can be downloaded at: https://www.mdpi.com/article/10.3390/ani14131985/s1, Selected articles on the human–dog bond without a focus on dog welfare.
