# Supplementary material for: The Welfare of Dogs as an Aspect of the Human–Dog Bond: A Scoping Review
Source: Animals (Basel). 2024 Jul 5;14(13):1985. doi: 10.3390/ani14131985 (PMC11240373; doi:10.3390/ani14131985)
Supplement: Supplementary file 1 [file animals-14-01985-s001.zip › animals-3050724-supplementary.pdf]

animals MDPI

1. 'Unbreakable bond' celebrated by Friends for Life competition. *Vet Rec* **2014**, *174*, 273, doi:10.1136/vr.g2117.
2. The importance of the human-animal bond. *Vet Rec* **2018**, *183*, 638-639, doi:10.1136/vr.k5064.
3. Do my treatment decisions make me a 'bad' pet owner? *The Veterinary record* **2019**, *184*, 323, doi:10.1136/vr.l1073.
4. The growth of pet obesity. *Vet Rec* **2019**, *185*, 1-3, doi:10.1136/vr.l6498.
5. Lean on me: the use of therapy animals. *Vet Rec* **2019**, *185*, 750-752, doi:10.1136/vr.l7035.
6. Abdai, J.; Gergely, A.; Petro, E.; Topal, J.; Miklosi, A. An Investigation on Social Representations: Inanimate Agent Can Mislead Dogs (Canis familiaris) in a Food Choice Task. *PLoS One* **2015**, *10*, e0134575, doi:10.1371/journal.pone.0134575.
7. Aboul-Enein, B.H.; Puddy, W.C.; Bowser, J.E. The 1925 Diphtheria Antitoxin Run to Nome - Alaska: A Public Health Illustration of Human-Animal Collaboration. *J Med Humanit* **2019**, *40*, 287-296, doi:10.1007/s10912-016-9428-y.
8. Adame-Gomez, R.; Gisela Rodrigez-Romero, M.; Hilario-Alejandro, I.; Alheli Pineda-Rodriguez, S.; Toribio-Jimenez, J.; Rodriguez-Bataz, E.; Ramirez-Peralta, A. Trichophyton species isolated from asymptomatic patients of the pet-owner pair in Mexico. *Curr Med Mycol* **2021**, *7*, 29-33, doi:10.18502/cmm.7.2.7029.
9. Akiyama, J.; Ohta, M. Hormonal and Neurological Aspects of Dog Walking for Dog Owners and Pet Dogs. *Animals (Basel)* **2021**, *11*, doi:10.3390/ani11092732.
10. Amaya, V.; Paterson, M.B.A.; Phillips, C.J.C. Effects of Olfactory and Auditory Enrichment on the Behaviour of Shelter Dogs. *Animals (Basel)* **2020**, *10*, doi:10.3390/ani10040581.
11. Amici, F.; Waterman, J.; Kellermann, C.M.; Karimullah, K.; Brauer, J. The ability to recognize dog emotions depends on the cultural milieu in which we grow up. *Sci Rep* **2019**, *9*, 16414, doi:10.1038/s41598-019-52938-4.
12. Amon, M.J.; Favela, L.H. Distributed cognition criteria: Defined, operationalized, and applied to human-dog systems. *Behav Processes* **2019**, *162*, 167-176, doi:10.1016/j.beproc.2019.03.001.
13. Anyiam, F.; Lechenne, M.; Mindekem, R.; Oussigere, A.; Naissengar, S.; Alfaroukh, I.O.; Mbilo, C.; Moto, D.D.; Coleman, P.G.; Probst-Hensch, N.; et al. Cost-estimate and proposal for a development impact bond for canine rabies elimination by mass vaccination in Chad. *Acta Trop* **2017**, *175*, 112-120, doi:10.1016/j.actatropica.2016.11.005.
14. Anza-Burgess, K.; Lepofsky, D.; Yang, D. "A Part of the People": Human-Dog Relationships among the Northern Coast Salish of SW British Columbia. *Journal of Ethnobiology* **2023**, *40*, 434-450, doi:10.2993/0278-0771-40.4.434.
15. Applebaum, J.W.; Tomlinson, C.A.; Matijczak, A.; McDonald, S.E.; Zsembik, B.A. The Concerns, Difficulties, and Stressors of Caring for Pets during COVID-19: Results from a Large Survey of U.S. Pet Owners. *Animals (Basel)* **2020**, *10*, doi:10.3390/ani10101882.
16. Arenas-Montes, J.; Perez-Martinez, P.; Vals-Delgado, C.; Romero-Cabrera, J.L.; Cardelo, M.P.; Leon-Acuna, A.; Quintana-Navarro, G.M.; Alcala-Diaz, J.F.; Lopez-Miranda, J.; Camargo, A.; et al. Owning a Pet Is Associated with Changes in the Composition of Gut Microbiota and Could Influence the Risk of Metabolic Disorders in Humans. *Animals (Basel)* **2021**, *11*, doi:10.3390/ani11082347.
17. Arkow, P. Human-Animal Relationships and Social Work: Opportunities Beyond the Veterinary Environment. *Child Adolesc Social Work J* **2020**, *37*, 573-588, doi:10.1007/s10560-020-00697-x.
18. Ashall, V.; Hobson-West, P. 'Doing good by proxy': human-animal kinship and the 'donation' of canine blood. *Sociol Health Illn* **2017**, *39*, 908-922, doi:10.1111/1467-9566.12534.
19. Aufderheide, C.; Jalongo, M.R. Celebrating the Achievements of Prison Dog Programs. In *Prison Dog Programs*; 2019; pp. 275-303.
20. Auster, C.J.; Auster-Gussman, L.J.; Carlson, E.C. Lancaster Pet Cemetery Memorial Plaques 1951–2018: An Analysis of Inscriptions. *Anthrozoös* **2020**, *33*, 261-283, doi:10.1080/08927936.2020.1719766.
21. Avila-Alvarez, A.; Alonso-Bidegain, M.; De-Rosende-Celeiro, I.; Vizcaino-Cela, M.; Larraneta-Alcalde, L.; Torres-Tobio, G. Improving social participation of children with autism spectrum disorder: Pilot testing of an early animal-assisted intervention in Spain. *Health Soc Care Community* **2020**, *28*, 1220-1229, doi:10.1111/hsc.12955.
22. Avolio, A.; Tan, I.; Butlin, M. Of cats and dogs and matters of the heart. *J Hypertens* **2017**, *35*, 718-720, doi:10.1097/HJH.0000000000001231.
23. Axelsson, E.L.; Fawcett, C. Humans' pupillary contagion extends to cats and dogs. *Soc Cogn Affect Neurosci* **2021**, *16*, 153-166, doi:10.1093/scan/nsaa138.
24. Baár, M. Prosthesis for the body and for the soul: the origins of guide dog provision for blind veterans in interwar Germany. *First World War Studies* **2015**, *6*, 81-98, doi:10.1080/19475020.2015.1047890.
25. Balconi, M.; Vanutelli, M.E. Hemodynamic (fNIRS) and EEG (N200) correlates of emotional inter-species interactions modulated by visual and auditory stimulation. *Sci Rep* **2016**, *6*, 23083, doi:10.1038/srep23083.
26. Balme, J.; O'Connor, S. Dingoes and Aboriginal social organization in Holocene Australia. *Journal of Archaeological Science: Reports* **2016**, *7*, 775-781, doi:10.1016/j.jasrep.2015.08.015.
27. Banton, S.; Baynham, A.; Pezzali, J.G.; von Massow, M.; Shoveller, A.K. Grains on the brain: A survey of dog owner purchasing habits related to grain-free dry dog foods. *PLoS One* **2021**, *16*, e0250806, doi:10.1371/journal.pone.0250806.
28. Barcelos, A.M.; Kargas, N.; Maltby, J.; Hall, S.; Mills, D.S. A framework for understanding how activities associated with dog ownership relate to human well-being. *Sci Rep* **2020**, *10*, 11363, doi:10.1038/s41598-020-68446-9.
29. Barcelos, A.M.; Kargas, N.; Packham, C.; Mills, D.S. Understanding the impact of dog ownership on autistic adults: implications for mental health and suicide prevention. *Sci Rep* **2021**, *11*, 23655, doi:10.1038/s41598-021-02504-8.

30. Barnard, S.; Passalacqua, C.; Pelosi, A.; Valsecchi, P.; Prato-Previde, E. Effects of breed group and development on dogs' willingness to follow a human misleading advice. *Anim Cogn* **2019**, *22*, 757-768, doi:10.1007/s10071-019-01272-3.
31. Barrera, G.; Giamal, Y.; Mustaca, A.; Bentosela, M. Association between housing type and gaze, sociability, and fear-appeasement responses in dogs. *Suma Psicologica* **2012**, *19*, 7-18.
32. Barrera, G.; Guillen-Salazar, F.; Bentosela, M. Still-face Effect in Dogs (Canis familiaris). A Pilot Study. *J Appl Anim Welf Sci* **2023**, *26*, 271-284, doi:10.1080/10888705.2021.1923493.
33. Barrios, C.L.; Gornall, V.; Bustos-Lopez, C.; Cirac, R.; Calvo, P. Creation and Validation of a Tool for Evaluating Caregiver Burnout Syndrome in Owners of Dogs (Canis lupus familiaris) Diagnosed with Behavior Disorders. *Animals (Basel)* **2022**, *12*, doi:10.3390/ani12091185.
34. Barroso, C.S.; Brown, K.C.; Laubach, D.; Souza, M.; Daugherty, L.M.; Dixson, M. Cat and/or Dog Ownership, Cardiovascular Disease, and Obesity: A Systematic Review. *Vet Sci* **2021**, *8*, doi:10.3390/vetsci8120333.
35. Bartges, J.; Kushner, R.F.; Michel, K.E.; Sallis, R.; Day, M.J. One Health Solutions to Obesity in People and Their Pets. *J Comp Pathol* **2017**, *156*, 326-333, doi:10.1016/j.jcpa.2017.03.008.
36. Bartosiewicz, L.; Daróczi-Szabó, M.; Gál, E. A dog's life: interpreting Migration Period dog burials from Hungary. *Anthropozoologica* **2023**, *58*, doi:10.5252/anthropozoologica2023v58a2.
37. Bastos, A.P.M.; Neilands, P.D.; Hassall, R.S.; Lim, B.C.; Taylor, A.H. Dogs Mentally Represent Jealousy-Inducing Social Interactions. *Psychol Sci* **2021**, *32*, 646-654, doi:10.1177/0956797620979149.
38. Batty, G.D.; Zaninotto, P.; Watt, R.G.; Bell, S. Associations of pet ownership with biomarkers of ageing: population based cohort study. *BMJ* **2017**, *359*, j5558, doi:10.1136/bmj.j5558.
39. Belshaw, Z.; Dean, R.; Asher, L. Could it be osteoarthritis? How dog owners and veterinary surgeons describe identifying canine osteoarthritis in a general practice setting. *Prev Vet Med* **2020**, *185*, 105198, doi:10.1016/j.prevetmed.2020.105198.
40. Belshaw, Z.; Dean, R.; Asher, L. Slower, shorter, sadder: a qualitative study exploring how dog walks change when the canine participant develops osteoarthritis. *BMC Vet Res* **2020**, *16*, 85, doi:10.1186/s12917-020-02293-8.
41. Belshaw, Z.; Dean, R.; Asher, L. "You can be blind because of loving them so much": the impact on owners in the United Kingdom of living with a dog with osteoarthritis. *BMC Vet Res* **2020**, *16*, 190, doi:10.1186/s12917-020-02404-5.
42. Ben-Sefer, E.; Shields, L. Animal Farm in healthcare: definitions, policies, laws and implications for health professionals. *J R Soc Med* **2021**, *114*, 171-177, doi:10.1177/0141076821996003.
43. Benitez, A.D.N.; Monica, T.C.; Miura, A.C.; Romanelli, M.S.; Giordano, L.G.P.; Freire, R.L.; Mitsuka-Bregano, R.; Martins, C.M.; Biondo, A.W.; Serrano, I.M.; et al. Spatial and Simultaneous Seroprevalence of Anti-Leptospira Antibodies in Owners and Their Domiciled Dogs in a Major City of Southern Brazil. *Front Vet Sci* **2020**, *7*, 580400, doi:10.3389/fvets.2020.580400.
44. Benjamin, A.; Slocombe, K. 'Who's a good boy?!' Dogs prefer naturalistic dog-directed speech. *Anim Cogn* **2018**, *21*, 353-364, doi:10.1007/s10071-018-1172-4.
45. Bennett, N.E.; Gray, P.B. A Study Evaluating Consumer Motivations, Perceptions, and Responses to Direct-to-Consumer Canine Genetic Test Results. *Animals (Basel)* **2022**, *12*, doi:10.3390/ani12233360.
46. Bennetts, S.K.; Howell, T.; Crawford, S.; Burgemeister, F.; Burke, K.; Nicholson, J.M. Family Bonds with Pets and Mental Health during COVID-19 in Australia: A Complex Picture. *Int J Environ Res Public Health* **2023**, *20*, doi:10.3390/ijerph20075245.
47. Bentosela, M.; Wynne, C.D.; D'Orazio, M.; Elgier, A.; Udell, M.A. Sociability and gazing toward humans in dogs and wolves: Simple behaviors with broad implications. *J Exp Anal Behav* **2016**, *105*, 68-75, doi:10.1002/jeab.191.
48. Bergen-Cico, D.; Smith, Y.; Wolford, K.; Gooley, C.; Hannon, K.; Woodruff, R.; Spicer, M.; Gump, B. Dog Ownership and Training Reduces Post-Traumatic Stress Symptoms and Increases Self-Compassion Among Veterans: Results of a Longitudinal Control Study. *J Altern Complement Med* **2018**, *24*, 1166-1175, doi:10.1089/acm.2018.0179.
49. Berns, G.S.; Brooks, A.; Spivak, M. Replicability and heterogeneity of awake unrestrained canine FMRI responses. *PLoS One* **2013**, *8*, e81698, doi:10.1371/journal.pone.0081698.
50. Berns, G.S.; Brooks, A.M.; Spivak, M. Scent of the familiar: an fMRI study of canine brain responses to familiar and unfamiliar human and dog odors. *Behav Processes* **2015**, *110*, 37-46, doi:10.1016/j.beproc.2014.02.011.
51. Berry, A.; Borgi, M.; Terranova, L.; Chiarotti, F.; Alleva, E.; Cirulli, F. Developing effective animal-assisted intervention programs involving visiting dogs for institutionalized geriatric patients: a pilot study. *Psychogeriatrics* **2012**, *12*, 143-150, doi:10.1111/j.1479-8301.2011.00393.x.
52. Bir, C.; Ortez, M.; Olynk Widmar, N.J.; Wolf, C.A.; Hansen, C.; Ouedraogo, F.B. Familiarity and Use of Veterinary Services by US Resident Dog and Cat Owners. *Animals (Basel)* **2020**, *10*, doi:10.3390/ani10030483.
53. Bishop, G.; Cooney, K.; Cox, S.; Downing, R.; Mitchener, K.; Shanan, A.; Soares, N.; Stevens, B.; Wynn, T. 2016 AAHA/IAAHPC End-of-Life Care Guidelines. *J Am Anim Hosp Assoc* **2016**, *52*, 341-356, doi:10.5326/JAAHA-MS-6637.
54. Black, J.; Belicki, K.; Emberley-Ralph, J.; McCann, A. Internalized versus externalized continuing bonds: Relations to grief, trauma, attachment, openness to experience, and posttraumatic growth. *Death Stud* **2022**, *46*, 399-414, doi:10.1080/07481187.2020.1737274.
55. Blazina, C.; Abrams, E. Working With Men and Their Dogs: How Context Informs Clinical Practice When the Bond Is Present in Males' Lives. In *Clinician's Guide to Treating Companion Animal Issues*; 2019; pp. 223-252.
56. Blazina, C.; Kogan, L. Do Men Underreport and Mask Their Emotional Attachment to Animal Companions? The Influence of Precarious Masculinity on Men[illegible] Bonds with Their Dogs. *Anthrozoös* **2019**, *32*, 51-64, doi:10.1080/08927936.2019.1550281.

57. Blazina, C.; O'Neil, J.M.; Denke, R. A new understanding of man's best friend: A proposed contextual model for the exploration of human–animal interaction among insecurely attached males. In *Men and Their Dogs: A New Understanding of Man's Best Friend*; Springer International Publishing: 2016; pp. 47-72.
58. Bogaerts, E.; Moons, C.P.H.; Van Nieuwerburgh, F.; Peelman, L.; Saunders, J.H.; Broeckx, B.J.G. Rejections in an non-purpose bred assistance dog population: Reasons, consequences and methods for screening. *PLoS One* **2019**, *14*, e0218339, doi:10.1371/journal.pone.0218339.
59. Bonanni, R.; Cafazzo, S. The Social Organisation of a Population of Free-Ranging Dogs in a Suburban Area of Rome. In *The Social Dog*; 2014; pp. 65-104.
60. Bongers, J.; Gutierrez-Quintana, R.; Stalin, C.E. Owner's Perception of Seizure Detection Devices in Idiopathic Epileptic Dogs. *Front Vet Sci* **2021**, *8*, 792647, doi:10.3389/fvets.2021.792647.
61. Borgi, M.; Cirulli, F. Pet Face: Mechanisms Underlying Human-Animal Relationships. *Front Psychol* **2016**, *7*, 298, doi:10.3389/fpsyg.2016.00298.
62. Borgi, M.; Cogliati-Dezza, I.; Brelsford, V.; Meints, K.; Cirulli, F. Baby schema in human and animal faces induces cuteness perception and gaze allocation in children. *Front Psychol* **2014**, *5*, 411, doi:10.3389/fpsyg.2014.00411.
63. Bourne, G. Not man's best friend. *Veterinary Record* **2017**, *181*, 632, doi:10.1136/vr.j5704.
64. Bowen, J.; Bulbena, A.; Fatjo, J. The Value of Companion Dogs as a Source of Social Support for Their Owners: Findings From a Pre-pandemic Representative Sample and a Convenience Sample Obtained During the COVID-19 Lockdown in Spain. *Front Psychiatry* **2021**, *12*, 622060, doi:10.3389/fpsyt.2021.622060.
65. Bowen, J.; Garcia, E.; Darder, P.; Arguelles, J.; Fatjo, J. The effects of the Spanish COVID-19 lockdown on people, their pets, and the human-animal bond. *J Vet Behav* **2020**, *40*, 75-91, doi:10.1016/j.jveb.2020.05.013.
66. Boya, U.O.; Dotson, M.J.; Hyatt, E.M. A comparison of dog food choice criteria across dog owner segments: an exploratory study. *International Journal of Consumer Studies* **2014**, *39*, 74-82, doi:10.1111/ijcs.12145.
67. Bradshaw, J.W.; Pullen, A.J.; Rooney, N.J. Why do adult dogs 'play'? *Behav Processes* **2015**, *110*, 82-87, doi:10.1016/j.beproc.2014.09.023.
68. Brahams, D. How pets can reduce feelings of isolation and loneliness during Covid-19 and beyond: A personal view from London. *Med Leg J* **2021**, *89*, 2-3, doi:10.1177/0025817220980684.
69. Bray, E.E.; Sammel, M.D.; Cheney, D.L.; Serpell, J.A.; Seyfarth, R.M. Effects of maternal investment, temperament, and cognition on guide dog success. *Proc Natl Acad Sci U S A* **2017**, *114*, 9128-9133, doi:10.1073/pnas.1704303114.
70. Brown, B.B.; Jensen, W.A. Dog Ownership and Walking: Perceived and Audited Walkability and Activity Correlates. *Int J Environ Res Public Health* **2020**, *17*, doi:10.3390/ijerph17041385.
71. Bryant, D.T. Fibromyalgia, my dogs and me. *Veterinary Record* **2018**, *182*, 203, doi:10.1136/vr.k747.
72. Bryce, E.; Zurberg, T.; Zurberg, M.; Shajari, S.; Roscoe, D. Identifying environmental reservoirs of Clostridium difficile with a scent detection dog: preliminary evaluation. *J Hosp Infect* **2017**, *97*, 140-145, doi:10.1016/j.jhin.2017.05.023.
73. Burkhard, M.E.; Range, F.; Ward, S.J.; Robinson, L.M. Bonded by nature: Humans form equally strong and reciprocated bonds with similar raised dogs and wolves. *Front Psychol* **2022**, *13*, 1044940, doi:10.3389/fpsyg.2022.1044940.
74. Burton, A. What about a seizure-alert dog? *Lancet Neurol* **2017**, *16*, 265-266, doi:10.1016/S1474-4422(17)30050-9.
75. Bussolari, C.; Currin-McCulloch, J.; Packman, W.; Kogan, L.; Erdman, P. "I Couldn't Have Asked for a Better Quarantine Partner!": Experiences with Companion Dogs during Covid-19. *Animals (Basel)* **2021**, *11*, doi:10.3390/ani11020330.
76. Buttelmann, D.; Römpke, A.K. Anxiety-reducing effect: Dog, fish and plant in direct comparison. *Anthrozoos* **2014**, *27*, 267-277, doi:10.2752/175303714X13903827487647.
77. Calvo, P.; Bowen, J.; Bulbena, A.; Tobena, A.; Fatjo, J. Highly Educated Men Establish Strong Emotional Links with Their Dogs: A Study with Monash Dog Owner Relationship Scale (MDORS) in Committed Spanish Dog Owners. *PLoS One* **2016**, *11*, e0168748, doi:10.1371/journal.pone.0168748.
78. Campbell, J.; Dwyer, J.J.M.; Coe, J.B. Intervention Mapping to Develop a Print Resource for Dog-Walking Promotion in Canada. *J Vet Med Educ* **2017**, *44*, 234-246, doi:10.3138/jvme.1115-189R.
79. Capella Miternique, H.; Gaunet, F. Coexistence of Diversified Dog Socialities and Territorialities in the City of Concepcion, Chile. *Animals (Basel)* **2020**, *10*, doi:10.3390/ani10020298.
80. Capparelli, A.L.; Miller, Q.C.; Wright, D.B.; London, K. Canine-Assisted Interviews Bolster Informativeness for Negative Autobiographical Memories. *Psychol Rep* **2020**, *123*, 159-178, doi:10.1177/0033294119851803.
81. Carballo, F.; Dzik, V.; Freidin, E.; Damian, J.P.; Casanave, E.B.; Bentosela, M. Do dogs rescue their owners from a stressful situation? A behavioral and physiological assessment. *Anim Cogn* **2020**, *23*, 389-403, doi:10.1007/s10071-019-01343-5.
82. Carballo, F.; Freidin, E.; Putrino, N.; Shimabukuro, C.; Casanave, E.; Bentosela, M. Dog's discrimination of human selfish and generous attitudes: the role of individual recognition, experience, and experimenters' gender. *PLoS One* **2015**, *10*, e0116314, doi:10.1371/journal.pone.0116314.
83. Cardoso, S.D.; Faraco, C.B.; de Sousa, L.; Pereira, G.D.G. Empathy with humans and with non-human animals: are there differences between individuals who have adopted and those who have relinquished a pet? *Journal of Veterinary Behavior* **2022**, *49*, 46-52, doi:10.1016/j.jveb.2021.11.008.
84. Carlin, E.P.; Tyungu, D.L. Toxocara: Protecting pets and improving the lives of people. *Adv Parasitol* **2020**, *109*, 3-16, doi:10.1016/bs.apar.2020.01.001.

85. Carlisle, G.K. The social skills and attachment to dogs of children with autism spectrum disorder. *J Autism Dev Disord* **2015**, *45*, 1137-1145, doi:10.1007/s10803-014-2267-7.
86. Carlisle, G.K.; Johnson, R.A.; Mazurek, M.; Bibbo, J.L.; Tocco, F.; Cameron, G.T. Companion animals in families of children with autism spectrum disorder: Lessons learned from caregivers. *Journal of Family Social Work* **2017**, *21*, 294-312, doi:10.1080/10522158.2017.1394413.
87. Carlsen, O.C.L.; Gudmundsdottir, H.K.; Bains, K.E.S.; Bertelsen, R.; Carlsen, K.C.L.; Carlsen, K.H.; Endre, K.M.A.; Granum, B.; Haugen, G.; Hedlin, G.; et al. Physical activity in pregnancy: a Norwegian-Swedish mother-child birth cohort study. *AJOG Glob Rep* **2021**, *1*, 100002, doi:10.1016/j.xagr.2020.100002.
88. Carlyle, D.; Graham, P. Bodies of Knowledge, Kinetic Melodies, Rhythms of Relating and Affect Attunement in Vital Spaces for Multi-Species Well-Being: Finding Common Ground in Intimate Human-Canine and Human-Equine Encounters. *Animals (Basel)* **2019**, *9*, doi:10.3390/ani9110934.
89. Carr, E.C.J.; Norris, J.M.; Alix Hayden, K.; Pater, R.; Wallace, J.E. A Scoping Review of the Health and Social Benefits of Dog Ownership for People Who Have Chronic Pain. *Anthrozoös* **2020**, *33*, 207-224, doi:10.1080/08927936.2020.1719761.
90. Carr, E.C.J.; Wallace, J.E.; Onyewuchi, C.; Hellyer, P.W.; Kogan, L. Exploring the Meaning and Experience of Chronic Pain with People Who Live with a Dog: A Qualitative Study. *Anthrozoös* **2018**, *31*, 551-565, doi:10.1080/08927936.2018.1505267.
91.
92. Catala, A.; Latour, P.; Cousillas, H.; Hausberger, M.; Grandgeorge, M. Is there a Profile of Spontaneous Seizure-Alert Pet Dogs? A Survey of French People with Epilepsy. *Animals (Basel)* **2020**, *10*, doi:10.3390/ani10020254.
93. Cavalli, C.; Carballo, F.; Dzik, M.V.; Bentosela, M. Showing behavior in Animal Assisted Intervention and pet dogs. *Behav Processes* **2020**, *179*, 104218, doi:10.1016/j.beproc.2020.104218.
94. Chalmers, D.; Dell, C.A. Applying One Health to the Study of Animal-Assisted Interventions. *Ecohealth* **2015**, *12*, 560-562, doi:10.1007/s10393-015-1042-3.
95. Chambers, J.; Quinlan, M.B.; Evans, A.; Quinlan, R.J. Dog-Human Coevolution: Cross-Cultural Analysis of Multiple Hypotheses. *Journal of Ethnobiology* **2023**, *40*, 414-433, doi:10.2993/0278-0771-40.4.414.
96. Chan, H.W.; Wong, D.F.K. Effects of Companion Dogs on Adult Attachment, Emotion Regulation, and Mental Wellbeing in Hong Kong. *Society & Animals* **2022**, *30*, 668-688, doi:10.1163/15685306-bja10091.
97. Chan, M.M.; Tapia Rico, G. The "pet effect" in cancer patients: Risks and benefits of human-pet interaction. *Crit Rev Oncol Hematol* **2019**, *143*, 56-61, doi:10.1016/j.critrevonc.2019.08.004.
98. Chen, C.M.; Tang, H.L.; Chiou, C.S.; Tung, K.C.; Lu, M.C.; Lai, Y.C. Colonization dynamics of Klebsiella pneumoniae in the pet animals and human owners in a single household. *Vet Microbiol* **2021**, *256*, 109050, doi:10.1016/j.vetmic.2021.109050.
99. Cherkaev, X.; Tipikina, E. Interspecies Affection and Military Aims. *Environmental Humanities* **2018**, *10*, 20-39, doi:10.1215/22011919-4385453.
100. Christian, H.; Mitrou, F.; Cunneen, R.; Zubrick, S.R. Pets Are Associated with Fewer Peer Problems and Emotional Symptoms, and Better Prosocial Behavior: Findings from the Longitudinal Study of Australian Children. *J Pediatr* **2020**, *220*, 200-206 e202, doi:10.1016/j.jpeds.2020.01.012.
101. Christiansen, S.B.; Kristensen, A.T.; Lassen, J.; Sandoe, P. Veterinarians' role in clients' decision-making regarding seriously ill companion animal patients. *Acta Vet Scand* **2016**, *58*, 30, doi:10.1186/s13028-016-0211-x.
102. Christiansen, S.B.; Kristensen, A.T.; Sandøe, P.; Lassen, J. Looking After Chronically Ill Dogs: Impacts on the Caregiver's Life. *Anthrozoös* **2015**, *26*, 519-533, doi:10.2752/175303713x13795775536174.
103. Chubak, J.; Hawkes, R. Animal-Assisted Activities: Results From a Survey of Top-Ranked Pediatric Oncology Hospitals. *J Pediatr Oncol Nurs* **2016**, *33*, 289-296, doi:10.1177/1043454215614961.
104. Chumley, P.R. Historical perspectives of the human-animal bond within the Department of Defense. *U.S. Army Medical Department journal* **2012**, 18-20.
105. Churchill, J.; Ward, E. Communicating with Pet Owners About Obesity: Roles of the Veterinary Health Care Team. *Vet Clin North Am Small Anim Pract* **2016**, *46*, 899-911, doi:10.1016/j.cvsm.2016.04.010.
106. Chutter, M.; Perry, P.; Houpt, K. Efficacy of fluoxetine for canine behavioral disorders. *Journal of Veterinary Behavior* **2019**, *33*, 54-58, doi:10.1016/j.jveb.2019.05.006.
107. Cimarelli, G.; Schindlbauer, J.; Pegger, T.; Wesian, V.; Viranyi, Z. Secure base effect in former shelter dogs and other family dogs: Strangers do not provide security in a problem-solving task. *PLoS One* **2021**, *16*, e0261790, doi:10.1371/journal.pone.0261790.
108. Cimarelli, G.; Turcsan, B.; Banlaki, Z.; Range, F.; Viranyi, Z. Dog Owners' Interaction Styles: Their Components and Associations with Reactions of Pet Dogs to a Social Threat. *Front Psychol* **2016**, *7*, 1979, doi:10.3389/fpsyg.2016.01979.
109. Cimarelli, G.; Turcsan, B.; Range, F.; Viranyi, Z. The Other End of the Leash: An Experimental Test to Analyze How Owners Interact with Their Pet Dogs. *J Vis Exp* **2017**, doi:10.3791/56233.
110. Cohen, S.I. A Dying Man's Wish Fulfilled. *Tex Heart Inst J* **2016**, *43*, 285-286, doi:10.14503/THIJ-16-5834.
111. Coleman, J.A.; Green, B.; Garthe, R.C.; Worthington, E.L.; Barker, S.B.; Ingram, K.M. The Coleman Dog Attitude Scale (C-DAS): Development, refinement, validation, and reliability. *Applied Animal Behaviour Science* **2016**, *176*, 77-86, doi:10.1016/j.applanim.2016.01.003.
112. Compitus, K. Traumatic pet loss and the integration of attachment-based animal assisted therapy. *Journal of Psychotherapy Integration* **2019**, *29*, 119-131, doi:10.1037/int0000143.

113. Contreras-Abarca, R.; Crespin, S.J.; Moreira-Arce, D.; Simonetti, J.A. Redefining feral dogs in biodiversity conservation. *Biological Conservation* **2022**, *265*, doi:10.1016/j.biocon.2021.109434.
114. Cook, A.; Arter, J.; Jacobs, L.F. My owner, right or wrong: the effect of familiarity on the domestic dog's behavior in a food-choice task. *Anim Cogn* **2014**, *17*, 461-470, doi:10.1007/s10071-013-0677-0.
115. Cook, P.F.; Prichard, A.; Spivak, M.; Berns, G.S. Awake canine fMRI predicts dogs' preference for praise vs food. *Soc Cogn Affect Neurosci* **2016**, *11*, 1853-1862, doi:10.1093/scan/nsw102.
116. Coombs, S.; Eberlein, A.; Mantata, K.; Turnhout, A.; Smith, C.M. Did dog ownership influence perceptions of adult health and wellbeing during and following the Canterbury earthquakes? A qualitative study. *Australasian Journal of Disaster and Trauma Studies* **2015**, *19*, 67-76.
117. Cooney, K.A.; Kogan, L.R.; Brooks, S.L.; Ellis, C.A. Pet Owners' Expectations for Pet End-of-Life Support and After-Death Body Care: Exploration and Practical Applications. *Top Companion Anim Med* **2021**, *43*, 100503, doi:10.1016/j.tcam.2020.100503.
118. Cordoni, G.; Palagi, E. Back to the Future: A Glance Over Wolf Social Behavior to Understand Dog-Human Relationship. *Animals (Basel)* **2019**, *9*, doi:10.3390/ani9110991.
119. Corkran, C.M. “An Extension of Me”. *Society & Animals* **2015**, *23*, 231-249, doi:10.1163/15685306-12341252.
120. Correa, G.F.; Barcelos, A.M.; Mills, D.S. Dog-related activities and human well-being in Brazilian dog owners: A framework and cross-cultural comparison with a British study. *Sci Prog* **2021**, *104*, 368504211050277, doi:10.1177/00368504211050277.
121. Cowling, D.M.; Isenstein, S.G.E.; Schneider, M.S. When the Bond Breaks: Variables Associated with Grief Following Companion Animal Loss. *Anthrozoös* **2020**, *33*, 693-708, doi:10.1080/08927936.2020.1824651.
122. Coy, A.E.; Green, J.D.; Behler, A.M.C. Why Can't I Resist Those "Puppy Dog" (or "Kitty Cat") Eyes? A Study of Owner Attachment and Factors Associated with Pet Obesity. *Animals (Basel)* **2021**, *11*, doi:10.3390/ani11020539.
123. Craigon, P.J.; Hobson-West, P.; England, G.C.W.; Whelan, C.; Lethbridge, E.; Asher, L. "She's a dog at the end of the day": Guide dog owners' perspectives on the behaviour of their guide dog. *PLoS One* **2017**, *12*, e0176018, doi:10.1371/journal.pone.0176018.
124. Creagan, E.T.; Bauer, B.A.; Thomley, B.S.; Borg, J.M. Animal-assisted therapy at Mayo Clinic: The time is now. *Complement Ther Clin Pract* **2015**, *21*, 101-104, doi:10.1016/j.ctcp.2015.03.002.
125. Cristescu, R.H.; Miller, R.L.; Frère, C.H. Sniffing out solutions to enhance conservation: How detection dogs can maximise research and management outcomes, through the example of koalas. *Australian Zoologist* **2020**, *40*, 416-432, doi:10.7882/az.2019.030.
126. Crossman, M.K.; Kazdin, A.E. Additional Evidence is Needed to Recommend Acquiring a Dog to Families of Children with Autism Spectrum Disorder: A Response to Wright and Colleagues. *J Autism Dev Disord* **2016**, *46*, 332-335, doi:10.1007/s10803-015-2542-2.
127. Curl, A.L.; Bibbo, J.; Johnson, R.A. Dog Walking, the Human-Animal Bond and Older Adults' Physical Health. *Gerontologist* **2017**, *57*, 930-939, doi:10.1093/geront/gnw051.
128. Curl, A.L.; Bibbo, J.; Johnson, R.A. Neighborhood Engagement, Dogs, and Life Satisfaction in Older Adulthood. *J Appl Gerontol* **2021**, *40*, 1706-1714, doi:10.1177/0733464820953725.
129. d'Angelo, D.; Ciani, F.; Zaccherini, A.; Tafuri, S.; Avallone, L.; d'Ingeo, S.; Quaranta, A. Human-Animal Relationship Dysfunction: A Case Study of Animal Hoarding in Italy. *Animals (Basel)* **2020**, *10*, doi:10.3390/ani10091501.
130.
131. D'Aniello, B.; Scandurra, A.; Alterisio, A.; Valsecchi, P.; Prato-Previde, E. The importance of gestural communication: a study of human-dog communication using incongruent information. *Anim Cogn* **2016**, *19*, 1231-1235, doi:10.1007/s10071-016-1010-5.
132. D'Aniello, B.; Scandurra, A.; Pinelli, C.; Marinelli, L.; Mongillo, P. Is this love? Sex differences in dog-owner attachment behavior suggest similarities with adult human bonds. *Anim Cogn* **2022**, *25*, 137-148, doi:10.1007/s10071-021-01545-w.
133. D'Aniello, B.; Scandurra, A.; Prato-Previde, E.; Valsecchi, P. Gazing toward humans: a study on water rescue dogs using the impossible task paradigm. *Behav Processes* **2015**, *110*, 68-73, doi:10.1016/j.beproc.2014.09.022.
134. D'Aniello, B.; Semin, G.R.; Alterisio, A.; Aria, M.; Scandurra, A. Interspecies transmission of emotional information via chemosignals: from humans to dogs (Canis lupus familiaris). *Anim Cogn* **2018**, *21*, 67-78, doi:10.1007/s10071-017-1139-x.
135. de Albuquerque, N.S.; Costa, D.B.; dos Reis Rodrigues, G.; Sessegolo, N.S.; Moret-Tatay, C.; Irigaray, T.Q. Adaptation and psychometric properties of Lexington Attachment to Pets Scale: Brazilian version (LAPS-B). *Journal of Veterinary Behavior* **2023**, *61*, 50-56, doi:10.1016/j.jveb.2022.12.005.
136. Degeling, C.; Brookes, V.; Lea, T.; Ward, M. Rabies response, One Health and more-than-human considerations in Indigenous communities in northern Australia. *Soc Sci Med* **2018**, *212*, 60-67, doi:10.1016/j.socscimed.2018.07.006.
137. Delicano, R.A.; Hammar, U.; Egenvall, A.; Westgarth, C.; Mubanga, M.; Byberg, L.; Fall, T.; Kennedy, B. The shared risk of diabetes between dog and cat owners and their pets: register based cohort study. *BMJ* **2020**, *371*, m4337, doi:10.1136/bmj.m4337.
138. Dell, C.; Chalmers, D.; Stobbe, M.; Rohr, B.; Husband, A. Animal-assisted therapy in a Canadian psychiatric prison. *Int J Prison Health* **2019**, *15*, 209-231, doi:10.1108/IJPH-04-2018-0020.
139. Dell, C.; Williamson, L.; McKenzie, H.; Carey, B.; Cruz, M.; Gibson, M.; Pavelich, A. A Commentary about Lessons Learned: Transitioning a Therapy Dog Program Online during the COVID-19 Pandemic. *Animals (Basel)* **2021**, *11*, doi:10.3390/ani11030914.
140. Dell, C.A. Questioning "Fluffy": A Dog's Eye View of Animal-Assisted Interventions (AAI) in the Treatment of Substance Misuse. *Subst Use Misuse* **2015**, *50*, 1148-1152, doi:10.3109/10826084.2015.1007668.
141. Dell, C.A.; Kosteniuk, B.; Bentley, E. Benefits and Challenges of Having a Service Dog Among Veterans in Recovery from Substance Use Harms. *Anthrozoös* **2022**, *36*, 83-97, doi:10.1080/08927936.2022.2074190.

142. Deng, P.; Swanson, K.S. Companion animals symposium: Future aspects and perceptions of companion animal nutrition and sustainability. *Journal of Animal Science* **2015**, *93*, 823-834, doi:10.2527/jas.2014-8520.
143. Derosier, J. Dental therapy dogs. Can man's best friend help your practice? *CDS review* **2016**, *109*, 10-12.
144. Díaz Videla, M.; López, P.A. Oxytocin in the human-dog bond: Review of the literature and analysis of future investigation fields. *Interdisciplinaria* **2017**, *34*, 73-90.
145. Dingman, P.A.; Levy, J.K.; Rockey, L.E.; Crandall, M.M. Use of visual and permanent identification for pets by veterinary clinics. *Vet J* **2014**, *201*, 46-50, doi:10.1016/j.tvjl.2014.04.024.
146. Doring, D.; Nick, O.; Bauer, A.; Kuchenhoff, H.; Erhard, M.H. How do rehomed laboratory beagles behave in everyday situations? Results from an observational test and a survey of new owners. *PLoS One* **2017**, *12*, e0181303, doi:10.1371/journal.pone.0181303.
147. Downes, M.J.; Devitt, C.; Downes, M.T.; More, S.J. Understanding the context for pet cat and dog feeding and exercising behaviour among pet owners in Ireland: a qualitative study. *Ir Vet J* **2017**, *70*, 29, doi:10.1186/s13620-017-0107-8.
148. Dreger, D.L.; Davis, B.W.; Cocco, R.; Sechi, S.; Di Cerbo, A.; Parker, H.G.; Polli, M.; Marelli, S.P.; Crepaldi, P.; Ostrander, E.A. Commonalities in Development of Pure Breeds and Population Isolates Revealed in the Genome of the Sardinian Fonni's Dog. *Genetics* **2016**, *204*, 737-755, doi:10.1534/genetics.116.192427.
149. Duindam, H.M.; Creemers, H.E.; Hoeve, M.; Asscher, J.J. Who Lets the Dog In? Differential Effects of a Dog-Training Program for Incarcerated Adults. *Anthrozoos* **2021**, *34*, 839-861, doi:10.1080/08927936.2021.1938405.
150. Duranton, C.; Bedossa, T.; Gaunet, F. Pet dogs synchronize their walking pace with that of their owners in open outdoor areas. *Anim Cogn* **2018**, *21*, 219-226, doi:10.1007/s10071-017-1155-x.
151. Duranton, C.; Bedossa, T.; Gaunet, F. Pet dogs exhibit social preference for people who synchronize with them: what does it tell us about the evolution of behavioral synchronization? *Anim Cogn* **2019**, *22*, 243-250, doi:10.1007/s10071-019-01241-w.
152. Duranton, C.; Gaunet, F. Canis sensitivus: Affiliation and dogs' sensitivity to others' behavior as the basis for synchronization with humans? *Journal of Veterinary Behavior* **2015**, *10*, 513-524, doi:10.1016/j.jveb.2015.08.008.
153. Duranton, C.; Gaunet, F. Effects of shelter housing on dogs' sensitivity to human social cues. *Journal of Veterinary Behavior* **2016**, *14*, 20-27, doi:10.1016/j.jveb.2016.06.011.
154. Duranton, C.; Gaunet, F. Behavioral synchronization and affiliation: Dogs exhibit human-like skills. *Learn Behav* **2018**, *46*, 364-373, doi:10.3758/s13420-018-0323-4.
155. Dzik, M.V.; Barrera, G.; Bentosela, M. The relevance of oxytocin in the dog-human bond. *Interdisciplinaria* **2018**, *35*, 527-542.
156. Dzik, M.V.; Carballo, F.; Casanave, E.; Bentosela, M. Effects of oxytocin administration and the dog-owner bond on dogs' rescue behavior. *Anim Cogn* **2021**, *24*, 1191-1204, doi:10.1007/s10071-021-01512-5.
157. Eagan, B.H.; Gordon, E.; Protopopova, A. Reasons for Guardian-Relinquishment of Dogs to Shelters: Animal and Regional Predictors in British Columbia, Canada. *Front Vet Sci* **2022**, *9*, 857634, doi:10.3389/fvets.2022.857634.
158. Early, J.; Arnott, E.; Wilson, B.; Wade, C.; McGreevy, P. The Perceived Value of Behavioural Traits in Australian Livestock Herding Dogs Varies with the Operational Context. *Animals (Basel)* **2019**, *9*, doi:10.3390/ani9070448.
159. Eatherington, C.J.; Mongillo, P.; Looke, M.; Marinelli, L. Dogs (Canis familiaris) recognise our faces in photographs: implications for existing and future research. *Anim Cogn* **2020**, *23*, 711-719, doi:10.1007/s10071-020-01382-3.
160. Edwards, P.T.; Hazel, S.J.; Browne, M.; Serpell, J.A.; McArthur, M.L.; Smith, B.P. Investigating risk factors that predict a dog's fear during veterinary consultations. *PLoS One* **2019**, *14*, e0215416, doi:10.1371/journal.pone.0215416.
161. El-Qushayri, A.E.; Kamel, A.M.A.; Faraj, H.A.; Vuong, N.L.; Diab, O.M.; Istanbuly, S.; Elshafei, T.A.; Makram, O.M.; Sattar, Z.; Istanbuly, O.; et al. Association between pet ownership and cardiovascular risks and mortality: a systematic review and meta-analysis. *J Cardiovasc Med (Hagerstown)* **2020**, *21*, 359-367, doi:10.2459/JCM.0000000000000920.
162. Ellen Netting, F.; Wilson, C.C.; Goodie, J.L.; Stephens, M.B.; Byers, C.G.; Olsen, C.H. Attachment, social support, and perceived mental health of adult dog walkers: What does age have to do with it? *Journal of Sociology and Social Welfare* **2013**, *40*, 261-283.
163. Elmaci, D.T.; Cevizci, S. Dog-assisted therapies and activities in rehabilitation of children with cerebral palsy and physical and mental disabilities. *Int J Environ Res Public Health* **2015**, *12*, 5046-5060, doi:10.3390/ijerph120505046.
164. Endo, K.; Yamasaki, S.; Ando, S.; Kikusui, T.; Mogi, K.; Nagasawa, M.; Kamimura, I.; Ishihara, J.; Nakanishi, M.; Usami, S.; et al. Dog and Cat Ownership Predicts Adolescents' Mental Well-Being: A Population-Based Longitudinal Study. *Int J Environ Res Public Health* **2020**, *17*, doi:10.3390/ijerph17030884.
165. Ensminger, J.J.; Thomas, J.L. Writing Letters to Help Patients with Service and Support Animals. *Journal of Forensic Psychology Practice* **2013**, *13*, 92-115, doi:10.1080/15228932.2013.765734.
166. Esch, K.J.; Petersen, C.A. Transmission and epidemiology of zoonotic protozoal diseases of companion animals. *Clin Microbiol Rev* **2013**, *26*, 58-85, doi:10.1128/CMR.00067-12.
167. Essner, A.; Hogberg, H.; Zetterberg, L.; Hellstrom, K.; Sjostrom, R.; Gustas, P. Investigating the Probability of Response Bias in Owner-Perceived Pain Assessment in Dogs With Osteoarthritis. *Top Companion Anim Med* **2020**, *39*, 100407, doi:10.1016/j.tcam.2020.100407.
168. Ezell, J.M.; Cassidy-Bushrow, A.E.; Havstad, S.; Joseph, C.L.M.; Wegienka, G.; Jones, K.; Ownby, D.R.; Johnson, C.C. Prenatal dog-keeping practices vary by race: Speculations on implications for disparities in childhood health and disease. *Ethnicity and Disease* **2014**, *24*, 104-109.

169. Fecteau, S.M.; Boivin, L.; Trudel, M.; Corbett, B.A.; Harrell, F.E., Jr.; Viau, R.; Champagne, N.; Picard, F. Parenting stress and salivary cortisol in parents of children with autism spectrum disorder: Longitudinal variations in the context of a service dog's presence in the family. *Biol Psychol* **2017**, *123*, 187-195, doi:10.1016/j.biopsycho.2016.12.008.
170. Ferreira, A.; Alho, A.M.; Otero, D.; Gomes, L.; Nijsse, R.; Overgaauw, P.A.M.; Madeira de Carvalho, L. Urban Dog Parks as Sources of Canine Parasites: Contamination Rates and Pet Owner Behaviours in Lisbon, Portugal. *J Environ Public Health* **2017**, *2017*, 5984086, doi:10.1155/2017/5984086.
171. Ferreira, E.A.; Paloski, L.H.; Costa, D.B.; Fiametti, V.S.; De Oliveira, C.R.; de Lima, A., II; Gonzatti, V.; Irigaray, T.Q. Animal Hoarding Disorder: A new psychopathology? *Psychiatry Res* **2017**, *258*, 221-225, doi:10.1016/j.psychres.2017.08.030.
172. Feuerbacher, E.N.; Wynne, C.D. Shut up and pet me! Domestic dogs (Canis lupus familiaris) prefer petting to vocal praise in concurrent and single-alternative choice procedures. *Behav Processes* **2015**, *110*, 47-59, doi:10.1016/j.beproc.2014.08.019.
173. Feuerbacher, E.N.; Wynne, C.D. Application of functional analysis methods to assess human-dog interactions. *J Appl Behav Anal* **2016**, *49*, 970-974, doi:10.1002/jaba.318.
174. Feuerbacher, E.N.; Wynne, C.D.L. Dogs don't always prefer their owners and can quickly form strong preferences for certain strangers over others. *J Exp Anal Behav* **2017**, *108*, 305-317, doi:10.1002/jeab.280.
175. Fine, A.H. The role of therapy and service animals in the lives of persons with disabilities. *Rev Sci Tech* **2018**, *37*, 141-149, doi:10.20506/rst.37.1.2747.
176. Fine, A.H. The Year That Has Passed Us By: Animals in Our Life of COVID-19. *Animals (Basel)* **2021**, *11*, doi:10.3390/ani11020395.
177. Fine, A.H.; Mackintosh, T.K. Animal-Assisted Interventions: Entering a Crossroads of Explaining an Instinctive Bond under the Scrutiny of Scientific Inquiry. In *Encyclopedia of Mental Health*; 2016; pp. 68-73.
178. Fiset, S.; Plourde, V. Commentary: Oxytocin-Gaze Positive Loop and the Coevolution of Human-Dog Bonds. *Front Psychol* **2015**, *6*, 1845, doi:10.3389/fpsyg.2015.01845.
179. Flegr, J.; Preiss, M. Friends with malefit. The effects of keeping dogs and cats, sustaining animal-related injuries and Toxoplasma infection on health and quality of life. *PLoS One* **2019**, *14*, e0221988, doi:10.1371/journal.pone.0221988.
180. Fletcher, T.; Platt, L. (Just) a walk with the dog? Animal geographies and negotiating walking spaces. *Social & Cultural Geography* **2016**, *19*, 211-229, doi:10.1080/14649365.2016.1274047.
181. Forbes, C.C.; Blanchard, C.M.; Mummery, W.K.; Courneya, K.S. Dog ownership and physical activity among breast, prostate, and colorectal cancer survivors. *Psychooncology* **2017**, *26*, 2186-2193, doi:10.1002/pon.4324.
182. Franklin, R.G., Jr.; Nelson, A.J.; Baker, M.; Beeney, J.E.; Vescio, T.K.; Lenz-Watson, A.; Adams, R.B., Jr. Neural responses to perceiving suffering in humans and animals. *Soc Neurosci* **2013**, *8*, 217-227, doi:10.1080/17470919.2013.763852.
183. Fraser, M.; Girling, S.J. An evaluation of the experiences of guide dog owners visiting Scottish veterinary practices. *Vet Rec* **2016**, *179*, 253, doi:10.1136/vr.103626.
184. Friedman, E.; Krause-Parello, C.A. Companion animals and human health: benefits, challenges, and the road ahead for human-animal interaction. *Rev Sci Tech* **2018**, *37*, 71-82, doi:10.20506/rst.37.1.2741.
185. Furst, G. Prisoners, pups, and PTSD: the grass roots response to veterans with PTSD. *Contemporary Justice Review* **2015**, *18*, 449-466, doi:10.1080/10282580.2015.1093688.
186. Furst, G.; Houser, K. Hirschi's Social Bond Theory: how human-animal interactions explain the effectiveness of carceral dog training programs. *Journal of Offender Rehabilitation* **2021**, *60*, 291-310, doi:10.1080/10509674.2021.1931626.
187. Gadomski, A.; Scribani, M.B.; Tallman, N.; Krupa, N.; Jenkins, P.; Wissow, L.S. Impact of pet dog or cat exposure during childhood on mental illness during adolescence: a cohort study. *BMC Pediatr* **2022**, *22*, 572, doi:10.1186/s12887-022-03636-0.
188. Gadomski, A.M.; Scribani, M.B.; Krupa, N.; Jenkins, P. Pet dogs and child physical activity: the role of child-dog attachment. *Pediatr Obes* **2017**, *12*, e37-e40, doi:10.1111/ijpo.12156.
189. Gaillard, V.; Chastant, S.; England, G.; Forman, O.; German, A.J.; Suchodolski, J.S.; Villaverde, C.; Chavatte-Palmer, P.; Peron, F. Environmental risk factors in puppies and kittens for developing chronic disorders in adulthood: A call for research on developmental programming. *Front Vet Sci* **2022**, *9*, 944821, doi:10.3389/fvets.2022.944821.
190. Galambos, Á.; Gergely, A.; Kovács, A.B.; Kiss, O.; Topál, J. Affect matters: Positive and negative social stimulation influences dogs' behaviour in a subsequent situation involving an out-of-reach object. *Applied Animal Behaviour Science* **2021**, *236*, doi:10.1016/j.applanim.2021.105242.
191. Garrison, L.; Weiss, E. What do people want? Factors people consider when acquiring dogs, the complexity of the choices they make, and implications for nonhuman animal relocation programs. *J Appl Anim Welf Sci* **2015**, *18*, 57-73, doi:10.1080/10888705.2014.943836.
192. Gerencser, L.; Bunford, N.; Moesta, A.; Miklosi, A. Development and validation of the Canine Reward Responsiveness Scale - Examining individual differences in reward responsiveness of the domestic dog. *Sci Rep* **2018**, *8*, 4421, doi:10.1038/s41598-018-22605-1.
193. German, A.J. Style over substance: What can parenting styles tell us about ownership styles and obesity in companion animals? *Br J Nutr* **2015**, *113 Suppl*, S72-77, doi:10.1017/S0007114514002335.
194. Gertz, A.; Rabinowitz, P.M. Pet therapy: Helping patients cope. *American Family Physician* **2017**, *96*, 464.
195. Gillespie, K.; Lawson, V. 'My Dog is My Home': multispecies care and poverty politics in Los Angeles, California and Austin, Texas. *Gender, Place & Culture* **2017**, *24*, 774-793, doi:10.1080/0966369x.2017.1339021.

196. Giuffrida, M.A.; Brown, D.C.; Ellenberg, S.S.; Farrar, J.T. Development and psychometric testing of the canine owner-reported quality of life questionnaire, an instrument designed to measure quality of life in dogs with cancer. *Journal of the American Veterinary Medical Association* **2018**, *252*, 1073-1083, doi:10.2460/javma.252.9.1073.
197. Glenk, L.M.; Pribylova, L.; Stetina, B.U.; Demirel, S.; Weissenbacher, K. Perceptions on Health Benefits of Guide Dog Ownership in an Austrian Population of Blind People with and without a Guide Dog. *Animals (Basel)* **2019**, *9*, doi:10.3390/ani9070428.
198. Goldstein, E.; Burns-Nader, S.; Casper, D.; Parker, J. Exploring the implementation of child life services with facility dogs. *J Child Health Care* **2022**, 13674935221146382, doi:10.1177/13674935221146382.
199. Goryoka, G.W.; Cossaboom, C.M.; Gharpure, R.; Dawson, P.; Tansey, C.; Rossow, J.; Mrotz, V.; Rooney, J.; Torchetti, M.; Loiacono, C.M.; et al. One Health Investigation of SARS-CoV-2 Infection and Seropositivity among Pets in Households with Confirmed Human COVID-19 Cases-Utah and Wisconsin, 2020. *Viruses* **2021**, *13*, doi:10.3390/v13091813.
200. Greene, L.M.; Royal, K.D.; Bradley, J.M.; Lascelles, B.D.; Johnson, L.R.; Hawkins, E.C. Severity of Nasal Inflammatory Disease Questionnaire for Canine Idiopathic Rhinitis Control: Instrument Development and Initial Validity Evidence. *J Vet Intern Med* **2017**, *31*, 134-141, doi:10.1111/jvim.14629.
201. Grigg, E.K.; Chou, J.; Parker, E.; Gatesy-Davis, A.; Clarkson, S.T.; Hart, L.A. Stress-Related Behaviors in Companion Dogs Exposed to Common Household Noises, and Owners' Interpretations of Their Dogs' Behaviors. *Front Vet Sci* **2021**, *8*, 760845, doi:10.3389/fvets.2021.760845.
202. Gunter, L.; Protopopova, A.; Hooker, S.P.; Der Ananian, C.; Wynne, C.D.L. Impacts of Encouraging Dog Walking on Returns of Newly Adopted Dogs to a Shelter. *J Appl Anim Welf Sci* **2017**, *20*, 357-371, doi:10.1080/10888705.2017.1341318.
203. Gurry, G.A.; Campion, V.; Premawardena, C.; Woolley, I.; Shortt, J.; Bowden, D.K.; Kaplan, Z.; Dendle, C. High rates of potentially infectious exposures between immunocompromised patients and their companion animals: an unmet need for education. *Intern Med J* **2017**, *47*, 333-335, doi:10.1111/imj.13361.
204. Hall, N.J.; Otto, C.M.; Baltzer, W.I. Editorial: Working Dogs: Form and Function, Volume II. *Front Vet Sci* **2021**, *8*, 732304, doi:10.3389/fvets.2021.732304.
205. Hall, S.S.; Wright, H.F.; Mills, D.S. What Factors Are Associated with Positive Effects of Dog Ownership in Families with Children with Autism Spectrum Disorder? The Development of the Lincoln Autism Pet Dog Impact Scale. *PLoS One* **2016**, *11*, e0149736, doi:10.1371/journal.pone.0149736.
206. Hansen Wheat, C.; Larsson, L.; Berner, P.; Temrin, H. Human-directed attachment behavior in wolves suggests standing ancestral variation for human-dog attachment bonds. *Ecol Evol* **2022**, *12*, e9299, doi:10.1002/ece3.9299.
207. Hardie, S.; Mai, D.L.; Howell, T.J. Social Support and Wellbeing in Cat and Dog Owners, and the Moderating Influence of Pet–Owner Relationship Quality. *Anthrozoös* **2023**, *36*, 891-907, doi:10.1080/08927936.2023.2182029.
208. Harris, C.R.; Prouvost, C. Jealousy in dogs. *PLoS One* **2014**, *9*, e94597, doi:10.1371/journal.pone.0094597.
209. Harris, L.K. Dog Theft: A Case for Tougher Sentencing Legislation. *Animals (Basel)* **2018**, *8*, doi:10.3390/ani8050078.
210. Hartnack, S.; Alobo, G.; Kankya, C. Toxocariasis in Africa: A One Health perspective. *Travel Med Infect Dis* **2017**, *20*, 3-4, doi:10.1016/j.tmaid.2017.11.001.
211. Harvie, H.; Rodrigo, A.; Briggs, C.; Thiessen, S.; Kelly, D.M. Does stress run through the leash? An examination of stress transmission between owners and dogs during a walk. *Anim Cogn* **2021**, *24*, 239-250, doi:10.1007/s10071-020-01460-6.
212. Harwood, C.; Kaczmarek, E.; Drake, D. Parental Perceptions of the Nature of the Relationship Children with Autism Spectrum Disorders Share with Their Canine Companion. *J Autism Dev Disord* **2019**, *49*, 248-259, doi:10.1007/s10803-018-3759-7.
213. Hasoon, B.C.; Shipp, A.E.; Hasoon, J. A look at the incidence and risk factors for dog bites in unincorporated Harris County, Texas, USA. *Vet World* **2020**, *13*, 419-425, doi:10.14202/vetworld.2020.419-425.
214. Heath, S.; Wilson, C. Canine and feline enrichment in the home and kennel: a guide for practitioners. *Vet Clin North Am Small Anim Pract* **2014**, *44*, 427-449, doi:10.1016/j.cvsm.2014.01.003.
215. Heberlein, M.T.; Turner, D.C.; Manser, M.B. Dogs' (Canis familiaris) attention to human perception: Influence of breed groups and life experiences. *J Comp Psychol* **2017**, *131*, 19-29, doi:10.1037/com0000050.
216. Heberlein, M.T.E.; Manser, M.B.; Turner, D.C. Deceptive-like behaviour in dogs (Canis familiaris). *Anim Cogn* **2017**, *20*, 511-520, doi:10.1007/s10071-017-1078-6.
217. Hecht, E.E.; Smaers, J.B.; Dunn, W.D.; Kent, M.; Preuss, T.M.; Gutman, D.A. Significant Neuroanatomical Variation Among Domestic Dog Breeds. *J Neurosci* **2019**, *39*, 7748-7758, doi:10.1523/JNEUROSCI.0303-19.2019.
218. Henschel, M.; Winters, J.; Muller, T.F.; Brauer, J. Effect of shared information and owner behavior on showing in dogs (Canis familiaris). *Anim Cogn* **2020**, *23*, 1019-1034, doi:10.1007/s10071-020-01409-9.
219. Herbeck, Y.E.; Gulevich, R.G. Neuropeptides as facilitators of domestication. *Cell Tissue Res* **2019**, *375*, 295-307, doi:10.1007/s00441-018-2939-2.
220. Herbeck, Y.E.; Gulevich, R.G.; Shepeleva, D.V.; Grinevich, V.V. Oxytocin: Coevolution of human and domesticated animals. *Russian Journal of Genetics: Applied Research* **2017**, *7*, 235-242, doi:10.1134/s2079059717030042.
221. Herron, M.E.; Shreyer, T. The pet-friendly veterinary practice: a guide for practitioners. *Vet Clin North Am Small Anim Pract* **2014**, *44*, 451-481, doi:10.1016/j.cvsm.2014.01.010.
222. Herwijnen, I.R.V.; Corbee, R.J.; Endenburg, N.; Beerda, B.; Borg, J. Permissive parenting of the dog associates with dog overweight in a survey among 2,303 Dutch dog owners. *PLoS One* **2020**, *15*, e0237429, doi:10.1371/journal.pone.0237429.
223. Herwijnen, I.R.v.; van der Borg, J.; Naguib, M.; Beerda, B. Rein sensor leash tension measurements in owner-dog dyads navigating a course with distractions. *Journal of Veterinary Behavior* **2020**, *35*, 45-46, doi:10.1016/j.jveb.2019.08.006.

224. Herwijnen, I.R.V.; van der Borg, J.A.M.; Naguib, M.; Beerda, B. Dog ownership satisfaction determinants in the owner-dog relationship and the dog's behaviour. *PLoS One* **2018**, *13*, e0204592, doi:10.1371/journal.pone.0204592.
225. Herzog, R.W.; Nichols, T.C.; Su, J.; Zhang, B.; Sherman, A.; Merricks, E.P.; Raymer, R.; Perrin, G.Q.; Hager, M.; Wiinberg, B.; et al. Oral Tolerance Induction in Hemophilia B Dogs Fed with Transplastomic Lettuce. *Mol Ther* **2017**, *25*, 512-522, doi:10.1016/j.ymthe.2016.11.009.
226. Hesselmar, B.; Hicke-Roberts, A.; Lundell, A.C.; Adlerberth, I.; Rudin, A.; Saalman, R.; Wennergren, G.; Wold, A.E. Pet-keeping in early life reduces the risk of allergy in a dose-dependent fashion. *PLoS One* **2018**, *13*, e0208472, doi:10.1371/journal.pone.0208472.
227. Heuberger, R.; Petty, M.; Huntingford, J. Companion Animal Owner Perceptions, Knowledge, and Beliefs Regarding Pain Management in End-of-Life Care. *Top Companion Anim Med* **2016**, *31*, 152-159, doi:10.1053/j.tcam.2017.02.001.
228. Hicks, J.R.; Weisman, C.J. Work or play?: An exploration of the relationships between people and their service dogs in leisure activities. *Journal of Leisure Research* **2015**, *47*, 243-262, doi:10.1080/00222216.2015.11950359.
229. Hladky-Krage, B.; Hoffman, C.L. Expectations versus Reality of Designer Dog Ownership in the United States. *Animals (Basel)* **2022**, *12*, doi:10.3390/ani12233247.
230. Hoffman, J.M.; Tolbert, M.K.; Promislow, D.E.L.; Dog Aging Project, C. Demographic factors associated with joint supplement use in dogs from the Dog Aging Project. *Front Vet Sci* **2022**, *9*, 906521, doi:10.3389/fvets.2022.906521.
231. Hofman, C.; Rick, T. The Dogs of CA-SRI-2: Osteometry of Canis familiaris from Santa Rosa Island, California. *Ethnobiology Letters* **2014**, *5*, doi:10.14237/ebl.5.2014.144.
232. Hoglin, A.; Van Poucke, E.; Katajamaa, R.; Jensen, P.; Theodorsson, E.; Roth, L.S.V. Long-term stress in dogs is related to the human-dog relationship and personality traits. *Sci Rep* **2021**, *11*, 8612, doi:10.1038/s41598-021-88201-y.
233. Holder, T.R.N.; Gruen, M.E.; Roberts, D.L.; Somers, T.; Bozkurt, A. A Systematic Literature Review of Animal-Assisted Interventions in Oncology (Part II): Theoretical Mechanisms and Frameworks. *Integr Cancer Ther* **2020**, *19*, 1534735420943269, doi:10.1177/1534735420943269.
234. Holland, K.E.; Owczarczak-Garstecka, S.C.; Anderson, K.L.; Casey, R.A.; Christley, R.M.; Harris, L.; McMillan, K.M.; Mead, R.; Murray, J.K.; Samet, L.; et al. "More Attention than Usual": A Thematic Analysis of Dog Ownership Experiences in the UK during the First COVID-19 Lockdown. *Animals (Basel)* **2021**, *11*, doi:10.3390/ani11010240.
235. Holttum, S. Pets, animal-assisted therapy and social inclusion. *Mental Health and Social Inclusion* **2018**, *22*, 65-71, doi:10.1108/mhsi-02-2018-0004.
236. Horowitz, A.; Hecht, J. Examining dog-human play: the characteristics, affect, and vocalizations of a unique interspecific interaction. *Anim Cogn* **2016**, *19*, 779-788, doi:10.1007/s10071-016-0976-3.
237. Horton, L.; Griffen, M.; Chang, L.; Newcomb, A.B. Efficacy of Animal-Assisted Therapy in Treatment of Patients With Traumatic Brain Injury: A Randomized Trial. *J Trauma Nurs* **2023**, *30*, 68-74, doi:10.1097/JTN.0000000000000705.
238. Hritcu, L.D.; Horhogea, C.; Ciobica, A.; Spataru, M.C.; Spataru, C.; Kis, A. Conceptual replication of canine serum oxytocin increase following a positive dog-human interaction. *Revista de Chimie* **2019**, *70*, 1579-1581, doi:10.37358/rc.19.5.7172.
239. Huber, L.; Salobir, K.; Mundry, R.; Cimarelli, G. Selective overimitation in dogs. *Learn Behav* **2020**, *48*, 113-123, doi:10.3758/s13420-019-00400-w.
240. Humby, L.; Barclay, E. Pawsitive Solutions: An Overview of Prison Dog Programs in Australia. *The Prison Journal* **2018**, *98*, 580-603, doi:10.1177/0032885518793951.
241. Hunt, R.L.; England, G.C.W.; Asher, L.; Whiteside, H.; Harvey, N.D. Concurrent and Predictive Criterion Validity of a Puppy Behaviour Questionnaire for Predicting Training Outcome in Juvenile Guide Dogs. *Animals (Basel)* **2020**, *10*, doi:10.3390/ani10122382.
242. Hupfeld, J.; Dolle, M.; Volk, H.; Rieder, J. Effect of long-term management of hypoadrenocorticism on the quality of life of affected dogs and their owners. *Vet Rec* **2022**, *191*, e1977, doi:10.1002/vetr.1977.
243. Ingram, K.M.; Cohen-Filipic, J. Benefits, challenges, and needs of people living with cancer and their companion dogs: An exploratory study. *J Psychosoc Oncol* **2019**, *37*, 110-126, doi:10.1080/07347332.2018.1529010.
244. Irvin, S. The healing role of assistance dogs: What these partnerships tell us about the human–animal bond. *Animal Frontiers* **2014**, *4*, 66-71, doi:10.2527/af.2014-0024.
245. Isgate, S.; Couchman, J.J. What Makes a Dog Adoptable? An Eye-Tracking Investigation. *J Appl Anim Welf Sci* **2018**, *21*, 69-81, doi:10.1080/10888705.2017.1374865.
246. Jacobs, J.A.; Coe, J.B.; Pearl, D.L.; Widowski, T.M.; Niel, L. Factors associated with canine resource guarding behaviour in the presence of people: A cross-sectional survey of dog owners. *Prev Vet Med* **2018**, *161*, 143-153, doi:10.1016/j.prevetmed.2017.02.005.
247. Jalongo, M.R. An Attachment Perspective on the Child–Dog Bond: Interdisciplinary and International Research Findings. *Early Childhood Education Journal* **2015**, *43*, 395-405, doi:10.1007/s10643-015-0687-4.
248. Jalongo, M.R. Introduction: Building a Rationale for Prison Dog Programs. In *Prison Dog Programs*; 2019; pp. 1-16.
249. Jalongo, M.R. Making It Real: Neuroscience and the Narrative Mode in Prison Dog Programs. In *Prison Dog Programs*; 2019; pp. 255-274.
250. Janevic, M.R.; Shute, V.; Connell, C.M.; Piette, J.D.; Goesling, J.; Fynke, J. The Role of Pets in Supporting Cognitive-Behavioral Chronic Pain Self-Management: Perspectives of Older Adults. *J Appl Gerontol* **2020**, *39*, 1088-1096, doi:10.1177/0733464819856270.

251. Janssens, L.; Giemsch, L.; Schmitz, R.; Street, M.; Van Dongen, S.; Crombé, P. A new look at an old dog: Bonn-Oberkassel reconsidered. *Journal of Archaeological Science* **2018**, *92*, 126-138, doi:10.1016/j.jas.2018.01.004.
252. Janssens, M.; Eshuis, J.; Peeters, S.; Lataster, J.; Reijnders, J.; Enders-Slegers, M.-J.; Jacobs, N. The Pet-Effect in Daily Life: An Experience Sampling Study on Emotional Wellbeing in Pet Owners. *Anthrozoös* **2020**, *33*, 579-588, doi:10.1080/08927936.2020.1771061.
253. Janssens, M.; Janssens, E.; Eshuis, J.; Lataster, J.; Simons, M.; Reijnders, J.; Jacobs, N. Companion Animals as Buffer against the Impact of Stress on Affect: An Experience Sampling Study. *Animals (Basel)* **2021**, *11*, doi:10.3390/ani11082171.
254. Jarolmen, J.; Patel, G. The Effects of Animal-Assisted Activities on College Students Before and After a Final Exam. *Journal of Creativity in Mental Health* **2018**, *13*, 264-274, doi:10.1080/15401383.2018.1425941.
255. Jayawardene, W.; Huber, L.; McDonnell, J.; Curran, L.; Larson, S.; Dickinson, S.; Chen, X.; Pena, E.; Carson, A.; Johnston, J. 'Tracking Together'-Simultaneous Use of Human and Dog Activity Trackers: Protocol for a Factorial, Randomized Controlled Pilot Trial. *Int J Environ Res Public Health* **2021**, *18*, doi:10.3390/ijerph18041561.
256. Jensen, C.L.; Rodriguez, K.E.; MacLean, E.L.; Abdul Wahab, A.H.; Sabbaghi, A.; O'Haire, M.E. Characterizing veteran and PTSD service dog teams: Exploring potential mechanisms of symptom change and canine predictors of efficacy. *PLoS One* **2022**, *17*, e0269186, doi:10.1371/journal.pone.0269186.
257. Jim, H.L.; Marshall-Pescini, S.; Range, F. Do dogs eavesdrop on human interactions in a helping situation? *PLoS One* **2020**, *15*, e0237373, doi:10.1371/journal.pone.0237373.
258. Johnson, H. Informed clients benefit practices and pets. *Vet Rec* **2017**, *180*, i-ii, doi:10.1136/vr.j2022.
259. Jokinen, T.S. Compliance in canine epilepsy: can the owner be the cause of treatment failure? *Vet Rec* **2021**, *188*, 148-150, doi:10.1002/vetr.224.
260. Joosten, P.; Van Cleven, A.; Sarrazin, S.; Paepe, D.; De Sutter, A.; Dewulf, J. Dogs and Their Owners Have Frequent and Intensive Contact. *Int J Environ Res Public Health* **2020**, *17*, doi:10.3390/ijerph17124300.
261. Joseph, N.; Chandramohan, A.K.; Lorainne D'souza, A.; Shekar C, B.; Hariram, S.; Nayak, A.H. Assessment of pet attachment and its relationship with stress and social support among residents in Mangalore city of south India. *Journal of Veterinary Behavior* **2019**, *34*, 1-6, doi:10.1016/j.jveb.2019.06.009.
262. Julien, D.A.; Sargeant, J.M.; Filejski, C.; Harper, S.L. Ouch! A cross-sectional study investigating self-reported human exposure to dog bites in rural and urban households in southern Ontario, Canada. *Zoonoses Public Health* **2020**, *67*, 554-565, doi:10.1111/zph.12719.
263. Jung, C.; Pörtl, D. Scavenging hypothesis: Lack of evidence for dog domestication on the waste dump. *Dog Behavior* **2018**, *4*, 41-56, doi:10.4454/db.v4i2.73.
264. Kabel, A.; Khosla, N.; Teti, M. The Dog narratives: Benefits of the human-animal bond for women with HIV. *J HIV AIDS Soc Serv* **2015**, *14*, 405-416, doi:10.1080/15381501.2013.860069.
265. Kamel, M.S.; El-Sayed, A.A.; Munds, R.A.; Verma, M.S. Interactions between Humans and Dogs during the COVID-19 Pandemic: Recent Updates and Future Perspectives. *Animals (Basel)* **2023**, *13*, doi:10.3390/ani13030524.
266. Kaminski, J.; Waller, B.M.; Diogo, R.; Hartstone-Rose, A.; Burrows, A.M. Evolution of facial muscle anatomy in dogs. *Proc Natl Acad Sci U S A* **2019**, *116*, 14677-14681, doi:10.1073/pnas.1820653116.
267. Kantarakia, C.; Tsoumani, M.E.; Galanos, A.; Mathioudakis, A.G.; Giannoulaki, E.; Beloukas, A.; Voyiatzaki, C. Comparison of the Level of Awareness about the Transmission of Echinococcosis and Toxocariasis between Pet Owners and Non-Pet Owners in Greece. *Int J Environ Res Public Health* **2020**, *17*, doi:10.3390/ijerph17155292.
268. Karkdijk, E.M.; Duindam, H.M.; Dekovic, M.; Creemers, H.E.; Asscher, J.J. A Friend in Prison: Human-Animal Bond, Stress and Self-Esteem of Detained Juveniles in Dutch Cell Dogs. *Animals (Basel)* **2022**, *12*, doi:10.3390/ani12050646.
269. Karl, S.; Anderle, K.; Volter, C.J.; Viranyi, Z. Pet dogs' Behavioural Reaction to Their Caregiver's Interactions with a Third Party: Join in or Interrupt? *Animals (Basel)* **2022**, *12*, doi:10.3390/ani12121574.
270. Karl, S.; Boch, M.; Zamansky, A.; van der Linden, D.; Wagner, I.C.; Volter, C.J.; Lamm, C.; Huber, L. Exploring the dog-human relationship by combining fMRI, eye-tracking and behavioural measures. *Sci Rep* **2020**, *10*, 22273, doi:10.1038/s41598-020-79247-5.
271. Kekecs, Z.; Szollosi, A.; Palfi, B.; Szaszi, B.; Kovacs, K.J.; Dienes, Z.; Aczel, B. Commentary: Oxytocin-gaze positive loop and the coevolution of human-dog bonds. *Front Neurosci* **2016**, *10*, 155, doi:10.3389/fnins.2016.00155.
272. Kent, J.L.; Mulley, C. Riding with dogs in cars: What can it teach us about transport practices and policy? *Transportation Research Part A: Policy and Practice* **2017**, *106*, 278-287, doi:10.1016/j.tra.2017.09.014.
273. Khorozyan, I.; Soofi, M.; Soufi, M.; Hamidi, A.K.; Ghoddousi, A.; Waltert, M. Effects of shepherds and dogs on livestock depredation by leopards (Panthera pardus) in north-eastern Iran. *PeerJ* **2017**, *5*, e3049, doi:10.7717/peerj.3049.
274. Kiflu, B.; Alemayehu, H.; Abdurahaman, M.; Negash, Y.; Eguale, T. Salmonella serotypes and their antimicrobial susceptibility in apparently healthy dogs in Addis Ababa, Ethiopia. *BMC Vet Res* **2017**, *13*, 134, doi:10.1186/s12917-017-1055-y.
275. Kim, W.H.; Min, K.D.; Cho, S.I.; Cho, S. The Relationship Between Dog-Related Factors and Owners' Attitudes Toward Pets: An Exploratory Cross-Sectional Study in Korea. *Front Vet Sci* **2020**, *7*, 493, doi:10.3389/fvets.2020.00493.
276. Kin, C. Psychiatric nurses◎views on caring: Patients and canine companions. *Journal of Psychosocial Nursing and Mental Health Services* **2017**, *55*, 46-52, doi:10.3928/02793695-20170301-06.
277. King, B.; McGlinn, M.; Duberstein, A. Mutuality and well-being: The human–animal bond. *The Humanistic Psychologist* **2023**, *51*, 133-141, doi:10.1037/hum0000257.

278. Kis, A.; Ciobica, A.; Topal, J. The effect of oxytocin on human-directed social behaviour in dogs (Canis familiaris). *Horm Behav* **2017**, *94*, 40-52, doi:10.1016/j.yhbeh.2017.06.001.
279. Kiss, O.; Kovacs, K.; Szantho, F.; Topal, J. Similarity between an unfamiliar human and the owner affects dogs' preference for human partner when responding to an unsolvable problem. *Learn Behav* **2018**, *46*, 430-441, doi:10.3758/s13420-018-0337-y.
280. Kogan, L.; Hellyer, P.; Duncan, C.; Schoenfeld-Tacher, R. A pilot investigation of the physical and psychological benefits of playing Pokémon GO for dog owners. *Computers in Human Behavior* **2017**, *76*, 431-437, doi:10.1016/j.chb.2017.07.043.
281. Kogan, L.R.; Bussolari, C. Exploring the Potential Impact of a Virtual Body Scan Meditation Exercise Conducted With Pet Dogs on Recipients and Facilitators. *Front Psychol* **2021**, *12*, 698075, doi:10.3389/fpsyg.2021.698075.
282. Kogan, L.R.; Bussolari, C.; Currin-McCulloch, J.; Packman, W.; Erdman, P. Disenfranchised Guilt-Pet Owners' Burden. *Animals (Basel)* **2022**, *12*, doi:10.3390/ani12131690.
283. Kogan, L.R.; Erdman, P.; Bussolari, C.; Currin-McCulloch, J.; Packman, W. The Initial Months of COVID-19: Dog Owners' Veterinary-Related Concerns. *Front Vet Sci* **2021**, *8*, 629121, doi:10.3389/fvets.2021.629121.
284. Kogan, L.R.; Wallace, J.E.; Hellyer, P.W.; Carr, E.C.J. Canine Caregivers: Paradoxical Challenges and Rewards. *Animals (Basel)* **2022**, *12*, doi:10.3390/ani12091074.
285. Kolm, N.; Temrin, H.; Miklosi, A.; Kubinyi, E.; Garamszegi, L.Z. The link between selection for function and human-directed play behaviour in dogs. *Biol Lett* **2020**, *16*, 20200366, doi:10.1098/rsbl.2020.0366.
286. Kotnik, T. Quality of Life of Allergic Dogs Treated with Allergen-Specific Immunotherapy-A Retrospective Study. *Vet Sci* **2023**, *10*, doi:10.3390/vetsci10020072.
287. Koyasu, H.; Kikusui, T.; Takagi, S.; Nagasawa, M. The Gaze Communications Between Dogs/Cats and Humans: Recent Research Review and Future Directions. *Front Psychol* **2020**, *11*, 613512, doi:10.3389/fpsyg.2020.613512.
288. Kramer, C.K.; Mehmood, S.; Suen, R.S. Dog Ownership and Survival: A Systematic Review and Meta-Analysis. *Circ Cardiovasc Qual Outcomes* **2019**, *12*, e005554, doi:10.1161/CIRCOUTCOMES.119.005554.
289. Krause-Parello, C.A.; Thames, M.; Ray, C.M.; Kolassa, J. Examining the Effects of a Service-Trained Facility Dog on Stress in Children Undergoing Forensic Interview for Allegations of Child Sexual Abuse. *J Child Sex Abus* **2018**, *27*, 305-320, doi:10.1080/10538712.2018.1443303.
290. Kroger, E.; Slettebo, A.; Fossum, M. Agility activities for children in a municipality in Norway. *J Community Health Nurs* **2015**, *32*, 53-67, doi:10.1080/07370016.2015.992265.
291. Krouzecky, C.; Aden, J.; Hametner, K.; Klaps, A.; Kovacovsky, Z.; Stetina, B.U. Fantastic Beasts and Why It Is Necessary to Understand Our Relationship-Animal Companionship under Challenging Circumstances Using the Example of Long-Covid. *Animals (Basel)* **2022**, *12*, doi:10.3390/ani12151892.
292. Krouzecky, C.; Emmett, L.; Klaps, A.; Aden, J.; Bunina, A.; Stetina, B.U. And in the Middle of My Chaos There Was You?-Dog Companionship and Its Impact on the Assessment of Stressful Situations. *Int J Environ Res Public Health* **2019**, *16*, doi:10.3390/ijerph16193664.
293. Krueger, F.; Mitchell, K.C.; Deshpande, G.; Katz, J.S. Human-dog relationships as a working framework for exploring human-robot attachment: a multidisciplinary review. *Anim Cogn* **2021**, *24*, 371-385, doi:10.1007/s10071-021-01472-w.
294. Kwaghe, A.V.; Okomah, D.; Okoli, I.; Kachalla, M.G.; Aligana, M.; Alabi, O.; Mshelbwala, G.M. Estimation of dog population in Nasarawa state Nigeria: a pilot study. *Pan Afr Med J* **2019**, *34*, 25, doi:10.11604/pamj.2019.34.25.16755.
295. Lagutchik, M.; Baker, J.; Balser, J.; Burghardt, W.; Enroth, M.; Flournoy, S.; Giles, J.; Grimm, P.; Hiniker, J.; Johnson, J.; et al. Trauma Management of Military Working Dogs. *Mil Med* **2018**, *183*, 180-189, doi:10.1093/milmed/usy119.
296. Lamontagne, A.; Legou, T.; Rauchbauer, B.; Grosbras, M.H.; Fabre, F.; Gaunet, F. Behavioural synchronization and social referencing of dogs and humans: walking in dyad vs in group. *Anim Cogn* **2023**, *26*, 1021-1034, doi:10.1007/s10071-023-01750-9.
297. Lass-Hennemann, J.; Schafer, S.K.; Sopp, M.R.; Michael, T. The relationship between attachment to pets and mental health: the shared link via attachment to humans. *BMC Psychiatry* **2022**, *22*, 586, doi:10.1186/s12888-022-04199-1.
298. Lavan, R.P.; Tunceli, K.; Zhang, D.; Normile, D.; Armstrong, R. Assessment of dog owner adherence to veterinarians' flea and tick prevention recommendations in the United States using a cross-sectional survey. *Parasit Vectors* **2017**, *10*, 284, doi:10.1186/s13071-017-2217-2.
299. Lawson, S.; Kirman, B.; Linehan, C.; Feltwell, T.; Hopkins, L. Problematising Upstream Technology through Speculative Design. In Proceedings of the Proceedings of the 33rd Annual ACM Conference on Human Factors in Computing Systems, 2015; pp. 2663-2672.
300. Lecova, L.; Hammerbauerova, I.; Tumova, P.; Nohynkova, E. Companion animals as a potential source of Giardia intestinalis infection in humans in the Czech Republic - A pilot study. *Vet Parasitol Reg Stud Reports* **2020**, *21*, 100431, doi:10.1016/j.vprsr.2020.100431.
301. Lee, S.; Wendland, T.M.; Rao, S.; Magee, C. Orthotic Device Use in Canine Patients: Owner Perception of Quality of Life for Owners and Patients. *Front Vet Sci* **2021**, *8*, 709364, doi:10.3389/fvets.2021.709364.
302. Leighton, S.C.; Nieforth, L.O.; O'Haire, M.E. Assistance dogs for military veterans with PTSD: A systematic review, meta-analysis, and meta-synthesis. *PLoS One* **2022**, *17*, e0274960, doi:10.1371/journal.pone.0274960.
303. Lenkei, R.; Farago, T.; Bakos, V.; Pongracz, P. Separation-related behavior of dogs shows association with their reactions to everyday situations that may elicit frustration or fear. *Sci Rep* **2021**, *11*, 19207, doi:10.1038/s41598-021-98526-3.
304. Lesch, R.; Kotrschal, K.; Schoberl, I.; Beetz, A.; Solomon, J.; Fitch, W.T. Talking to Dogs: Companion Animal-Directed Speech in a Stress Test. *Animals (Basel)* **2019**, *9*, doi:10.3390/ani9070417.

305. Letissier, G. From Dog Alterity to Canine Sublime: A Cross-Century Reading of Victorian Fiction. *Cahiers victoriens et édouardiens* **2017**, doi:10.4000/cve.3224.
306. Li, K.; Kou, J.; Lam, Y.; Lyons, P.; Nguyen, S. First-Time Experience in Owning a Dog Guide by Older Adults with Vision Loss. *Journal of Visual Impairment & Blindness* **2019**, *113*, 452-463, doi:10.1177/0145482x19868351.
307. Linder, D.; Mueller, M. Pet obesity management: beyond nutrition. *Vet Clin North Am Small Anim Pract* **2014**, *44*, 789-806, vii, doi:10.1016/j.cvsm.2014.03.004.
308. Linder, D.E.; Santiago, S.; Halbreich, E.D. Is There a Correlation Between Dog Obesity and Human Obesity? Preliminary Findings of Overweight Status Among Dog Owners and Their Dogs. *Front Vet Sci* **2021**, *8*, 654617, doi:10.3389/fvets.2021.654617.
309. Lindsay, S.; Thiyagarajah, K. The impact of service dogs on children, youth and their families: A systematic review. *Disabil Health J* **2021**, *14*, 101012, doi:10.1016/j.dhjo.2020.101012.
310. Lippi, G.; Cervellin, G.; Dondi, M.; Targher, G. Hypoglycemia alert dogs: A novel, cost-effective approach for diabetes monitoring? *Alternative Therapies in Health and Medicine* **2016**, *22*, 14-18.
311. Lloyd, J.; Budge, C.; La Grow, S.; Stafford, K. The End of the Partnership With a Guide Dog: Emotional Responses, Effects on Quality of Life and Relationships With Subsequent Dogs. *Front Vet Sci* **2021**, *8*, 543463, doi:10.3389/fvets.2021.543463.
312. Lloyd, J.; Budge, C.; Stafford, K. Handlers' Expectations and Perceived Compatibility regarding the Partnership with Their First Guide Dogs. *Animals (Basel)* **2021**, *11*, doi:10.3390/ani11102765.
313. Lloyd, J.; Johnston, L.; Lewis, J. Psychiatric Assistance Dog Use for People Living With Mental Health Disorders. *Front Vet Sci* **2019**, *6*, 166, doi:10.3389/fvets.2019.00166.
314. Lord, K.; Feinstein, M.; Smith, B.; Coppinger, R. Variation in reproductive traits of members of the genus Canis with special attention to the domestic dog (Canis familiaris). *Behav Processes* **2013**, *92*, 131-142, doi:10.1016/j.beproc.2012.10.009.
315. Lord, M.; Casey, R.A.; Loftus, B.A.; Blackwell, E.J. Risk factors for human-directed canine aggression in a referral level clinical population. *Vet Rec* **2017**, *181*, 44, doi:10.1136/vr.103638.
316. Luisana, E.; Saker, K.; Jaykus, L.A.; Getty, C. Survey evaluation of dog owners' feeding practices and dog bowls' hygiene assessment in domestic settings. *PLoS One* **2022**, *17*, e0259478, doi:10.1371/journal.pone.0259478.
317. Luna-Cortes, G. The Influence of Materialism on Purebred Dogs' Welfare Among Two Different Generations in Colombia (South America). *J Appl Anim Welf Sci* **2019**, *22*, 149-158, doi:10.1080/10888705.2018.1452019.
318. Luno, I.; Muniesa, A.; Palacio, J.; Garcia-Belenguer, S.; Rosado, B. Detection of owner-perceived emotional eating in companion dogs: A regression modelling approach. *Vet Rec* **2021**, *189*, e63, doi:10.1002/vetr.63.
319. Machiela, M.J.; Chanock, S.J. GWAS is going to the dogs. *Genome Biology* **2014**, *15*, doi:10.1186/gb4166.
320. Maharaj, N.; Haney, C.J. A Qualitative Investigation of the Significance of Companion Dogs. *West J Nurs Res* **2015**, *37*, 1175-1193, doi:10.1177/0193945914545176.
321. Maharaj, N.; Kazanjian, A.; Haney, C.J. The Human–Canine Bond: A Sacred Relationship. *Journal of Spirituality in Mental Health* **2016**, *18*, 76-89, doi:10.1080/19349637.2015.1047922.
322. Mai, D.L.; Howell, T.; Benton, P.; Bennett, P.C. Beyond puppy selection—considering the role of puppy raisers in bringing out the best in assistance dog puppies. *Journal of Veterinary Behavior* **2021**, *42*, 1-10, doi:10.1016/j.jveb.2020.11.002.
323. Mantini, C.; Capparuccia, C.; Cademartiri, F.; Messalli, G.; Mastrodicasa, D.; Cinalli, S.; Cotroneo, A.R.; Caputo, M. Uncommon Isolated Unilocular Myocardial Cyst in a Dog-Friendly Young Female Patient - Multimodality Imaging. *Circ J* **2017**, *81*, 1056-1058, doi:10.1253/circj.CJ-16-1215.
324. Marchetti, V.; Gori, E.; Mariotti, V.; Gazzano, A.; Mariti, C. The Impact of Chronic Inflammatory Enteropathy on Dogs' Quality of Life and Dog-Owner Relationship. *Vet Sci* **2021**, *8*, doi:10.3390/vetsci8080166.
325. Mariti, C.; Lenzini, L.; Carlone, B.; Zilocchi, M.; Ogi, A.; Gazzano, A. Does attachment to man already exist in 2 months old normally raised dog puppies? A pilot study. *Dog Behavior* **2020**, *6*, 1-11, doi:10.4454/db.v6i1.96.
326. Mariti, C.; Ricci, E.; Zilocchi, M.; Gazzano, A. Owners as a secure base for their dogs. *Behaviour* **2013**, *150*, 1275-1294, doi:10.1163/1568539x-00003095.
327. Marks, G.; McVilly, K.R. Assistance Dogs for People with Younger (Early)-Onset Dementia: The Family Carer's Experience. *Animals (Basel)* **2023**, *13*, doi:10.3390/ani13050777.
328. Marshall-Pescini, S.; Schaebs, F.S.; Gaugg, A.; Meinert, A.; Deschner, T.; Range, F. The Role of Oxytocin in the Dog-Owner Relationship. *Animals (Basel)* **2019**, *9*, doi:10.3390/ani9100792.
329. Martellucci, S.; Belvisi, V.; Ralli, M.; Stadio, A.D.; Musacchio, A.; Greco, A.; Gallo, A.; Vincentiis, M.; Attanasio, G. Assistance Dogs for Persons with Hearing Impairment: A Review. *Int Tinnitus J* **2019**, *23*, 26-30, doi:10.5935/0946-5448.20190005.
330. Martin, A.L.; Kelling, A.S.; Mallavarapu, S. Attitudes Toward Dog Relinquishment as Assessed Through a Survey of University Students. *Anthrozoös* **2021**, *34*, 201-215, doi:10.1080/08927936.2021.1885142.
331. Martin, N.D.; Pascual, J.L.; Hirsch, J.; Holena, D.N.; Kaplan, L.J. Excluded but not forgotten: Veterinary emergency care during emergencies and disasters. *Am J Disaster Med* **2020**, *15*, 25-31, doi:10.5055/ajdm.2020.0352.
332. Martos Martinez-Caja, A.; De Herdt, V.; Boon, P.; Brandl, U.; Cock, H.; Parra, J.; Perucca, E.; Thadani, V.; Moons, C.P.H. Seizure-alerting behavior in dogs owned by people experiencing seizures. *Epilepsy Behav* **2019**, *94*, 104-111, doi:10.1016/j.yebeh.2019.02.001.
333. Martos Martinez-Caja, A.; De Herdt, V.; Enders-Slegers, M.J.; Moons, C.P.H. Pet ownership, feelings of loneliness, and mood in people affected by the first COVID-19 lockdown. *J Vet Behav* **2022**, *57*, 52-63, doi:10.1016/j.jveb.2022.09.008.

334. Marx, A.; Lenkei, R.; Perez Fraga, P.; Bakos, V.; Kubinyi, E.; Farago, T. Occurrences of non-linear phenomena and vocal harshness in dog whines as indicators of stress and ageing. *Sci Rep* **2021**, *11*, 4468, doi:10.1038/s41598-021-83614-1.
335. Masson, S.; Gaultier, E. Retrospective study on hypersensitivity-hyperactivity syndrome in dogs: Long-term outcome of high dose fluoxetine treatment and proposal of a clinical score. *Dog Behavior* **2018**, *4*, 15-32, doi:10.4454/db.v4i2.79.
336. Mauti, S.; Léchenne, M.; Mbilo, C.; Nel, L.; Zinsstag, J. Rabies. In *Transboundary Animal Diseases in Sahelian Africa and Connected Regions*; 2019; pp. 107-119.
337. Mauti, S.; Traore, A.; Sery, A.; Bryssinckx, W.; Hattendorf, J.; Zinsstag, J. First study on domestic dog ecology, demographic structure and dynamics in Bamako, Mali. *Prev Vet Med* **2017**, *146*, 44-51, doi:10.1016/j.prevetmed.2017.07.009.
338. Mayers, R. Dogs Unleashed: The Positive Role Dogs Play during COVID-19. *Leisure Sciences* **2020**, *43*, 252-259, doi:10.1080/01490400.2020.1774010.
339. McCool, K.E.; Kedrowicz, A.A. Evaluation of Veterinary Students◎Communication Skills with a Service Dog Handler in a Simulated Client Scenario. *Journal of Veterinary Medical Education* **2021**, *48*, 538-548, doi:10.3138/jvme.2019-0140.
340. McGetrick, J.; Poncet, L.; Amann, M.; Schullern-Schrattenhofen, J.; Fux, L.; Martinez, M.; Range, F. Dogs fail to reciprocate the receipt of food from a human in a food-giving task. *PLoS One* **2021**, *16*, e0253277, doi:10.1371/journal.pone.0253277.
341. McHugh, S. Bitch, Bitch, Bitch: Personal Criticism, Feminist Theory, and Dog-writing. *Hypatia* **2020**, *27*, 616-635, doi:10.1111/j.1527-2001.2012.01289.x.
342. Merkouri, A.; Graham, T.M.; O'Haire, M.E.; Purewal, R.; Westgarth, C. Dogs and the Good Life: A Cross-Sectional Study of the Association Between the Dog-Owner Relationship and Owner Mental Wellbeing. *Front Psychol* **2022**, *13*, 903647, doi:10.3389/fpsyg.2022.903647.
343. Merola, I.; Marshall-Pescini, S.; D◎Aniello, B.; Prato-Previde, E. Social referencing: Water rescue trained dogs are less affected than pet dogs by the stranger's message. *Applied Animal Behaviour Science* **2013**, *147*, 132-138, doi:10.1016/j.applanim.2013.05.010.
344. Messam, L.L.M.; Hart, L.A. Persons Experiencing Prolonged Grief After the Loss of a Pet. In *Clinician's Guide to Treating Companion Animal Issues*; 2019; pp. 267-280.
345. Metraux, A. On Some Issues of Human-Animal Studies: An Introduction. *Sci Context* **2016**, *29*, 1-10, doi:10.1017/S0269889715000368.
346. Meyer, F.L.; McCrory, N.; Hewitt, L.; Peteet, J.R. Controversies Regarding Service Animals in the Ambulatory Oncology Setting. *J Oncol Pract* **2018**, *14*, 141-143, doi:10.1200/JOP.2017.026740.
347. Mickova, E.; Machova, K.; Dadova, K.; Svobodova, I. Does Dog Ownership Affect Physical Activity, Sleep, and Self-Reported Health in Older Adults? *Int J Environ Res Public Health* **2019**, *16*, doi:10.3390/ijerph16183355.
348. Mikkola, S.; Salonen, M.; Puurunen, J.; Hakanen, E.; Sulkama, S.; Araujo, C.; Lohi, H. Aggressive behaviour is affected by demographic, environmental and behavioural factors in purebred dogs. *Sci Rep* **2021**, *11*, 9433, doi:10.1038/s41598-021-88793-5.
349. Miklosi, A.; Topal, J. What does it take to become 'best friends'? Evolutionary changes in canine social competence. *Trends Cogn Sci* **2013**, *17*, 287-294, doi:10.1016/j.tics.2013.04.005.
350. Milani, M. The art of private veterinary practice. *Canadian Veterinary Journal* **2021**, *62*, 415-416.
351. Mills, G. Assessing the impact of Covid-19 on pets. *Veterinary Record* **2022**, *191*, 5, doi:10.1002/vetr.1983.
352. Min, K.D.; Kim, W.H.; Cho, S.; Cho, S.I. Owners' Attitudes toward Their Companion Dogs Are Associated with the Owners' Depression Symptoms-An Exploratory Study in South Korea. *Int J Environ Res Public Health* **2019**, *16*, doi:10.3390/ijerph16193567.
353. Minke, L.K. Normalization, Social Bonding, and Emotional Support— A Dog◎s Effect within a Prison Workshop for Women. *Anthrozoös* **2017**, *30*, 387-395, doi:10.1080/08927936.2017.1311065.
354. Miro, G.; Galvez, R.; Montoya, A.; Delgado, B.; Drake, J. Survey of Spanish pet owners about endoparasite infection risk and deworming frequencies. *Parasit Vectors* **2020**, *13*, 101, doi:10.1186/s13071-020-3976-8.
355. Miyake, K.; Kito, K.; Kotemori, A.; Sasaki, K.; Yamamoto, J.; Otagiri, Y.; Nagasawa, M.; Kuze-Arata, S.; Mogi, K.; Kikusui, T.; et al. Association between Pet Ownership and Obesity: A Systematic Review and Meta-Analysis. *Int J Environ Res Public Health* **2020**, *17*, doi:10.3390/ijerph17103498.
356. Mizukawa, H.; Nomiyama, K.; Nakatsu, S.; Yamamoto, M.; Ishizuka, M.; Ikenaka, Y.; Nakayama, S.M.M.; Tanabe, S. Anthropogenic and Naturally Produced Brominated Phenols in Pet Blood and Pet Food in Japan. *Environ Sci Technol* **2017**, *51*, 11354-11362, doi:10.1021/acs.est.7b01009.
357. Mongillo, P.; Pitteri, E.; Marinelli, L. Sustained attention to the owner is enhanced in dogs trained for animal assisted interventions. *Behav Processes* **2017**, *140*, 69-73, doi:10.1016/j.beproc.2017.03.024.
358. Montrose, V.T.; Squibb, K.; Hazel, S.; Kogan, L.R.; Oxley, J.A. Dog bites dog: The use of news media articles to investigate dog-on-dog aggression. *Journal of Veterinary Behavior* **2020**, *40*, 7-15, doi:10.1016/j.jveb.2020.08.002.
359. Moreira, R.L.; Gubert, F.D.; Sabino, L.M.; Benevides, J.L.; Tome, M.A.; Martins, M.C.; Brito, M.A. Assisted therapy with dogs in pediatric oncology: relatives' and nurses' perceptions. *Rev Bras Enferm* **2016**, *69*, 1188-1194, doi:10.1590/0034-7167-2016-0243.
360. Morelli, G.; Marchesini, G.; Contiero, B.; Fusi, E.; Diez, M.; Ricci, R. A Survey of Dog Owners' Attitudes toward Treats. *J Appl Anim Welf Sci* **2020**, *23*, 1-9, doi:10.1080/10888705.2019.1579095.
361. Mornement, K. Animals as Companions. In *Animals and Human Society*; 2018; pp. 281-304.
362. Mota-Rojas, D.; Calderon-Maldonado, N.; Lezama-Garcia, K.; Sepiurka, L.; Maria Garcia, R.C. Abandonment of dogs in Latin America: Strategies and ideas. *Vet World* **2021**, *14*, 2371-2379, doi:10.14202/vetworld.2021.2371-2379.

363. Mota-Rojas, D.; Marcet-Rius, M.; Ogi, A.; Hernandez-Avalos, I.; Mariti, C.; Martinez-Burnes, J.; Mora-Medina, P.; Casas, A.; Dominguez, A.; Reyes, B.; et al. Current Advances in Assessment of Dog's Emotions, Facial Expressions, and Their Use for Clinical Recognition of Pain. *Animals (Basel)* **2021**, *11*, doi:10.3390/ani11113334.
364. Mpolya, E.A. Encouraging responsible dog ownership in Africa. *Vet Rec* **2019**, *184*, 278-280, doi:10.1136/vr.l862.
365. Mubanga, M.; Byberg, L.; Egenvall, A.; Ingelsson, E.; Fall, T. Dog Ownership and Survival After a Major Cardiovascular Event: A Register-Based Prospective Study. *Circ Cardiovasc Qual Outcomes* **2019**, *12*, e005342, doi:10.1161/CIRCOUTCOMES.118.005342.
366. Mueller, M.K.; Gee, N.R.; Bures, R.M. Human-animal interaction as a social determinant of health: descriptive findings from the health and retirement study. *BMC Public Health* **2018**, *18*, 305, doi:10.1186/s12889-018-5188-0.
367. Mueller, M.K.; Richer, A.M.; Callina, K.S.; Charmaraman, L. Companion Animal Relationships and Adolescent Loneliness during COVID-19. *Animals (Basel)* **2021**, *11*, doi:10.3390/ani11030885.
368. Muller, C.A.; Riemer, S.; Rosam, C.M.; Schosswender, J.; Range, F.; Huber, L. Brief owner absence does not induce negative judgement bias in pet dogs. *Anim Cogn* **2012**, *15*, 1031-1035, doi:10.1007/s10071-012-0526-6.
369. Mulvaney-Roth, P.; Jackson, C.; Bert, L.; Eriksen, S.; Ryan, M. Using Pet Therapy to Decrease Patients' Anxiety on Two Diverse Inpatient Units. *J Am Psychiatr Nurses Assoc* **2023**, *29*, 112-121, doi:10.1177/1078390321999719.
370. Must, A.; Mule, C.M.; Linder, D.E.; Cash, S.B.; Folta, S.C. Animal-Assisted Intervention: A Promising Approach to Obesity Prevention for Youth With Autism Spectrum Disorder. *Front Vet Sci* **2021**, *8*, 646081, doi:10.3389/fvets.2021.646081.
371. Nagasawa, M.; Mitsui, S.; En, S.; Ohtani, N.; Ohta, M.; Sakuma, Y.; Onaka, T.; Mogi, K.; Kikusui, T. Oxytocin-gaze positive loop and the coevolution of human-dog bonds. *Science* **2015**, *348*, 333-336, doi:10.1126/science.1261022.
372. Nagasawa, M.; Mogi, K.; Ohtsuki, H.; Kikusui, T. Familiarity with humans affect dogs' tendencies to follow human majority groups. *Sci Rep* **2020**, *10*, 7119, doi:10.1038/s41598-020-64058-5.
373. Nagasawa, M.; Ogawa, M.; Mogi, K.; Kikusui, T. Intranasal Oxytocin Treatment Increases Eye-Gaze Behavior toward the Owner in Ancient Japanese Dog Breeds. *Front Psychol* **2017**, *8*, 1624, doi:10.3389/fpsyg.2017.01624.
374. Nagasawa, M.; Saito, M.; Hirasawa, H.; Mogi, K.; Kikusui, T. Dogs showed lower parasympathetic activity during mutual gazing while owners did not. *J Physiol Sci* **2023**, *73*, 9, doi:10.1186/s12576-023-00863-7.
375. Nakano, Y.; Matsushima, M.; Nakamori, A.; Hiroma, J.; Matsuo, E.; Wakabayashi, H.; Yoshida, S.; Ichikawa, H.; Kaneko, M.; Mutai, R.; et al. Depression and anxiety in pet owners after a diagnosis of cancer in their pets: a cross-sectional study in Japan. *BMJ Open* **2019**, *9*, e024512, doi:10.1136/bmjopen-2018-024512.
376. Narvekar, H.N. A Reflection on the Current Status of Animal-Assisted Therapy in India. *Human Arenas* **2021**, *6*, 760-775, doi:10.1007/s42087-021-00250-x.
377. Narvekar, H.N.; Narvekar, H.N. Canine-assisted Therapy in Neurodevelopmental Disorders: A Scoping Review. *European Journal of Integrative Medicine* **2022**, *50*, doi:10.1016/j.eujim.2022.102112.
378. Nelson, J.K.; Shih, P.C. CompanionViz: Mediated platform for gauging canine health and enhancing human–pet interactions. *International Journal of Human-Computer Studies* **2017**, *98*, 169-178, doi:10.1016/j.ijhcs.2016.04.002.
379. Ng, Z.; Albright, J.; Fine, A.H.; Peralta, J. Our Ethical and Moral Responsibility. In *Handbook on Animal-Assisted Therapy*; 2015; pp. 357-376.
380. Nguyen, T.; Clark, N.; Jones, M.K.; Herndon, A.; Mallyon, J.; Soares Magalhaes, R.J.; Abdullah, S. Perceptions of dog owners towards canine gastrointestinal parasitism and associated human health risk in Southeast Queensland. *One Health* **2021**, *12*, 100226, doi:10.1016/j.onehlt.2021.100226.
381. Nieforth, L.O.; Rodriguez, K.E.; O'Haire, M.E. Benefits and challenges of mobility and medical alert service dogs for caregivers of service dog recipients. *Disability and Rehabilitation: Assistive Technology* **2023**, *18*, 743-751, doi:10.1080/17483107.2021.1916630.
382. Niese, J.R.; Mepham, T.; Nielen, M.; Monninkhof, E.M.; Kroese, F.M.; de Ridder, D.T.D.; Corbee, R.J. Evaluating the Potential Benefit of a Combined Weight Loss Program in Dogs and Their Owners. *Front Vet Sci* **2021**, *8*, 653920, doi:10.3389/fvets.2021.653920.
383. Nitsch, R.; Kolle, P. The Impact of Optical Impressions on Dog Feeding Practice. *Top Companion Anim Med* **2022**, *50*, 100693, doi:10.1016/j.tcam.2022.100693.
384. Noli, C. Assessing Quality of Life for Pets with Dermatologic Disease and Their Owners. *Vet Clin North Am Small Anim Pract* **2019**, *49*, 83-93, doi:10.1016/j.cvsm.2018.08.008.
385. Ntampaka, P.; Nyaga, P.N.; Niragire, F.; Gathumbi, J.K.; Tukei, M. Knowledge, attitudes and practices regarding rabies and its control among dog owners in Kigali city, Rwanda. *PLoS One* **2019**, *14*, e0210044, doi:10.1371/journal.pone.0210044.
386. Nugent, W.R.; Daugherty, L. A Measurement Equivalence Study of the Family Bondedness Scale: Measurement Equivalence Between Cat and Dog Owners. *Front Vet Sci* **2021**, *8*, 812922, doi:10.3389/fvets.2021.812922.
387. O'Bryan, M. Family reflections: benefits for children with service dogs. *Pediatr Res* **2021**, *89*, 1032-1033, doi:10.1038/s41390-020-01289-2.
388. Offermans, J.E.; Duindam, H.M.; Asscher, J.J.; Stams, G.J.J.; Creemers, H.E. Brief report: The effectiveness of Dutch Cell Dogs: A multiple case experimental study. *Clin Child Psychol Psychiatry* **2020**, *25*, 1015-1021, doi:10.1177/1359104520940744.
389. Ogechi, I.; Snook, K.; Davis, B.M.; Hansen, A.R.; Liu, F.; Zhang, J. Pet Ownership and the Risk of Dying from Cardiovascular Disease Among Adults Without Major Chronic Medical Conditions. *High Blood Press Cardiovasc Prev* **2016**, *23*, 245-253, doi:10.1007/s40292-016-0156-1.
390. Ohkita, M.; Nagasawa, M.; Kazutaka, M.; Kikusui, T. Owners' direct gazes increase dogs' attention-getting behaviors. *Behav Processes* **2016**, *125*, 96-100, doi:10.1016/j.beproc.2016.02.013.

391. Okin, G.S. Environmental impacts of food consumption by dogs and cats. *PLoS One* **2017**, *12*, e0181301, doi:10.1371/journal.pone.0181301.
392. Oliva, J.L.; Johnston, K.L. Puppy love in the time of Corona: Dog ownership protects against loneliness for those living alone during the COVID-19 lockdown. *Int J Soc Psychiatry* **2021**, *67*, 232-242, doi:10.1177/0020764020944195.
393. Olmert, M. The Comfort Dog Project of Northern Uganda: An Innovative Canine-Assisted Psychosocial Trauma Recovery Programme. *Intervention* **2021**, *19*, doi:10.4103/intv.Intv_16_20.
394. Olsen, M.R. A case for methodological overhaul and increased study of executive function in the domestic dog (Canis lupus familiaris). *Anim Cogn* **2018**, *21*, 175-195, doi:10.1007/s10071-018-1162-6.
395. Orritt, R. Dog ownership has unknown risks but known health benefits: we need evidence based policy. *BMJ* **2014**, *349*, g4081, doi:10.1136/bmj.g4081.
396. Ortmeyer, H.K.; Robey, L.C. Companion Dog Foster Caregiver Program for Older Veterans at the VA Maryland Health Care System: A Feasibility Study. *Int J Environ Res Public Health* **2019**, *16*, doi:10.3390/ijerph16214285.
397. Overall, K.L. Standardization of assessments of behavior and welfare improves care. *Journal of Veterinary Behavior* **2019**, *32*, i-iii, doi:10.1016/j.jveb.2019.07.006.
398. Overgaauw, P.A.M.; Vinke, C.M.; Hagen, M.; Lipman, L.J.A. A One Health Perspective on the Human-Companion Animal Relationship with Emphasis on Zoonotic Aspects. *Int J Environ Res Public Health* **2020**, *17*, doi:10.3390/ijerph17113789.
399. Owczarczak-Garstecka, S.C.; Furtado, T.; Graham, T.M.; Lloyd, I.; Singleton, D.A.; Wallis, L.; Westgarth, C. Impacts of COVID-19 on Owner's Veterinary Healthcare Seeking Behavior for Dogs With Chronic Conditions: An Exploratory Mixed-Methods Study With a Convenience Sample. *Front Vet Sci* **2022**, *9*, 902219, doi:10.3389/fvets.2022.902219.
400. Oyama, M.A.; Citron, L.; Shults, J.; Cimino Brown, D.; Serpell, J.A.; Farrar, J.T. Measuring Quality of Life in Owners of Companion Dogs: Development and Validation of a Dog Owner-specific Quality of Life Questionnaire. *Anthrozoös* **2017**, *30*, 61-75, doi:10.1080/08927936.2016.1228774.
401. Packer, R.M.A.; O'Neill, D.G.; Fletcher, F.; Farnworth, M.J. Great expectations, inconvenient truths, and the paradoxes of the dog-owner relationship for owners of brachycephalic dogs. *PLoS One* **2019**, *14*, e0219918, doi:10.1371/journal.pone.0219918.
402. Packer, R.M.A.; O'Neill, D.G.; Fletcher, F.; Farnworth, M.J. Come for the looks, stay for the personality? A mixed methods investigation of reacquisition and owner recommendation of Bulldogs, French Bulldogs and Pugs. *PLoS One* **2020**, *15*, e0237276, doi:10.1371/journal.pone.0237276.
403. Packer, R.M.A.; Volk, H.A.; Fowkes, R.C. Physiological reactivity to spontaneously occurring seizure activity in dogs with epilepsy and their carers. *Physiol Behav* **2017**, *177*, 27-33, doi:10.1016/j.physbeh.2017.04.008.
404. Packman, W.; Bussolari, C.; Katz, R.; Carmack, B.J.; Field, N.P. Posttraumatic Growth Following the Loss of a Pet. *Omega (Westport)* **2017**, *75*, 337-359, doi:10.1177/0030222816663411.
405. Packman, W.; Carmack, B.J.; Katz, R.; Carlos, F.; Field, N.P.; Landers, C. Online survey as empathic bridging for the disenfranchised grief of pet loss. *Omega (Westport)* **2014**, *69*, 333-356, doi:10.2190/OM.69.4.a.
406. Panning, C.; Lem, M.; Bateman, S. Profiling a one-health model for priority populations. *Can J Public Health* **2016**, *107*, e222-e223, doi:10.17269/cjph.107.5463.
407. Patterson, D.; Simmonds, J.G.; Snell, T.L. "Savage Beasts," "Great Companions": The First Dogs to Winter on the Antarctic Continent. *Society & Animals* **2018**, *28*, 651-669, doi:10.1163/15685306-12341564.
408. Payne, E.; Boot, M.; Starling, M.; Henshall, C.; McLean, A.; Bennett, P.; McGreevy, P. Evidence of horsemanship and dogmanship and their application in veterinary contexts. *Vet J* **2015**, *204*, 247-254, doi:10.1016/j.tvjl.2015.04.004.
409. Payne, E.; DeAraugo, J.; Bennett, P.; McGreevy, P. Exploring the existence and potential underpinnings of dog-human and horse-human attachment bonds. *Behav Processes* **2016**, *125*, 114-121, doi:10.1016/j.beproc.2015.10.004.
410. Peacock, J.; Chur-Hansen, A.; Winefield, H. Mental health implications of human attachment to companion animals. *J Clin Psychol* **2012**, *68*, 292-303, doi:10.1002/jclp.20866.
411. Pearl, R.L.; Wadden, T.A.; Bach, C.; Leonard, S.M.; Michel, K.E. Who's a good boy? Effects of dog and owner body weight on veterinarian perceptions and treatment recommendations. *Int J Obes (Lond)* **2020**, *44*, 2455-2464, doi:10.1038/s41366-020-0622-7.
412. Pearson, C. "Four-Legged Poilus": French Army Dogs, Emotional Practices and the Creation of Militarized Human-Dog Bonds, 1871–1918. *Journal of Social History* **2019**, *52*, 731-760, doi:10.1093/jsh/shx090.
413. Pedretti, G.; Wirobski, G.; Range, F.; Marshall-Pescini, S. Artificially elevated oxytocin concentrations in pet dogs are associated with higher proximity-maintenance and gazing towards the owners. *Physiol Behav* **2021**, *237*, 113451, doi:10.1016/j.physbeh.2021.113451.
414. Pellegrino, L.D.; Cerimele, J.M.; Dubovsky, A.N. Service Dogs in the Hospital: Helpful or Harmful? A Case Report and Clinical Recommendations. *Psychosomatics* **2016**, *57*, 301-304, doi:10.1016/j.psym.2015.12.009.
415. Pendry, P.; Kuzara, S.; Gee, N.R. Evaluation of Undergraduate Students' Responsiveness to a 4-Week University-Based Animal-Assisted Stress Prevention Program. *Int J Environ Res Public Health* **2019**, *16*, doi:10.3390/ijerph16183331.
416. Perez Fraga, P.; Gerencser, L.; Andics, A. Human proximity seeking in family pigs and dogs. *Sci Rep* **2020**, *10*, 20883, doi:10.1038/s41598-020-77643-5.
417. Perez-Camargo, G.; Creagan, E.T. The design of visitation facilities to engage patients with their own cats and dogs. *Complement Ther Clin Pract* **2018**, *31*, 193-199, doi:10.1016/j.ctcp.2017.12.010.
418. Pergande, A.E.; Belshaw, Z.; Volk, H.A.; Packer, R.M.A. "We have a ticking time bomb": a qualitative exploration of the impact of canine epilepsy on dog owners living in England. *BMC Vet Res* **2020**, *16*, 443, doi:10.1186/s12917-020-02669-w.

419. Persson, M.E.; Trottier, A.J.; Belteky, J.; Roth, L.S.V.; Jensen, P. Intranasal oxytocin and a polymorphism in the oxytocin receptor gene are associated with human-directed social behavior in golden retriever dogs. *Horm Behav* **2017**, *95*, 85-93, doi:10.1016/j.yhbeh.2017.07.016.
420. Peterson, A.L. Canine Rescue as a Social Movement: The Politics of Love. *Society & Animals* **2018**, *28*, 670-687, doi:10.1163/15685306-12341555.
421. Peterson, H.; Engwall, K. “Why Would You Want a Baby When You Could Have a Dog?” Voluntarily Childless Women⊗s “Peternal” Feelings, Longing and Ambivalence. *Social Sciences* **2019**, *8*, doi:10.3390/socsci8040126.
422. Pfaller-Sadovsky, N.; Medina, L.; Dillenburger, K.; Hurtado-Parrado, C. We Don't Train in Vain: A Systematic Review and Meta-Analysis of Human and Canine Caregiver Training. *J Appl Anim Welf Sci* **2020**, *23*, 265-301, doi:10.1080/10888705.2019.1646134.
423.
424. Pinto-Garcia, L. Military Dogs and Their Soldier Companions: The More-than-human Biopolitics of Leishmaniasis in Conflict-torn Colombia. *Med Anthropol Q* **2022**, *36*, 237-255, doi:10.1111/maq.12694.
425. Pirrone, F.; Albertini, M.; Mazzola, S.M.; Pierantoni, L.; Bavagnoli, F.; Vigo, D. Correlation between the size of companion dogs and the profile of the owner: A cross-sectional study in Italy. *Dog Behavior* **2015**, *1*, 32-43, doi:10.4454/db.v1i2.14.
426. Pirrone, F.; Pierantoni, L.; Bossetti, A.; Uccheddu, S.; Albertini, M. Salivary Vasopressin as A Potential Non-Invasive Biomarker of Anxiety in Dogs Diagnosed with Separation-Related Problems. *Animals (Basel)* **2019**, *9*, doi:10.3390/ani9121033.
427. Pirrone, F.; Ripamonti, A.; Garoni, E.C.; Stradiotti, S.; Albertini, M. Measuring social synchrony and stress in the handler-dog dyad during animal-assisted activities: A pilot study. *Journal of Veterinary Behavior* **2017**, *21*, 45-52, doi:10.1016/j.jveb.2017.07.004.
428. Plata, E.; Montiel, S. Human-Dog Bond in the Contemporary Mayab: Social Perceptions and Benefits Associated with the Hunter-Milpa Dog in Maya Peasant-Hunter Life Strategies in Yucatan, Mexico. *Journal of Ethnobiology* **2023**, *40*, 451-464, doi:10.2993/0278-0771-40.4.451.
429. Porter, N. Training Dogs to Feel Good: Embodying Well-being in Multispecies Relations. *Med Anthropol Q* **2019**, *33*, 101-119, doi:10.1111/maq.12459.
430. Potter, K.; Chase, C.J. Leveraging the Human-Dog Bond to Support Physical Activity across the Life Span. *ACSM's Health and Fitness Journal* **2022**, *26*, 17-22, doi:10.1249/FIT.0000000000000780.
431. Potter, K.; Marcotte, R.T.; Petrucci, G.J.; Rajala, C.; Linder, D.E.; Balzer, L.B. Examining the Contribution of Dog Walking to Total Daily Physical Activity Among Dogs and Their Owners. *Journal for the Measurement of Physical Behaviour* **2021**, *4*, 97-101, doi:10.1123/jmpb.2020-0059.
432. Potter, K.; Masteller, B.; Balzer, L.B. Examining Obedience Training as a Physical Activity Intervention for Dog Owners: Findings from the Stealth Pet Obedience Training (SPOT) Pilot Study. *Int J Environ Res Public Health* **2021**, *18*, doi:10.3390/ijerph18030902.
433. Potter, K.; Rajala, C.; Chase, C.J.; LeBlanc, R. Testing Leash Walking Training as a Physical Activity Intervention for Older Adult Dog Owners: A Feasibility Study. *Geriatrics (Basel)* **2022**, *7*, doi:10.3390/geriatrics7060120.
434. Potter, K.; Sartore-Baldwin, M. Dogs as support and motivation for physical activity. *Current Sports Medicine Reports* **2019**, *18*, 275-280, doi:10.1249/JSR.0000000000000611.
435. Potter, K.; Teng, J.E.; Masteller, B.; Rajala, C.; Balzer, L.B. Examining How Dog 'Acquisition' Affects Physical Activity and Psychosocial Well-Being: Findings from the BuddyStudy Pilot Trial. *Animals (Basel)* **2019**, *9*, doi:10.3390/ani9090666.
436. Powell, L.; Chia, D.; McGreevy, P.; Podberscek, A.L.; Edwards, K.M.; Neilly, B.; Guastella, A.J.; Lee, V.; Stamatakis, E. Expectations for dog ownership: Perceived physical, mental and psychosocial health consequences among prospective adopters. *PLoS One* **2018**, *13*, e0200276, doi:10.1371/journal.pone.0200276.
437. Powell, L.; Edwards, K.M.; Bauman, A.; Guastella, A.J.; Drayton, B.; Stamatakis, E.; McGreevy, P. Canine Endogenous Oxytocin Responses to Dog-Walking and Affiliative Human(-)Dog Interactions. *Animals (Basel)* **2019**, *9*, doi:10.3390/ani9020051.
438. Powell, L.; Edwards, K.M.; Michael, S.; McGreevy, P.; Bauman, A.; Guastella, A.J.; Drayton, B.; Stamatakis, E. Effects of Human–Dog Interactions on Salivary Oxytocin Concentrations and Heart Rate Variability: A Four-Condition Cross-Over Trial. *Anthrozoös* **2020**, *33*, 37-52, doi:10.1080/08927936.2020.1694310.
439. Powell, L.; Guastella, A.J.; McGreevy, P.; Bauman, A.; Edwards, K.M.; Stamatakis, E. The physiological function of oxytocin in humans and its acute response to human-dog interactions: A review of the literature. *Journal of Veterinary Behavior* **2019**, *30*, 25-32, doi:10.1016/j.jveb.2018.10.008.
440. Powell, L.; Lee, B.; Reinhard, C.L.; Morris, M.; Satriale, D.; Serpell, J.; Watson, B. Returning a Shelter Dog: The Role of Owner Expectations and Dog Behavior. *Animals (Basel)* **2022**, *12*, doi:10.3390/ani12091053.
441. Prato Previde, E.; Valsecchi, P. The Immaterial Cord. In *The Social Dog*; 2014; pp. 165-189.
442. Prato-Previde, E.; Nicotra, V.; Fusar Poli, S.; Pelosi, A.; Valsecchi, P. Do dogs exhibit jealous behaviors when their owner attends to their companion dog? *Anim Cogn* **2018**, *21*, 703-713, doi:10.1007/s10071-018-1204-0.
443. Prato-Previde, E.; Nicotra, V.; Pelosi, A.; Valsecchi, P. Pet dogs' behavior when the owner and an unfamiliar person attend to a faux rival. *PLoS One* **2018**, *13*, e0194577, doi:10.1371/journal.pone.0194577.
444. Prato-Previde, E.; Pedretti, G.; Terruzzi, E.; Valsecchi, P. When the owner does not know: comparing puppies and adult dogs' showing behavior. *Anim Cogn* **2023**, *26*, 985-996, doi:10.1007/s10071-023-01744-7.
445. Pugliese, M.; Voslarova, E.; Biondi, V.; Passantino, A. Clinical Practice Guidelines: An Opinion of the Legal Implication to Veterinary Medicine. *Animals (Basel)* **2019**, *9*, doi:10.3390/ani9080577.

446. Rajagopaul, S.; Parr, J.M.; Woods, J.P.; Pearl, D.L.; Coe, J.B.; Verbrugghe, A. Owners' attitudes and practices regarding nutrition of dogs diagnosed with cancer presenting at a referral oncology service in Ontario, Canada. *J Small Anim Pract* **2016**, *57*, 484-490, doi:10.1111/jsap.12526.
447. Ramírez, M.T.G.; Berumen, L.C.Q.; Hernández, R.L. Psychometric properties of the Lexington attachment to pets scale: Mexican version (LAPS-M). *Anthrozoos* **2014**, *27*, 351-359, doi:10.2752/175303714X13903827487926.
448. Ramokapane, K.M.; van der Linden, D.; Zamansky, A. Does my dog really need a gadget? In Proceedings of the Proceedings of the Sixth International Conference on Animal-Computer Interaction, 2019; pp. 1-6.
449. Ramos, M.T.; Otto, C.M. Canine Mobility Maintenance and Promotion of a Healthy Lifestyle. *Vet Clin North Am Small Anim Pract* **2022**, *52*, 907-924, doi:10.1016/j.cvsm.2022.03.001.
450. Range, F.; Kassis, A.; Taborsky, M.; Boada, M.; Marshall-Pescini, S. Wolves and dogs recruit human partners in the cooperative string-pulling task. *Sci Rep* **2019**, *9*, 17591, doi:10.1038/s41598-019-53632-1.
451. Range, F.; Marshall-Pescini, S.; Kratz, C.; Viranyi, Z. Wolves lead and dogs follow, but they both cooperate with humans. *Sci Rep* **2019**, *9*, 3796, doi:10.1038/s41598-019-40468-y.
452. Rathish, D.; Rajapakse, R.P.V.J.; Weerakoon, K.G.A.D. The role of cortisol in the association of canine-companionship with blood pressure, glucose, and lipids: a systematic review. *High Blood Pressure and Cardiovascular Prevention* **2021**, *28*, 447-455, doi:10.1007/s40292-021-00469-3.
453. Rayment, D.J.; Peters, R.A.; Marston, L.C.; De Groef, B. Relationships between serum serotonin, plasma cortisol, and behavioral factors in a mixed-breed, -sex, and -age group of pet dogs. *Journal of Veterinary Behavior* **2020**, *38*, 96-102, doi:10.1016/j.jveb.2020.05.007.
454. Redding, L.E.; Kelly, B.J.; Stefanovski, D.; Lautenbach, J.K.; Tolomeo, P.; Cressman, L.; Gruber, E.; Meily, P.; Lautenbach, E. Pet Ownership Protects Against Recurrence of Clostridioides difficile Infection. *Open Forum Infect Dis* **2020**, *7*, ofz541, doi:10.1093/ofid/ofz541.
455. Reed, A.E.; DeYoung, S.E.; Farmer, A.K. Companion Animals and Online Discourse: Victim-Blaming and Animal Evacuation. *Anthrozoös* **2020**, *33*, 727-742, doi:10.1080/08927936.2020.1824654.
456. Reese, L.A.; Vertalka, J.J. Understanding Dog Bites: The Important Role of Human Behavior. *J Appl Anim Welf Sci* **2021**, *24*, 331-346, doi:10.1080/10888705.2020.1790371.
457. Reevy, G.M.; Delgado, M.M. Are emotionally attached companion animal caregivers conscientious and neurotic? Factors that affect the human-companion animal relationship. *J Appl Anim Welf Sci* **2015**, *18*, 239-258, doi:10.1080/10888705.2014.988333.
458. Rehn, T.; Keeling, L.J. Measuring dog-owner relationships: Crossing boundaries between animal behaviour and human psychology. *Applied Animal Behaviour Science* **2016**, *183*, 1-9, doi:10.1016/j.applanim.2016.07.003.
459. Rehn, T.; McGowan, R.T.; Keeling, L.J. Evaluating the Strange Situation Procedure (SSP) to assess the bond between dogs and humans. *PLoS One* **2013**, *8*, e56938, doi:10.1371/journal.pone.0056938.
460. Resnick, B. Can pets help make aging better? What do we know and believe? *Geriatr Nurs* **2019**, *40*, 121-122, doi:10.1016/j.gerinurse.2019.03.001.
461. Riddoch, K.A.; Hawkins, R.D.; Cross, E.S. Exploring behaviours perceived as important for human-Dog bonding and their translation to a robotic platform. *PLoS One* **2022**, *17*, e0274353, doi:10.1371/journal.pone.0274353.
462. Riggio, G.; Gazzano, A.; Zsilak, B.; Carlone, B.; Mariti, C. Quantitative Behavioral Analysis and Qualitative Classification of Attachment Styles in Domestic Dogs: Are Dogs with a Secure and an Insecure-Avoidant Attachment Different? *Animals (Basel)* **2020**, *11*, doi:10.3390/ani11010014.
463. Riggio, G.; Piotti, P.; Diverio, S.; Borrelli, C.; Di Iacovo, F.; Gazzano, A.; Howell, T.J.; Pirrone, F.; Mariti, C. The Dog-Owner Relationship: Refinement and Validation of the Italian C/DORS for Dog Owners and Correlation with the LAPS. *Animals (Basel)* **2021**, *11*, doi:10.3390/ani11082166.
464. Ritchie, L.; Quinn, S.; Tolson, D.; Jenkins, N.; Sharp, B. Exposing the mechanisms underlying successful animal-assisted interventions for people with dementia: A realistic evaluation of the Dementia Dog Project. *Dementia (London)* **2021**, *20*, 66-83, doi:10.1177/1471301219864505.
465. Robinson, J.L.; Baxi, M.; Katz, J.S.; Waggoner, P.; Beyers, R.; Morrison, E.; Salibi, N.; Denney, T.S.; Vodyanoy, V.; Deshpande, G. Characterization of Structural Connectivity of the Default Mode Network in Dogs using Diffusion Tensor Imaging. *Sci Rep* **2016**, *6*, 36851, doi:10.1038/srep36851.
466. Rodger, S.; Scott, E.M.; Nolan, A.; Wright, A.K.; Reid, J. Effect of Age, Breed, and Sex on the Health-Related Quality of Life of Owner Assessed Healthy Dogs. *Front Vet Sci* **2021**, *8*, 603139, doi:10.3389/fvets.2021.603139.
467. Rodrigo-Claverol, M.; Manuel-Canals, M.; Lobato-Rincon, L.L.; Rodriguez-Criado, N.; Roman-Casenave, M.; Musull-Dulcet, E.; Rodrigo-Claverol, E.; Pifarre, J.; Miro-Bernaus, Y. Human-Animal Bond Generated in a Brief Animal-Assisted Therapy Intervention in Adolescents with Mental Health Disorders. *Animals (Basel)* **2023**, *13*, doi:10.3390/ani13030358.
468. Rodriguez, K.E.; Bibbo, J.; O'Haire, M.E. The effects of service dogs on psychosocial health and wellbeing for individuals with physical disabilities or chronic conditions. *Disabil Rehabil* **2020**, *42*, 1350-1358, doi:10.1080/09638288.2018.1524520.
469. Rodriguez, K.E.; Bibbo, J.; Verdon, S.; O'Haire, M.E. Mobility and medical service dogs: a qualitative analysis of expectations and experiences. *Disabil Rehabil Assist Technol* **2020**, *15*, 499-509, doi:10.1080/17483107.2019.1587015.
470. Rodriguez, K.E.; LaFollette, M.R.; Hediger, K.; Ogata, N.; O'Haire, M.E. Defining the PTSD Service Dog Intervention: Perceived Importance, Usage, and Symptom Specificity of Psychiatric Service Dogs for Military Veterans. *Front Psychol* **2020**, *11*, 1638, doi:10.3389/fpsyg.2020.01638.

471. Romero, T.; Nagasawa, M.; Mogi, K.; Hasegawa, T.; Kikusui, T. Intranasal administration of oxytocin promotes social play in domestic dogs. *Commun Integr Biol* **2015**, *8*, e1017157, doi:10.1080/19420889.2015.1017157.
472. Roulaux, P.E.M.; van Herwijnen, I.R.; Beerda, B. Self-reports of Dutch dog owners on received professional advice, their opinions on castration and behavioural reasons for castrating male dogs. *PLoS One* **2020**, *15*, e0234917, doi:10.1371/journal.pone.0234917.
473. Roussel, C.; Drake, J.; Ariza, J.M. French national survey of dog and cat owners on the deworming behaviour and lifestyle of pets associated with the risk of endoparasites. *Parasit Vectors* **2019**, *12*, 480, doi:10.1186/s13071-019-3712-4.
474. Rubel, D.; Carbajo, A. Dogs in public spaces of Buenos Aires, Argentina: Exploring patterns of the abundance of dogs, the canine faecal contamination, the behaviour of people with dogs, and its relationships with demographic/economic variables. *Prev Vet Med* **2019**, *170*, 104713, doi:10.1016/j.prevetmed.2019.104713.
475. Rujoiu, O.; Rujoiu, V. Pet Loss and Human Emotion: Romanian Students' Reflections on Pet Loss. *Journal of Loss and Trauma* **2014**, *19*, 474-483, doi:10.1080/15325024.2013.806150.
476. Ruple, A.; Jones, M.; Simpson, M.; Page, R. The Golden Retriever Lifetime Study: Assessing factors associated with owner compliance after the first year of enrollment. *J Vet Intern Med* **2021**, *35*, 142-149, doi:10.1111/jvim.15921.
477. Russo, A.; Dowling-Guyer, S.; McCobb, E. Community Programming for Companion Dog Retention: A Survey of Animal Welfare Organizations. *J Appl Anim Welf Sci* **2023**, *26*, 117-131, doi:10.1080/10888705.2020.1869551.
478. Saberi, P.; Neilands, T.B.; Johnson, M.O. Association between Dog Guardianship and HIV Clinical Outcomes. *Journal of the International Association of Providers of AIDS Care (JIAPAC)* **2013**, *13*, 300-304, doi:10.1177/2325957413488832.
479. Sable, P. The Pet Connection: An Attachment Perspective. *Clinical Social Work Journal* **2012**, *41*, 93-99, doi:10.1007/s10615-012-0405-2.
480. Salamon, A.; Szaraz, J.; Miklosi, A.; Gacsi, M. Movement and vocal intonation together evoke social referencing in companion dogs when confronted with a suspicious stranger. *Anim Cogn* **2020**, *23*, 913-924, doi:10.1007/s10071-020-01401-3.
481. Salgado-Caxito, M.; Benavides, J.A.; Atero, N.; Cordova-Burhle, F.; Ramos, R.; Fernandez, M.; Sapiente-Aguirre, C.; Mardones, F.O. Preventive healthcare among dogs and cats in Chile is positively associated with emotional owner-companion animal bond and socioeconomic factors. *Prev Vet Med* **2023**, *213*, 105882, doi:10.1016/j.prevetmed.2023.105882.
482. Salonen, M.; Sulkama, S.; Mikkola, S.; Puurunen, J.; Hakanen, E.; Tiira, K.; Araujo, C.; Lohi, H. Prevalence, comorbidity, and breed differences in canine anxiety in 13,700 Finnish pet dogs. *Sci Rep* **2020**, *10*, 2962, doi:10.1038/s41598-020-59837-z.
483. San Jose, R.D.; Magsino, P.J.P.; Bundalian, R.D.L. Factors affecting the knowledge, attitude, and practices of pet owners on responsible pet ownership in magalang, Pampanga, Philippines: A cross-sectional study. *Philippine Journal of Veterinary Medicine* **2020**, *57*, 182-195.
484. Sanchez-Soriano, C.; Gibson, A.D.; Gamble, L.; Bailey, J.L.B.; Mayer, D.; Lohr, F.; Chikungwa, P.; Chulu, J.; Handel, I.G.; Bronsvoort, B.M.D.; et al. Implementation of a mass canine rabies vaccination campaign in both rural and urban regions in southern Malawi. *PLoS Negl Trop Dis* **2020**, *14*, e0008004, doi:10.1371/journal.pntd.0008004.
485. Sandoe, P.; Palmer, C.; Corr, S.; Astrup, A.; Bjornvad, C.R. Canine and feline obesity: a One Health perspective. *Vet Rec* **2014**, *175*, 610-616, doi:10.1136/vr.g7521.
486. Sanford, E.M.; Burt, E.R.; Meyers-Manor, J.E. Timmy's in the well: Empathy and prosocial helping in dogs. *Learn Behav* **2018**, *46*, 374-386, doi:10.3758/s13420-018-0332-3.
487. Sarkar, R.; Sau, S.; Bhadra, A. Scavengers can be choosers: A study on food preference in free-ranging dogs. *Applied Animal Behaviour Science* **2019**, *216*, 38-44, doi:10.1016/j.applanim.2019.04.012.
488. Sarlon, J.; Staniloiu, A.; Schontges, A.; Kordon, A. Vegetative symptoms and behaviour of the therapy-accompanying dog of a chronically suicidal patient. *BMJ Case Rep* **2018**, *2018*, doi:10.1136/bcr-2018-225483.
489. Saunders, J.; Parast, L.; Babey, S.H.; Miles, J.V. Exploring the differences between pet and non-pet owners: Implications for human-animal interaction research and policy. *PLoS One* **2017**, *12*, e0179494, doi:10.1371/journal.pone.0179494.
490. Savadogo, M.; Tialla, D.; Ouattara, B.; Dahourou, L.D.; Ossebi, W.; Ilboudo, S.G.; Combari, A.H.B.; Tarnagda, Z.; Alambedji, R.B. Factors associated with owned-dogs' vaccination against rabies: A household survey in Bobo Dioulasso, Burkina Faso. *Vet Med Sci* **2021**, *7*, 1096-1106, doi:10.1002/vms3.468.
491. Savalli, C.; Resende, B.; Gaunet, F. Eye Contact Is Crucial for Referential Communication in Pet Dogs. *PLoS One* **2016**, *11*, e0162161, doi:10.1371/journal.pone.0162161.
492. Savel, S.; Sombe, P. Are dogs with congenital hearing and/or vision impairments so different from sensory normal dogs? A survey of demographics, morphology, health, behaviour, communication, and activities. *PLoS One* **2020**, *15*, e0230651, doi:10.1371/journal.pone.0230651.
493. Scagnetto, F.; Simionato, I.; Benedetti, D.; Notari, V. A new perspective on the bond between human beings and animals: A study on the human-dog and human-horse relationship. *Dog Behavior* **2021**, *7*, 19-34, doi:10.4454/db.v7i1.133.
494. Scandurra, A.; Alterisio, A.; D@Aniello, B. Behavioural effects of training on water rescue dogs in the Strange Situation Test. *Applied Animal Behaviour Science* **2016**, *174*, 121-127, doi:10.1016/j.applanim.2015.10.007.
495. Scanlon, L.; Hobson-West, P.; Cobb, K.; McBride, A.; Stavisky, J. Homeless People and Their Dogs: Exploring the Nature and Impact of the Human–Companion Animal Bond. *Anthrozoös* **2021**, *34*, 77-92, doi:10.1080/08927936.2021.1878683.
496. Scarborough, R.; Hardefeldt, L.; Browning, G.; Bailey, K. Pet Owners and Antibiotics: Knowledge, Opinions, Expectations, and Communication Preferences. *Antibiotics (Basel)* **2021**, *10*, doi:10.3390/antibiotics10111326.

497. Schenk, G.; Duindam, H.M.; Creemers, H.E.; Hoeve, M.; Stams, G.; Asscher, J.J. The effectiveness of Dutch Cell Dogs in correctional facilities in the Netherlands: a study protocol of a quasi-experimental trial. *BMC Psychiatry* **2018**, *18*, 218, doi:10.1186/s12888-018-1797-5.
498. Schoberl, I.; Wedl, M.; Beetz, A.; Kotrschal, K. Psychobiological Factors Affecting Cortisol Variability in Human-Dog Dyads. *PLoS One* **2017**, *12*, e0170707, doi:10.1371/journal.pone.0170707.
499. Schoenfeld-Tacher, R.M.; Kogan, L.R. The Human-Animal Bond and Hispanic Clients in the United States. In *Clinician's Guide to Treating Companion Animal Issues*; 2019; pp. 457-475.
500. Schor, M.; Protopopova, A. Effect of COVID-19 on Pet Food Bank Servicing: Quantifying Numbers of Clients Serviced in the Vancouver Downtown Eastside, British Columbia, Canada. *Front Vet Sci* **2021**, *8*, 730390, doi:10.3389/fvets.2021.730390.
501. Schunemann, B.; Keller, J.; Rakoczy, H.; Behne, T.; Brauer, J. Dogs distinguish human intentional and unintentional action. *Sci Rep* **2021**, *11*, 14967, doi:10.1038/s41598-021-94374-3.
502.
503. Schuurmans, L.; Enders-Slegers, M.J.; Verheggen, T.; Schols, J. Animal-Assisted Interventions in Dutch Nursing Homes: A Survey. *J Am Med Dir Assoc* **2016**, *17*, 647-653, doi:10.1016/j.jamda.2016.03.015.
504. Schwarzmueller-Erber, G.; Maier, M.; Kundi, M. Pet Attachment and Wellbeing of Older-Aged Recreational Horseback Riders. *Int J Environ Res Public Health* **2020**, *17*, doi:10.3390/ijerph17061865.
505. Serpell, J.A. How happy is your pet? The problem of subjectivity in the assessment of companion animal welfare. *Animal Welfare* **2023**, *28*, 57-66, doi:10.7120/09627286.28.1.057.
506. Severson, R.L. The Value of (Research on) Animals in Children's Lives. *Human Development* **2014**, *57*, 26-29, doi:10.1159/000357792.
507. Shah, S.S.A.; Khan, M.I.; Khan, M.A.; Khan, H.; Ali, A.; Ali, M.I.; Jan, R. Tick-borne diseases-possible threat to humans-dog interspecie bond. *Advances in Animal and Veterinary Sciences* **2017**, *5*, 115-120, doi:10.14737/journal.aavs/2017/5.3.115.120.
508. Shen, R.Z.Z.; Xiong, P.; Chou, U.I.; Hall, B.J. "We need them as much as they need us": A systematic review of the qualitative evidence for possible mechanisms of effectiveness of animal-assisted intervention (AAI). *Complement Ther Med* **2018**, *41*, 203-207, doi:10.1016/j.ctim.2018.10.001.
509. Sherlock, C.; Holland, C.V.; Keegan, J.D. Caring for Canines: A Survey of Dog Ownership and Parasite Control Practices in Ireland. *Vet Sci* **2023**, *10*, doi:10.3390/vetsci10020090.
510. Sherman, G.D.; Rice, L.K.; Jin, E.S.; Jones, A.C.; Josephs, R.A. Sex differences in cortisol's regulation of affiliative behavior. *Horm Behav* **2017**, *92*, 20-28, doi:10.1016/j.yhbeh.2016.12.005.
511. Shoesmith, E.; Santos de Assis, L.; Shahab, L.; Ratschen, E.; Toner, P.; Kale, D.; Reeve, C.; Mills, D.S. The Perceived Impact of The First UK COVID-19 Lockdown on Companion Animal Welfare and Behaviour: A Mixed-Method Study of Associations with Owner Mental Health. *Int J Environ Res Public Health* **2021**, *18*, doi:10.3390/ijerph18116171.
512. Siebenbruner, J. Companion Animals in Childhood and Emerging Adulthood: The Relation to Emerging Adult Development. *Society & Animals* **2019**, *27*, 235-253, doi:10.1163/15685306-12341522.
513. Siniscalchi, M.; Stipo, C.; Quaranta, A. "Like owner, like dog": correlation between the owner's attachment profile and the owner-dog bond. *PLoS One* **2013**, *8*, e78455, doi:10.1371/journal.pone.0078455.
514. Smith, B.P.; Hazelton, P.C.; Thompson, K.R.; Trigg, J.L.; Etherton, H.C.; Blunden, S.L. A Multispecies Approach to Co-Sleeping : Integrating Human-Animal Co-Sleeping Practices into Our Understanding of Human Sleep. *Hum Nat* **2017**, *28*, 255-273, doi:10.1007/s12110-017-9290-2.
515. Sohal, J.S.; Khan, A.; Vats, D.; Jain, M.; Polavarapu, R.; Aseri, G.K.; Sharma, D. Applications of genome editing in pet world. In *Genomics and Biotechnological Advances in Veterinary, Poultry, and Fisheries*; 2020; pp. 151-162.
516. Solhjoo, N. Using the pet health information behaviour intervention model should make information prescriptions for pet guardians more effective. *Health Info Libr J* **2024**, *41*, 26-42, doi:10.1111/hir.12456.
517. /14616734.2018.1517812.
518. Sorensen, I.K.; Bidstrup, P.E.; Rod, N.H.; Ruhling, T.; Johansen, C. Is dog ownership associated with mortality? A nationwide registry study. *Eur J Public Health* **2018**, *28*, 1169-1171, doi:10.1093/eurpub/cky164.
519. Spitznagel, M.B.; Hillier, A.; Gober, M.; Carlson, M.D. Treatment complexity and caregiver burden are linked in owners of dogs with allergic/atopic dermatitis. *Vet Dermatol* **2021**, *32*, 192-e150, doi:10.1111/vde.12938.
520. Spitznagel, M.B.; Patrick, K.; Gober, M.W.; Carlson, M.D.; Gardner, M.; Shaw, K.K.; Coe, J.B. Relationships among owner consideration of euthanasia, caregiver burden, and treatment satisfaction in canine osteoarthritis. *Vet J* **2022**, *286*, 105868, doi:10.1016/j.tvjl.2022.105868.
521. Srimoragot, P.; Sudsakorn, P.; Suwannaphirom, P.; Ruchisereekul, K. Survey of knowledge and opinion among thai veterinarians on nicotine toxicity and second-hand smoke effects on pets' health. *Thai Journal of Veterinary Medicine* **2021**, *51*, 69-74.
522. Stahl, P.W. Old dogs and new tricks: Recent developments in our understanding of the human–dog relationship. *Reviews in Anthropology* **2016**, *45*, 51-68, doi:10.1080/00938157.2016.1142298.
523. Steagall, P.V.; Monteiro, B.P.; Ruel, H.L.M.; Beauchamp, G.; Luca, G.; Berry, J.; Little, S.; Stiles, E.; Hamilton, S.; Pang, D. Perceptions and opinions of Canadian pet owners about anaesthesia, pain and surgery in small animals. *J Small Anim Pract* **2017**, *58*, 380-388, doi:10.1111/jsap.12674.
524. Sterneberg-van der Maaten, T.; Turner, D.; Van Tilburg, J.; Vaarten, J. Benefits and Risks for People and Livestock of Keeping Companion Animals: Searching for a Healthy Balance. *J Comp Pathol* **2016**, *155*, S8-S17, doi:10.1016/j.jcpa.2015.06.007.

525. Stevens, P.; Kepros, J.P.; Mosher, B.D. Use of a Dog Visitation Program to Improve Patient Satisfaction in Trauma Patients. *J Trauma Nurs* **2017**, *24*, 97-101, doi:10.1097/JTN.0000000000000272.
526. Stoeckel, L.E.; Palley, L.S.; Gollub, R.L.; Niemi, S.M.; Evins, A.E. Patterns of brain activation when mothers view their own child and dog: an fMRI study. *PLoS One* **2014**, *9*, e107205, doi:10.1371/journal.pone.0107205.
527. Su, B.; Koda, N.; Martens, P. How Japanese companion dog and cat owners' degree of attachment relates to the attribution of emotions to their animals. *PLoS One* **2018**, *13*, e0190781, doi:10.1371/journal.pone.0190781.
528. Suarez, L.; Bautista-Castano, I.; Pena Romera, C.; Montoya-Alonso, J.A.; Corbera, J.A. Is Dog Owner Obesity a Risk Factor for Canine Obesity? A "One-Health" Study on Human-Animal Interaction in a Region with a High Prevalence of Obesity. *Vet Sci* **2022**, *9*, doi:10.3390/vetsci9050243.
529. Sugawara, A.; Masud, M.M.; Yokoyama, A.; Mizutani, W.; Watanuki, S.; Yanai, K.; Itoh, M.; Tashiro, M. Effects of Presence of a Familiar Pet Dog on Regional Cerebral Activity in Healthy Volunteers: A Positron Emission Tomography Study. *Anthrozoös* **2015**, *25*, 25-34, doi:10.2752/175303712x13240472427311.
530. Suici, T.; DărăbuȘ, G.; Mederle, N.; Sîrbu, C.; Imre, M.; Morariu, S. The effect of atopic dermatitis on quality of life of affected dogs and their owners in Romania. *Journal of Veterinary Behavior* **2021**, *46*, 24-30, doi:10.1016/j.jveb.2021.07.006.
531. Sundell, I.B. What can i do when ruger is limping? *Journal of Stem Cells* **2018**, *13*, 107-112.
532. Sundman, A.S.; Van Poucke, E.; Svensson Holm, A.C.; Faresjo, A.; Theodorsson, E.; Jensen, P.; Roth, L.S.V. Long-term stress levels are synchronized in dogs and their owners. *Sci Rep* **2019**, *9*, 7391, doi:10.1038/s41598-019-43851-x.
533. Surma, S.; Oparil, S.; Narkiewicz, K. Pet Ownership and the Risk of Arterial Hypertension and Cardiovascular Disease. *Curr Hypertens Rep* **2022**, *24*, 295-302, doi:10.1007/s11906-022-01191-8.
534. Szantho, F.; Miklosi, A.; Kubinyi, E. Is your dog empathic? Developing a Dog Emotional Reactivity Survey. *PLoS One* **2017**, *12*, e0170397, doi:10.1371/journal.pone.0170397.
535. Tague, I.H. The history of emotional attachment to animals. In *The Routledge Companion to Animal-Human History*; Taylor and Francis: 2018; pp. 345-366.
536. Takashima, G.K.; Day, M.J. Setting the One Health agenda and the human-companion animal bond. *Int J Environ Res Public Health* **2014**, *11*, 11110-11120, doi:10.3390/ijerph111111110.
537. Tan, J.; Walker, K.K.; Hoff, K.; Hare, B. What influences a pet dog's first impression of a stranger? *Learn Behav* **2018**, *46*, 414-429, doi:10.3758/s13420-018-0353-y.
538. Taniguchi, Y.; Seino, S.; Nishi, M.; Tomine, Y.; Tanaka, I.; Yokoyama, Y.; Ikeuchi, T.; Kitamura, A.; Shinkai, S. Association of Dog and Cat Ownership with Incident Frailty among Community-Dwelling Elderly Japanese. *Sci Rep* **2019**, *9*, 18604, doi:10.1038/s41598-019-54955-9.
539. Taylor, N. ©We need a new approach to dangerous dogs© *Veterinary Record* **2022**, *190*, 331, doi:10.1002/vetr.1716.
540. Teo, J.T.; Johnstone, S.J.; Romer, S.S.; Thomas, S.J. Psychophysiological mechanisms underlying the potential health benefits of human-dog interactions: A systematic literature review. *Int J Psychophysiol* **2022**, *180*, 27-48, doi:10.1016/j.ijpsycho.2022.07.007.
541. Tepfer, A.; Ross, S.; MacDonald, M.; Udell, M.A.R.; Ruaux, C.; Baltzer, W. Family Dog-Assisted Adapted Physical Activity: A Case Study. *Animals (Basel)* **2017**, *7*, doi:10.3390/ani7050035.
542. Testoni, I.; De Vincenzo, C.; Campigli, M.; Caregnato Manzatti, A.; Ronconi, L.; Uccheddu, S. Validation of the HHHHHMM Scale in the Italian Context: Assessing Pets' Quality of Life and Qualitatively Exploring Owners' Grief. *Animals (Basel)* **2023**, *13*, doi:10.3390/ani13061049.
543. Thielke, L.E.; Udell, M.A.R. Characterizing Human-Dog Attachment Relationships in Foster and Shelter Environments as a Potential Mechanism for Achieving Mutual Wellbeing and Success. *Animals (Basel)* **2019**, *10*, doi:10.3390/ani10010067.
544. Thompkins, A.M.; Lazarowski, L.; Ramaiahgari, B.; Gotoor, S.S.R.; Waggoner, P.; Denney, T.S.; Deshpande, G.; Katz, J.S. Dog-human social relationship: representation of human face familiarity and emotions in the dog brain. *Anim Cogn* **2021**, *24*, 251-266, doi:10.1007/s10071-021-01475-7.
545. Thys, S.; Knobel, D.L.; Simpson, G.; Rooyen, J.V.; Marcotty, T.; Gabriël, S.; Dorny, P.; Boelaert, M. Perceptions and Practices of Dog Ownership and Rabies Control at a Human–Wildlife–Domestic Animal Interface in South Africa. *Anthrozoös* **2021**, *34*, 281-302, doi:10.1080/08927936.2021.1885146.
546. Tomlinson, C.A.; Matijczak, A.; McDonald, S.E.; Gee, N.R. The role of human-animal interaction in child and adolescent health and development. In *Encyclopedia of Child and Adolescent Health*; 2023; pp. 564-577.
547. Tops, M.; Huijbregts, S.C.J.; Buisman-Pijlman, F.T.A. Commentary: Intranasal Oxytocin Treatment Increases Eye-Gaze Behavior toward the Owner in Ancient Japanese Dog Breeds. *Front Psychol* **2018**, *9*, 1473, doi:10.3389/fpsyg.2018.01473.
548. Treves-Brown, K. Recognising fish as animals. *Vet Rec* **2020**, *187*, 195, doi:10.1136/vr.m3235.
549. Tseng, A. Brief Report: Above and Beyond Safety: Psychosocial and Biobehavioral Impact of Autism-Assistance Dogs on Autistic Children and their Families. *J Autism Dev Disord* **2023**, *53*, 468-483, doi:10.1007/s10803-021-05410-0.
550. Turcsan, B.; Miklosi, A.; Kubinyi, E. Owner perceived differences between mixed-breed and purebred dogs. *PLoS One* **2017**, *12*, e0172720, doi:10.1371/journal.pone.0172720.
551. Turcsan, B.; Szantho, F.; Miklosi, A.; Kubinyi, E. Fetching what the owner prefers? Dogs recognize disgust and happiness in human behaviour. *Anim Cogn* **2015**, *18*, 83-94, doi:10.1007/s10071-014-0779-3.
552. Tzivian, L.; Friger, M.; Kushnir, T. Associations between stress and quality of life: differences between owners keeping a living dog or losing a dog by euthanasia. *PLoS One* **2015**, *10*, e0121081, doi:10.1371/journal.pone.0121081.

553. Uccheddu, S.; De Cataldo, L.; Albertini, M.; Coren, S.; Da Graca Pereira, G.; Haverbeke, A.; Mills, D.S.; Pierantoni, L.; Riemer, S.; Ronconi, L.; et al. Pet Humanisation and Related Grief: Development and Validation of a Structured Questionnaire Instrument to Evaluate Grief in People Who Have Lost a Companion Dog. *Animals (Basel)* **2019**, *9*, doi:10.3390/ani9110933.
554. Udell, M.A.R.; Brubaker, L. Are Dogs Social Generalists? Canine Social Cognition, Attachment, and the Dog-Human Bond. *Current Directions in Psychological Science* **2016**, *25*, 327-333, doi:10.1177/0963721416662647.
555. Ujfalussy, D.J.; Viranyi, Z.; Gacsi, M.; Farago, T.; Pogany, A.; Bereczky, B.M.; Miklosi, A.; Kubinyi, E. Comparing the tractability of young hand-raised wolves (Canis lupus) and dogs (Canis familiaris). *Sci Rep* **2020**, *10*, 14678, doi:10.1038/s41598-020-71687-3.
556. Van Bourg, J.; Patterson, J.E.; Wynne, C.D.L. Pet dogs (Canis lupus familiaris) release their trapped and distressed owners: Individual variation and evidence of emotional contagion. *PLoS One* **2020**, *15*, e0231742, doi:10.1371/journal.pone.0231742.
557. van der Linden, D.; Davidson, B.I.; Zamansky, A. The not so secret life of pets. In Proceedings of the Proceedings of the Sixth International Conference on Animal-Computer Interaction, 2019; pp. 1-6.
558. van der Linden, D.; Williams, E.; Hadar, I.; Zamansky, A. Some might freak out. In Proceedings of the Proceedings of the Sixth International Conference on Animal-Computer Interaction, 2019; pp. 1-12.
559. van Houtert, E.A.E.; Endenburg, N.; Wijnker, J.J.; Rodenburg, T.B.; van Lith, H.A.; Vermetten, E. The Translation and Validation of the Dutch Monash Dog(-)Owner Relationship Scale (MDORS). *Animals (Basel)* **2019**, *9*, doi:10.3390/ani9050249.
560. Vanutelli, M.E.; Balconi, M. Perceiving emotions in human-human and human-animal interactions: Hemodynamic prefrontal activity (fNIRS) and empathic concern. *Neurosci Lett* **2015**, *605*, 1-6, doi:10.1016/j.neulet.2015.07.020.
561. Vegas Comitre, M.D.; Palmer, L.; Bacek, L.M.; Kuo, K.W.; Keys, D. Assessment of prehospital care in canine trauma patients presented to Veterinary Trauma Centers: A VetCOT registry study. *J Vet Emerg Crit Care (San Antonio)* **2021**, *31*, 788-794, doi:10.1111/vec.13105.
562. Veloso, E.C.M.; Negreiros, A.D.S.; da Silva, J.P.; Moura, L.D.; Nascimento, L.F.M.; Silva, T.S.; Werneck, G.L.; Cruz, M. Socioeconomic and environmental factors associated with the occurrence of canine infection by Leishmania infantum in Teresina, Brazil. *Vet Parasitol Reg Stud Reports* **2021**, *24*, 100561, doi:10.1016/j.vprsr.2021.100561.
563. Vieira, L.C.; Contesini, E.A.; Goldim, J.R. Cancer diagnosis and treatment of children and dogs: coping strategies used by parents and owners in teaching hospitals. *Brazilian Journal of Veterinary Medicine* **2021**, *43*, doi:10.29374/2527-2179.bjvm002220.
564. Vincent, A. Dog Parks as an Institutional Resource for Social Capital in the Urban Neighborhood. *Society & Animals* **2019**, *29*, 517-538, doi:10.1163/15685306-00001598.
565. Viviers, H. The psychology of animal companionship: Some ancient and modern views. *HTS Teologiese Studies / Theological Studies* **2014**, *70*, doi:10.4102/hts.v70i1.2705.
566. von Rentzell, K.A.; van Haaften, K.; Morris, A.; Protopopova, A. Investigation into owner-reported differences between dogs born in versus imported into Canada. *PLoS One* **2022**, *17*, e0268885, doi:10.1371/journal.pone.0268885.
567. Vucinic, M.; Vucicevic, M.; Nenadovic, K. The COVID-19 pandemic affects owners walking with their dogs. *J Vet Behav* **2022**, *48*, 1-10, doi:10.1016/j.jveb.2021.10.009.
568. Waller, B.M.; Peirce, K.; Caeiro, C.C.; Scheider, L.; Burrows, A.M.; McCune, S.; Kaminski, J. Paedomorphic facial expressions give dogs a selective advantage. *PLoS One* **2013**, *8*, e82686, doi:10.1371/journal.pone.0082686.
569. Wallis, L.J.; Szabo, D.; Erdelyi-Belle, B.; Kubinyi, E. Demographic Change Across the Lifespan of Pet Dogs and Their Impact on Health Status. *Front Vet Sci* **2018**, *5*, 200, doi:10.3389/fvets.2018.00200.
570. Wallis, L.J.; Szabo, D.; Kubinyi, E. Cross-Sectional Age Differences in Canine Personality Traits; Influence of Breed, Sex, Previous Trauma, and Dog Obedience Tasks. *Front Vet Sci* **2019**, *6*, 493, doi:10.3389/fvets.2019.00493.
571. Walther, B.; Tedin, K.; Lubke-Becker, A. Multidrug-resistant opportunistic pathogens challenging veterinary infection control. *Vet Microbiol* **2017**, *200*, 71-78, doi:10.1016/j.vetmic.2016.05.017.
572. Wanser, S.H.; MacDonald, M.; Udell, M.A.R. Dog-human behavioral synchronization: family dogs synchronize their behavior with child family members. *Anim Cogn* **2021**, *24*, 747-752, doi:10.1007/s10071-020-01454-4.
573. Wanser, S.H.; Simpson, A.C.; MacDonald, M.; Udell, M.A.R. Considering Family Dog Attachment Bonds: Do Dog-Parent Attachments Predict Dog-Child Attachment Outcomes in Animal-Assisted Interventions? *Front Psychol* **2020**, *11*, 566910, doi:10.3389/fpsyg.2020.566910.
574. Webb, T.L.; Krasuska, M.; Toth, Z.; du Plessis, H.R.; Colliard, L. Using research on self-regulation to understand and tackle the challenges that owners face helping their (overweight) dogs lose weight. *Prev Vet Med* **2018**, *159*, 227-231, doi:10.1016/j.prevetmed.2018.08.017.
575. Westgarth, C.; Boddy, L.M.; Stratton, G.; German, A.J.; Gaskell, R.M.; Coyne, K.P.; Bundred, P.; McCune, S.; Dawson, S. The association between dog ownership or dog walking and fitness or weight status in childhood. *Pediatr Obes* **2017**, *12*, e51-e56, doi:10.1111/ijpo.12176.
576. Westgarth, C.; Christian, H.E.; Christley, R.M. Factors associated with daily walking of dogs. *BMC Vet Res* **2015**, *11*, 116, doi:10.1186/s12917-015-0434-5.
577. While, A. Pet dogs as promoters of wellbeing. *British Journal of Community Nursing* **2017**, *22*, 332-336, doi:10.12968/bjcn.2017.22.7.332.
578. White, G.A.; Ward, L.; Pink, C.; Craigon, J.; Millar, K.M. "Who's been a good dog?" - Owner perceptions and motivations for treat giving. *Prev Vet Med* **2016**, *132*, 14-19, doi:10.1016/j.prevetmed.2016.08.002.
579. White, N.; Mills, D.; Hall, S. Attachment Style Is Related to Quality of Life for Assistance Dog Owners. *Int J Environ Res Public Health* **2017**, *14*, doi:10.3390/ijerph14060658.

580. Whitebird, R.R.; Solberg, L.I. What's Important: Postoperative Care Planning. Recognizing the Central Role of Pets in Many Patients' Lives. *J Bone Joint Surg Am* **2021**, *103*, 1663-1664, doi:10.2106/JBJS.21.00099.
581. Wiewiorowski, J.; Dostalík, P. The specific position of the animal, especially a dog in the Roman and modern Czech law. *Lawyer Quarterly* **2017**, *7*, 12-28.
582. Wilkin, C.L.; Fairlie, P.; Ezzedeen, S.R. Who let the dogs in? A look at pet-friendly workplaces. *International Journal of Workplace Health Management* **2016**, *9*, 96-109, doi:10.1108/ijwhm-04-2015-0021.
583. Williams, M.; Varelas, E.N.; Olmsted, Z.T.; Sheldon, B.L.; Khazen, O.; DiMarzio, M.; Pilitsis, J.G. Can dogs and cats really help our spinal cord stimulation patients? *Clin Neurol Neurosurg* **2021**, *208*, 106831, doi:10.1016/j.clineuro.2021.106831.
584. Willis, E.M.; Ross, K.E. Review of principles governing dog health education in remote Aboriginal communities. *Aust Vet J* **2019**, *97*, 4-9, doi:10.1111/avj.12776.
585. Wlodarczyk, J. Post-Communist Canine: A Feminist Approach to Women and Dogs in Canine Performance Sports in Poland. *Society & Animals* **2016**, *24*, 129-152, doi:10.1163/15685306-12341398.
586. Woo, B.M.; Schaller, M. "Parental" responses to human infants (and puppy dogs): Evidence that the perception of eyes is especially influential, but eye contact is not. *PLoS One* **2020**, *15*, e0232059, doi:10.1371/journal.pone.0232059.
587. Wood, L.; Martin, K.; Christian, H.; Nathan, A.; Lauritsen, C.; Houghton, S.; Kawachi, I.; McCune, S. The pet factor--companion animals as a conduit for getting to know people, friendship formation and social support. *PLoS One* **2015**, *10*, e0122085, doi:10.1371/journal.pone.0122085.
588. Woodhead, J.K.; Feng, L.C.; Howell, T.J.; Ruby, M.B.; Bennett, P.C. Perceptions of dog breeding practices, breeding dog welfare and companion dog acquisition in a self-selected sample of Australian adults. *Animal Welfare* **2023**, *27*, 357-368, doi:10.7120/09627286.27.4.357.
589. Woodward, S.H.; Jamison, A.L.; Gala, S.; Holmes, T.H. Canine companionship is associated with modification of attentional bias in posttraumatic stress disorder. *PLoS One* **2017**, *12*, e0179912, doi:10.1371/journal.pone.0179912.
590. Worsley, H.K.; O'Hara, S.J. Cross-species referential signalling events in domestic dogs (Canis familiaris). *Anim Cogn* **2018**, *21*, 457-465, doi:10.1007/s10071-018-1181-3.
591. Wright, M.M.; Schreiner, P.; Rosser, B.R.S.; Polter, E.J.; Mitteldorf, D.; West, W.; Ross, M.W. The Influence of Companion Animals on Quality of Life of Gay and Bisexual Men Diagnosed with Prostate Cancer. *Int J Environ Res Public Health* **2019**, *16*, doi:10.3390/ijerph16224457.
592. Xin, X.; Cheng, L.; Li, S.; Feng, L.; Xin, Y.; Wang, S. Improvement to the subjective well-being of pet ownership may have positive psychological influence during COVID-19 epidemic. *Anim Sci J* **2021**, *92*, e13624, doi:10.1111/asj.13624.
593. Yates, D.; Leedham, R. Prepubertal neutering in cats and dogs. *In Practice* **2019**, *41*, 285-298, doi:10.1136/inp.l5007.
594. Yeap, I. Being towards death. *Med J Aust* **2021**, *215*, 456-457, doi:10.5694/mja2.51323.
595. Young, J.S. Pet therapy: dogs de-stress students. *J Christ Nurs* **2012**, *29*, 217-221, doi:10.1097/cnj.0b013e31826701a7.
596. Zier, E.R. Which one to follow? Service animal policy in the United States. *Disabil Health J* **2020**, *13*, 100907, doi:10.1016/j.dhjo.2020.100907.
